# HPLC-DAD-ESI/MS and 2D-TLC Analyses of Secondary Metabolites from Selected Poplar Leaves and an Evaluation of Their Antioxidant Potential

**DOI:** 10.3390/ijms26136189

**Published:** 2025-06-27

**Authors:** Loretta Pobłocka-Olech, Mirosława Krauze-Baranowska, Sylwia Godlewska, Katarzyna Kimel

**Affiliations:** Department of Pharmacognosy with Medicinal Plants Garden, Faculty of Pharmacy, Medical University of Gdańsk, Gen. J. Hallera Str. 107, 80-416 Gdańsk, Poland; miroslawa.krauze-baranowska@gumed.edu.pl (M.K.-B.); sylwia.godlewska@gumed.edu.pl (S.G.); kimkas@gumed.edu.pl (K.K.)

**Keywords:** *Populus*, flavonoids, polyphenols, phenol glycosides, HPLC-DAD-ESI/MS, 2D-TLC, bioautography, phytochemicals, medicinal plants, chromatographic identification

## Abstract

Poplar leaves (*Populi folium*) are a herbal remedy traditionally used for the treatment of rheumatic diseases and prostate inflammation. The aim of our study was a comprehensive identification of secondary metabolites occurring in the leaves of *Populus alba*, *Populus* × *candicans*, and *Populus nigra*, in order to search for a source of raw plant material rich in active compounds. Total salicylate (TSC), flavonoid (TFC), and phenolic compound (TPC) contents were determined, and the antioxidant potential was assessed using DPPH (2,2-diphenyl-1-picrylhydrazyl), ABTS (2,2′-azino-bis(3-ethylbenzothiazoline- 6-sulfonic acid) diammonium salt), and FRAP (ferric reducing antioxidant power) assays as well as 2D-TLC (two-dimensional thin layer chromatography) bioautography using DPPH, riboflavin-light-NBT (nitro blue tetrazolium chloride), and xanthine oxidase inhibition tests. Secondary metabolites present in the analyzed poplar leaves were identified under the developed HPLC-DAD-ESI/MS (high performance liquid chromatography with photodiode array detection and electrospray ionization mass spectrometric detection analysis conditions and using the 2D-TLC method. Among the 80 identified compounds, 13 were shown for the first time in the genus *Populus*. The most diverse and similar set of flavonoids characterized the leaves of *P.* × *candicans* and *P. nigra*, while numerous salicylic compounds were present in the leaves of *P. alba* and *P.* × *candicans*. All analyzed leaves are a rich source of phenolic compounds. The highest flavonoid content was found in the leaves of *P. × candicans* and *P. nigra*, while the leaves of *P. alba* were characterized by the highest content of salicylates. All examined poplar leaves demonstrated antioxidant potential in all the assays used, which decreased in the following order: *P. nigra*, *P. × candicans*, *P. alba*.

## 1. Introduction

Chronic inflammation is the basis of many diseases of civilization, including cardiovascular diseases; cancer; and metabolic syndrome, including diabetes, and their treatment is a serious challenge for public healthcare [1]. Many plant species provide plant raw materials with anti-inflammatory effects that can support the treatment process and, through rational and systematic use, prevent the development of these diseases. In addition, herbal medicines, unlike synthetic medicines, are characterized by a very low risk of side effects or their complete absence [1]. On the other hand, achieving reproducible therapeutic efficacy of a herbal medicine requires the content of active compounds to be at a minimum level or higher, specified by the standard described in the pharmacopeial monograph. Therefore, for many plant raw materials, candidates for a herbal drug, the widest possible recognition of the chemical composition, the determination of the compound/class of compounds responsible for the effect observed in pharmacological studies, and the determination of the minimum effective dose are required. Therefore, it is important to develop appropriate standardization methods for each new plant raw material, so that the drug developed using the material can meet the requirements for modern herbal medicines used in phytotherapy.

Known plant materials with anti-inflammatory effects resulting from the presence of salicylic compounds come mainly from plants belonging to two genera of the Salicaceae family, namely—*Salix* (willow) and *Populus* (poplar) [2]. Representatives of both genera most often provide the bark as a medicinal raw material. In the case of poplar, however, the additional raw material is the leaves, which are characterized by a very diverse chemical composition. In addition to salicylic derivatives, they contain flavonoids and phenolic acids, which have anti-inflammatory effects based on different mechanisms of action [1].

The presence of the above-mentioned classes of active compounds may result in synergistic action and cause a stronger therapeutic effect. The synergism of salicylic and flavonoid compounds was noted in clinical studies evaluating the anti-inflammatory effect of the extract from willow bark from the Salicaceae family [3]. Willow bark as well as poplar leaves are a rare source of particularly valuable glucosides and glucoside esters of salicylic alcohol (saligenin). The product of the metabolism of this class of natural salicylates in the human body is salicylic acid, which is formed only in the blood by the oxidation of released, free salicylic alcohol. This prevents the irritation of the gastric mucosa—one of the more common side effects observed when using synthetic salicylates [3].

In the monograph of the Polish Pharmacopoeia XIII (FP XIII) [4], medicinal plant raw materials are the leaves of the black poplar *Populus nigra*, with a flavonoid content not less than 0.6% and salicylic derivatives at a concentration not less than 0.2%. Products containing poplar leaves are practically not available on the herbal products market, because the natural resources of *P. nigra* in Poland are gradually decreasing, and the phytopharmaceutical industry has a problem with obtaining a plant raw material that meets the requirements of the FP XIII. Therefore, it is necessary to search for new sources of the plant raw material, *Populi folium*, meeting pharmacopeial standards.

In Poland, there are over 30 species and varieties of poplars, the leaves of which can be a potential source of plant raw material for pharmaceutical applications. In our previous studies [5], we demonstrated the presence of about 23 compounds from the class of polyphenols and simple phenols in the leaves of 9 species and varieties of the genus *Populus*, and determined the total content of flavonoids and salicylic derivatives. The results show that the leaves of some poplars can be an equally valuable source or are sometimes much richer in salicylic glycosides than the leaves of *P. nigra*.

The recognition of the broadest possible range of secondary metabolites, including primarily those that determine the therapeutic effect, and assessment of their content are essential before using the plant raw material as a medicinal plant raw material. The chemical composition of poplar leaves is still poorly recognized. Recent research in this area has led to the recognition of the chemical composition of *Populus tremula* × *P. tremuloides* leaves [6] and the isolation and identification of 16 compounds in *P. alba* leaves [7]. In our previous studies of secondary metabolites present in the leaves from nine different poplar species and hybrids, a number of compounds were also not identified [5]. Therefore, the aim of our study was to identify the chemical composition of the leaves of *Populus alba* and *Populus × candicans*, selected in previous studies as the richest in flavonoids and salicylates, and containing the most compounds with antioxidant activity in TLC bioautography tests, against the background of the set of active compounds from *P. nigra* leaves, using the HPLC-DAD-ESI/MS method.

The analyzed poplar leaves were quantitatively characterized by the determination of total flavonoid (TFC), total phenolic (TPC), and total salicin (TSC) contents. As it is known that oxidative stress is a common cause of inflammation [8], the antioxidant capacity of extracts from the examined leaves was determined based on DPPH (2,2-diphenyl-1-picrylhydrazyl), ABTS (2,2′-azino-bis(3-ethylbenzothiazoline-6-sulfonic acid) diammonium salt), and FRAP (ferric reducing antioxidant power) spectrophotometric assays. In addition, the 2D-TLC method for the separation of secondary metabolites present in *Populus* leaves was developed and used for the identification of compounds that exhibited antioxidant properties by DPPH, riboflavin-light-NBT, and xanthine oxidase inhibition tests.

## 2. Results

### 2.1. HPLC-DAD-ESI/MS Analysis

**Figure 1:**
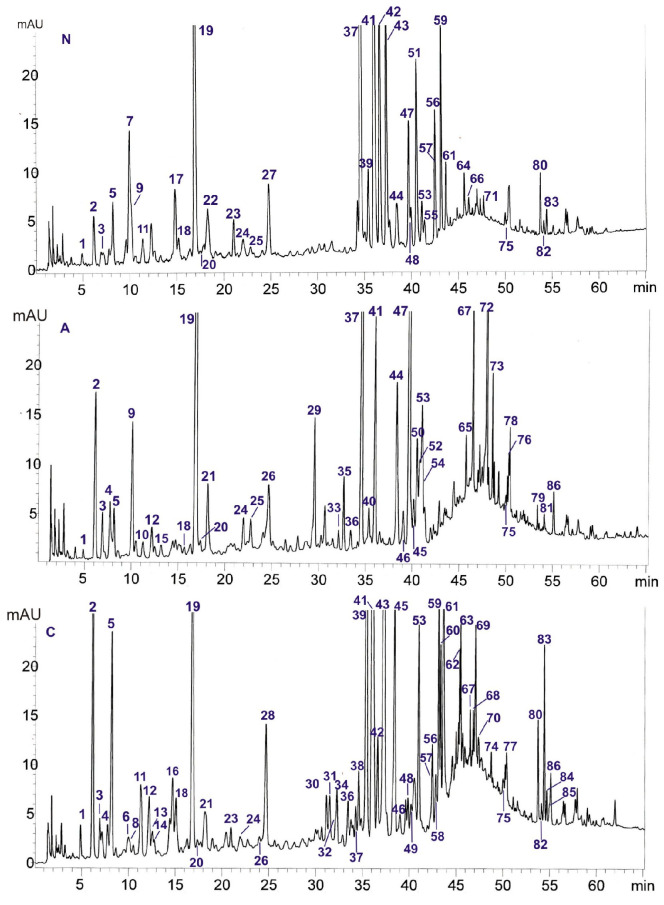
HPLC-DAD-ESI/MS chromatograms of methanol extracts from the leaves of *P. nigra* (N), *P. alba* (A), and *P. × candicans* (C). The corresponding compound numbers are presented in Table 1.

The optimization of the HPLC-DAD-ESI/MS separation of secondary metabolites occurring in the analyzed poplar leaves was carried out using several types of columns: Kinetex F5 (4.6 × 100 mm, 2.6 µm, and 2.1 × 100 mm, 1.7 µm), Kinetex C-18 (4.6 × 100 mm, 2.6 µm), and Kinetex PFP (4.6 × 100 mm, 2.6 µm, and 4.6 × 100 mm, 2.6 µm) (Phenomenex, Torrance, CA, USA). Mobile phases containing mixtures of different organic modifiers were tested: methanol, acetonitrile, or isopropanol and water with added formic acid. The HPLC best separation of compounds present in the methanol extracts was achieved on a Kinetex F5 (4.6 × 100 mm, 2.6 µm) column using mobile phases consisting of A—water:formic acid (100:0.1, *v*/*v*), B—acetonitrile:formic acid (100:0.1, *v*/*v*), and the following gradient elutions ranging from 3 to 20% B (0–40 min), 20 to 50% B (40–55 min), and 50 to 70% B (55–65 min) (Figure 1). In comparison with the previously used separation method [5], the separation of caffeic and chlorogenic acids, the separation of salicortin from co-eluted compounds, and the better separation of flavonoid compounds and salicin ester derivatives were performed.

The chemical composition of *P. nigra* (N), *P. alba* (A), and *P. × candicans* (C) leaves by the HPLC-DAD-ESI/MS method was obtained and compared. The compounds were identified in comparison to the 58 standards of polyphenols (flavonoids, flavan-3-ols) and simple phenols (phenolic acids, salicylic compounds). The identification was based on a comparison with the retention times of chromatographic peaks (t_R_), UV spectra, and calculated formulas of protonated [M+H]^+^ and deprotonated [M-H]^−^ molecular ions on the basis of ESI mass spectra. The results of the HPLC-DAD-ESI/MS analysis are presented in Table 1. We detected 86 compounds, and among them, 27 were identified in comparison with standard compounds. The other compounds were tentatively identified with the use of UV and MS spectral data (Table 1 and Table S1) and the literature data [2,6,7,9,10,11,12,13,14,15,16,17,18,19,20,21,22].

**Table 1:** Chromatographic data of the compounds present in the methanol extracts of *Populus* leaves obtained by HPLC-DAD-ESI/MS.

N^o^	t_R_ (min)	UV (λ_max_, nm)	*m*/*z* [M+H]^+^	*m*/*z* [M-H]^−^/[M-H+HCOOH]^−^	Compound	Reference	N	A	C
**1**	4.92	214, 268		169/-	Gallic acid		tr ^1^	tr ^1^	+ ^1^
**2**	6.16	211, 268		-/331	Salicin		+	++	+++
**3**	6.89	213, 274		109/-	Pyrocatechol		tr	+	+
**4**	7.72	217, 272		271, 123/-	Unidentified saligenin derivative t.a. ^2^		-	+	+
**5**	8.16	260, 296		153/-	Protocatechuic acid		+	+	+++
**6**	9.86	210, 286, 333sh		315/-	Dihydroxybenzoic acid hexoside isomer I	[16]	-	-	tr
**7**	9.94	192, 278		301/-	Unidentified compound		++	-	-
**8**	10.06	208, 300sh, 326		315/-	Dihydroxybenzoic acid hexoside isomer II	[16]	-	-	tr
**9**	10.12	221, 298sh, 326	355	353/-	Chlorogenic acid isomer I (mono-Caffeoylquinic acid)	[6,13]	++	+	-
**10**	11.24	223, 300		337/-	Coumaroylquinic acid isomer I	[6]	-	tr	-
**11**	11.36	234, 300sh, 326	181	341/-	Caffeoyl hexoside isomer I	[16,17]	+	-	+
**12**	12.21	210, 260, 300sh	165	325/-	Coumaroyl hexoside isomer I	[17]	+	+	+
**13**	12.54	206, 265, 290sh, 330sh		327/-	Acetyl-salicin	[7,9]	-	-	+
**14**	12.75	217, 294sh, 324	181	341/-	Caffeoyl hexoside isomer II	[16,17]	-	-	tr
**15**	13.21	221, 312	339	337/-	Coumaroylquinic acid isomer II	[6]	-	tr	-
**16**	14.73	217, 290, 326		417, 341/-	Unidentified compound		-	-	++
**17**	14.88	215, 265		329/-	Vanillic acid hexoside	[16]	++	-	-
**18**	15.88	226, 313	165	325/-	Coumaroyl hexoside isomer II	[17]	tr	tr	+
**19**	16.98	234, 299sh, 324	355	353/-	Chlorogenic acid		+++	+++	+++
**20**	17.48	212, 277, 326sh	291	289/-	Catechin		tr	tr	tr
**21**	18.18	248sh, 293sh, 317	355	353/-	Chlorogenic acid isomer II (mono-Caffeoylquinic acid)	[6,13]	-	+	+
**22**	18.26	212, 289sh, 320		179/-	Caffeic acid		+	-	-
**23**	21.40	254, 267sh, 349	627	625/-	Quercetin-O-dihexoside	[16]	+	-	+
**24**	22.03	230, 310	339, 165	337/-	Coumaroylquinic acid isomer III	[6]	+	tr	tr
**25**	22.81	211, 260, 306	339, 165	337/-	Coumaroylquinic acid isomer IV	[6]	+	+	-
**26**	24.66	218, 272		423/469	Salicortin	[9,12]	-	++	tr
**27**	24.73	222, 308	165	163/-	*p*-Coumaric acid		++	-	-
**28**	24.77	214, 293sh, 325	337, 165	335/-	Coumaric acid dihydroxybenzyl ester t.a.		-	-	++
**29**	29.63	251, 264sh, 354	757, 303, 611, 465	755/-	Quercetin-hexoside-di-rhamnoside	[10,11]	-	++	-
**30**	31.23	261, 302sh, 354	481, 319	479/-	Myricetin-hexoside	[6,15]	-	-	+
**31**	31.53	252, 295sh, 327		481/-	Caffeic acid derivative	[17]	-	-	+
**32**	32.23	254, 270sh, 353	611, 303	609/-	Quercetin-hexoside-rhamnoside	[13,16]	-	-	tr
**33**	32.24	262, 352	741, 449, 287	739/-	Kaempferol-hexoside-di-rhamnoside	[10,11]	-	tr	-
**34**	32.34	252, 266sh, 354	597, 303, 465	595/-	Quercetin-pentosylhexoside	[6]	-	-	+
**35**	32.80	253, 352	771, 317, 625, 479	769/-	Isorhamnetin-hexoside-di-rhamnoside	[10,13]	-	+	-
**36**	33.49	226, 287sh, 326		343/-	Hydrocaffeic acid hexoside	[16]	-	tr	tr
**37**	34.6	254, 264sh, 304sh, 356	611, 465, 303	609/-	Quercetin 3-O-rutinoside (rutin)		+++	+++	tr
**38**	34.74	248, 297sh, 330		439/-	Caffeic acid derivative	[17]	-	-	+
**39**	35.41	253, 266sh, 302sh, 350	465, 303		Quercetin 3-O-galactoside (hyperoside, hyperin)		+	-	+++
**40**	35.44	217, 240sh, 307		405/451	Salicyloyl-salicin	[9,12]	-	+	+
**41**	36.04	254, 266sh, 300sh, 351	465, 303	463/-	Quercetin 3-O-glucoside (isoquercitrin)		+++	++	+++
**42**	36.65	251, 263sh, 346	449, 287	447/-	Luteolin 7-O-glucoside (cynaroside, luteoloside)		++	-	+
**43**	37.41	253, 264sh, 346	463, 287	461/-	Luteolin 7-O-glucuronide		++	-	+++
**44**	38.46	263, 287, 351	595, 287	593/-	Kaempferol 3-O-rutinoside (nicotiflorin)		+	++	-
**45**	38.64	254, 267sh, 300sh, 350	435, 303	433/-	Quercertin-3-O-arabinoside (guaiaverin)		-	tr	++
**46**	39.25	227, 312		423/-	Grandidentatin	[6,7]	-	+	tr
**47**	39.67	253, 295sh, 352	625, 479, 317	623/-	Isorhamnetin-rutinoside	[7,13]	+	+++	-
**48**	39.52	266, 295sh, 346	449, 287	447/-	Kaempferol 3-O-glucoside (astragalin)		tr	-	tr
**49**	40.47	197, 313		423/-	Grandidentatin isomer I	[6,21]	-	-	tr
**50**	40.59	212, 296sh, 326		447/-	Populoside/populoside A	[17,22]	-	+	-
**51**	40.78	252, 266sh, 300sh, 353	449, 303	447/-	Quercetin 3-O-rhamnoside (quercitrin, quercitroside, quercimelin)		++	-	-
**52**	40.85	219,274		-/435	Tremuloidin	[9,12]	-	+	-
**53**	41.06	253, 267sh, 300sh, 351	479, 317	477/-	Isorhamnetin-3-O-glucoside		tr	+	++
**54**	41.13	234, 294sh, 329	517	515/-	1,5-Dicaffeoylquinic acid		-	++	-
**55**	41.33	265, 340	433, 271	431/-	Apigenin-7-O-glucoside (Apigetrin)		tr	-	-
**56**	42.47	266, 333	447	445, 269/-	Apigenin-glucuronide	[20]	++	-	+
**57**	42.65	251, 265sh, 347	463, 301	461/-	Trihydroxy-methoxyflavone-hexoside	[10]	+	-	+
**58**	42.92	218, 318		423/469	Grandidentatin isomer II	[6,19,21]	-	-	tr
**59**	43.16	249, 266sh, 346	477	475/-	Trihydroxy-methoxyflavone-glucuronide	[20]	++	-	++
**60**	43.44	216, 321		461/-	Populoside C	[22]	-	-	++
**61**	43.65	213, 296sh, 325		447/-	Populoside/populoside A	[17,22]	+	-	+++
**62**	45.43	215, 315	287	487, 285/-	Unidentified salicin derivative isomer I t.a.	[9]	-	-	+
**63**	45.55	218, 315		487/-	Unidentified salicin derivative isomer II t.a.	[9]	-	-	+
**64**	45.65	268, 306	255, 417	-/461	Chrysin-hexoside	[18]	+	-	-
**65**	45.77	271, 336	273	449/-	Unidentified compound		-	+	-
**66**	46.13	213, 269		-/435	Tremuloidin isomer	[9]	tr	-	-
**67**	46.5	217, 296sh, 327		585, 423/469	Caffeoyl-salicortin t.a.	[6]	-	++	+
**68**	46.88	198, 311		471, 327/-	Unidentified acetyl-salicin/fragilin derivative isomer I t.a.	[9]	-	-	+
**69**	47.13	218, 310		471/-	Unidentified acetyl-salicin/fragilin derivative isomer II t.a.		-	-	++
**70**	47.44	200, 313		405, 473, 501/-	Unidentified salicyloylsalicin/salireposide/siebolside derivative t.a.	[9]	-	-	tr
**71**	47.71	251, 265sh, 344	287	285/-	Luteolin		+	-	-
**72**	47.95	220, 271		527/573	Tremulacin		-	+++	-
**73**	48.59	270, 316	579, 271	577/-	Apigenin-coumaroyl-hexoside isomer I	[14]	-	+	-
**74**	48.82	280, 334sh		461/-	Unidentified compound		-	-	+
**75**	50.04	267, 335	271	269/-	Apigenin		tr	tr	tr
**76**	50.35	225, 271, 318		423, 527/469, 573	Tremulacin isomer	[9]	-	+	-
**77**	50.45	267, 368	287	285/-	Kaempferol		-	-	+
**78**	50.47	268, 316	579	577/-	Apigenin-coumaroyl-hexoside isomer II	[14]	-	+	-
**79**	53.43	218, 272		443, 387/-	Unidentified salicylate-like compound t.a.		-	tr	-
**80**	53.83	266, 315	255	253/-	Chrysin		+	-	+
**81**	54.16	209, 273sh, 313		509/-	Salicyloyl tremuloidin t.a.	[2]	-	tr	-
**82**	54.18	289, 331sh	257	255/-	Pinocembrin		tr	-	tr
**83**	54.49	263, 316sh, 360	271	269/-	Galangin		+	-	++
**84**	54.74	212, 240, 337		289/-	Unidentified compound		-	-	tr
**85**	55.03	266, 367	301	299/-	Trihydroxy-methoxyflavone	[20]	-	-	tr
**86**	55.14	200, 257	557	555/-	Unidentified compound		-	+	+

^1^ Peak intensity of compound, observed at λ—254 nm: +++, high; ++, medium; +, low; tr., traces—not detected; ^2^ t.a.—tentatively identified; leaves of: *P. nigra* (N), *P. alba* (A), and *P. × candicans* (C).

#### 2.1.1. Identification of Flavonoids

The analysis carried out with the use of standards revealed in the subclass of flavonols the presence of isoquercitrin (compound **41**), rutin (**37**), and isorhamnetin-3-O-glucoside (**53**) in the leaves of all analyzed poplars; hyperoside (**39**), astragalin (**48**), chrysin (**80**), and galangin (**83**) in *P. nigra* and *P. × candicans*; guaiaverin (**45**) in *P. alba* and *P. × candicans*; nicotiflorin (**44**) in *P. alba* and *P. nigra*; quercitrin (**51**) only in *P. nigra*; and kaempferol (**77**) only in *P. × candicans*. In the subclass of flavones, apigenin (**75**) was identified in all analyzed poplars, cynaroside (**42**) and luteolin-7-O-glucuronide (**43**) in *P. nigra* and *P. × candicans*, and apigetrin (**55**) and luteolin (**71**) only in *P. nigra*. In the flavanones subclass, pinocembrin (**82**) was present in the leaves of *P. nigra* and *P. × candicans* (Table 1).

Moreover, on the basis of UV spectra and *m*/*z* values of molecular [M+H]^+^ and [M-H]^−^ ions in the ESI mass spectra in the positive and negative modes and the literature data (Ferreres et al., 2015 [10]; Kachmar et al., 2019 [11]; Kleszken et al., 2022 [13]; Król-Kogus et al., 2014 [14]; Matsuda and Matsuo, 1985 [15]; Nosrati Gazafroudi et al., 2024 [16]; Pearl and Darling, 1971a [18]; Rusalepp et al., 2021 [6]; Tawfeek et al., 2019 [7]; Tsagkaris et al., 2022 [20]), 15 flavonoids were identified, for which probable structures are presented (Table 1). Compound **23**, whose peak in the HPLC chromatograms of *P. nigra* and *P.* × *candicans* extracts was at t_R_ 21.40 min, was characterized by absorption maxima at 254, 267sh nm (II maximum), and 349 nm (I maximum) in the UV spectrum. The presence of a shoulder in the second absorption maximum indicates the presence of an o-dihydroxyl group in the side phenyl [23], characteristic of flavonols. This compound produced molecular ions in the positive mode [M+H]^+^ at *m*/*z* 627 and in the negative mode [M-H]^−^ at *m*/*z* 625, which correspond to the mass of quercetin-O-di-hexoside [16,23]. The peak of this compound had a different retention time (t_R_ 21.40 min) than that of the quercetin-3,4′-O-diglucoside (t_R_ 29.31 min) used as a standard, but probably it was the quercetin-3,7-O-diglucoside previously described in the leaves of *P. angustifolia* and *P. × acuminata* [24]. Compound **30**, present in the leaves of *P. × candicans*, showed molecular ions in the positive mode [M+H]^+^ at *m*/*z* 481 and in the negative mode [M-H]^−^ at *m*/*z* 479. Moreover, it exhibited fragmentation ions in the positive mode [M+H]^+^ at *m*/*z* 319 by the loss of a hexoside moiety (162 Da), indicating the presence of myricetin as an aglycone (Ag) [6,16]. Previously, myricetin-3-O-galactoside was identified in the leaves of this hybrid [15]. The similar UV spectra (251, 264sh, 354 nm) showed compound **29** on the chromatograms of *P. alba* (t_R_ 29.63 min) and **32** from *P. × candicans* (t_R_ 32.23 min). Compound **29** showed molecular ions in the positive mode [M+H]^+^ at *m*/*z* 757 and fragment ions at *m*/*z* 611 [M+H-rhamnose]^+^ and *m*/*z* 465 [M+H-di-rhamnose]^+^, resulting from the loss of one (146 Da) and two (292 Da) rhamnose moieties, respectively. Additionally, this compound yielded the [Ag+H]^+^ ion at *m*/*z* 303, consistent with the mass of quercetin. On the basis on the literature data, it can be stated that compound **29** is quercetin-hexoside-di-rhamnoside [10,11]. Compound **32** exhibited the same fragmentation ion for aglycones (*m*/*z* 303) and molecular ions in the positive mode [M+H]^+^ at *m*/*z* 611 and in the negative mode [M-H]^−^ at *m*/*z* 609. The structure of compound 32 was established as quercetin-hexoside-rhamnoside [13,16].

The obtained ESI-MS data for compounds **33** and **35** present in *P. alba* reveal that they have three sugars in their structures. Compound **33** had, as dominating, the molecular ion [M+H]^+^ at *m*/*z* 741 and fragmentation ions at *m*/*z* 449 [M+H-di-rhamnose]^+^ and *m*/*z* 287 [Ag+H]^+^, resulting from the loss of the di-rhamnose moiety (292 Da) and hexose (162 Da), respectively, identifying kaempferol as an aglycone; therefore, compound **33** was characterized as kaempferol-hexoside-di-rhamnoside [10,11]. The presence of kaempferol in the structure of compound **33** was confirmed by the UV spectrum, characterized by the presence of two absorption maxima at 262 nm and 352 nm [23]. Compound **35** presented a molecular ion [M+H]^+^ at *m*/*z* 771 and three fragmentation ions. Two of them were created by the loss of one moiety of rhamnose (146 Da) at *m*/*z* 625 [M+H-rhamnose]^+^ and two moieties of rhamnose at *m*/*z* 479 [M+H-di-rhamnose]^+^. The mass of the last fragment ion at *m*/*z* 317 [Ag+H]^+^ corresponds to the mass of isorhamnetin. On the basis of these data, compound **35** was characterized as isorhamnetin-hexoside-di-rhamnoside (Ferreres et al., 2015 [10]).

The MS spectrum of another flavonoid detected in the extract from *P. × candicans,* compound **34**, exhibited molecular ions in the positive mode [M+H]^+^ at *m*/*z* 597 and in the negative mode [M-H]^−^ at *m*/*z* 595. In the positive mode, it presented a fragmentation ion at *m*/*z* 465 [M+H-pentose]^+^ and an ion at *m*/*z* 303 [Ag+H]^+^ that originated from an aglycone. Similarly, as was shown for compound **23**, in the UV spectrum of compound **34**, we observed a shoulder at the II absorption maximum, which confirms the presence of quercetin as an aglycone; therefore, it was classified as quercetin-pentosylhexoside [6]. Compound **85** showed molecular ions in the positive mode [M+H]^+^ at *m*/*z* 301 and in the negative mode [M-H]^−^ at *m*/*z* 299, corresponding to trihydroxy-methoxyflavone [20].

In the leaves of *P. nigra* and *P. alba*, compound **47** was detected, which exhibited molecular ions in the positive mode [M+H]^+^ at *m*/*z* 625 and in the negative mode [M-H]^−^ at *m*/*z* 623. In the positive mode, it has fragmentation ions at *m*/*z* 479 [M+H-rhamnose]^+^ and at *m*/*z* 317 [Ag+H]^+^, which corresponds to isorhamnetin as an aglycone. On the basis of these data, compound **47** was classified as isorhamnetin-rutinoside [7,13]. Compound **64** was present in the last of the mentioned species, which showed molecular ions in the positive mode [M+H]^+^ at *m*/*z* 417 and a fragmentation ion at *m*/*z* 255 [Ag+H]^+^, which corresponds to dihydroxyflavone as an aglycone. In the negative mode of ionization, we observed a molecular ion at *m*/*z* 461, and it was classified as adduct [M-H^+^ 46]^−^ of dihydroxyflavone hexoside. According to the fact that chrysin was previously recognized as dihydroxyflavone in the leaves of *Populus deltoides*, it was proposed that this compound could be chrysin-hexoside [18].

On the basis of the UV spectrum (249, 266sh, 346 nm) and molecular ions in the positive mode [M+H]^+^ at *m*/*z* 477 and in the negative mode [M-H]^−^ at *m*/*z* 475 noted for compound **59**, the presence of trihydroxy-methoxy-flavone glucuronide was observed in the leaves of *P. nigra* and *P. × candicans* [20,23]. Compound **57** showed a similar UV spectrum (251, 265sh, 347 nm), and it exhibited molecular ions in the positive mode [M+H]^+^ at *m*/*z* 463 and in the negative mode [M-H]^−^ at *m*/*z* 461. This compound yielded a fragmentation ion in the positive mode at *m*/*z* 301 [Ag+H]^+^, corresponding to trihydroxy-methoxy-flavone as an aglycone; therefore, it was classified as trihydroxy-methoxy-flavone hexoside [10]. This compound was detected in the analyzed leaves of *P. alba* and *P. × candicans*. In both of these poplars, compound **56** was also present with molecular ions in the positive mode [M+H]^+^ at *m*/*z* 447 and in the negative mode [M-H]^−^ at *m*/*z* 445. It produced a fragmentation ion in the negative mode of ESI-MS ionization by the loss of the glucuronide moiety (176 Da) at *m*/*z* 269 [Ag-H]^−^. The UV spectrum of this compound possesses two absorption maxima at 266 nm and 333 nm, characteristic of flavones without an o-dihydroxyl group inside phenyl [23]. On the basis of these data, compound **56** was classified as apigenin glucuronide [20]. Compounds **73** and **78** present in *P. alba* showed similar UV spectra with two absorption maxima at 270 nm and 316 nm, which is characteristic of flavonoid esters (Mabry et al., 1970 [23]). Moreover, the presence of a molecular ion in the positive mode of ionization [M+H]^+^ at *m*/*z* 579 and a fragmentation ion at *m*/*z* 271 [Ag+H]^+^ confirms that both compounds can be isomers of apigenin-coumaroyl-hexoside [14].

#### 2.1.2. Phenolic Acids

- Hydroxybenzoic acids

Gallic (compound **1**) and protocatechuic (**5**) acids were revealed by a comparison to standard compounds in all the analyzed poplars (Table 1). Compounds **6** and **8** showed similar UV spectra and the presence of a molecular ion [M+H]^+^ at *m*/*z* 315, indicating the occurrence of dihydroxybenzoic acid hexoside isomers in the leaves of *P. × candicans* [16]. Compound **17**, present in the extract of *P. nigra,* showed a molecular ion [M+H]^+^ at *m*/*z* 329, and it can be attributed to vanillic acid hexoside [16].

- Hydroxycinnamic acids and their derivatives

The HPLC-DAD-ESI/MS analysis performed with the use of standard compounds, in the subclass of hydroxycinnamic acids, showed the presence of chlorogenic acid (**19**) in all the analyzed poplars and 1,5-dicaffeoylquinic acid (**54**) in *P. alba* and *P. × candicans*, while caffeic (**22**) and *p*-coumaric (**27**) acids were only present in the leaves of *P. nigra* (Table 1). Compounds **9** and **21** exhibited characteristic UV spectra with an absorption maximum at 326 nm and shoulder at 298sh nm, and revealed molecular ions [M+H]^+^ at *m*/*z* 355 and [M-H]^−^ at *m*/*z* 353 and a fragment ion at *m*/*z* 315 [M+H—quinic acid]^+^, which proves the presence of chlorogenic (mono-caffeoylquinic) acid isomers [6,13].

Compounds **10**, **15**, **24**, and **25** showed the same molecular ions [M-H]^−^ at *m*/*z* 337 in the negative mode. Additionally, compounds **24** and **25** in the positive mode yielded a molecular ion [M+H]^+^ at *m*/*z* 339 and a fragment ion [M+H—quinic acid]^+^ at *m*/*z* 165, which corresponds to the mass of coumaric acid (164 Da). Finally, compounds **10**, **15**, **24**, and **25** were identified as coumaroylquinic acid isomers [6]. Compounds **10** and **15** were present in *P. alba* leaves and **24** in all the analyzed *Populus* extracts, while **25** was present only in *P. alba* and *P. nigra.* Compounds **11** (t_R_ 11.36 min) and **14** (t_R_ 12.75 min) exhibited a molecular ion [M-H]^−^ at *m*/*z* 341 and a fragmentation ion [M+H-caffeoyl]^+^ at *m*/*z* 181, and their structures were assigned to caffeoyl hexoside isomers [16,17]. The first set was identified in the plant material of *P. nigra* and *P. × candicans*, whereas the second set was identified exclusively in *P. × candicans*. In the ESI-MS spectra of compounds **12** and **18**, observed in the HPLC chromatograms of all analyzed *Populus* leaves, at peaks **12** and **18**, there were molecular ions [M-H]^−^ at *m*/*z* 325. Both compounds produced a fragmentation ion at *m*/*z* 165 by the loss of the hexose moiety [M+H-hexose]^+^; so, they were classified as coumaroyl hexosides [17].

Another phenolic acid hexoside may be compound **36**, since the molecular ion [M-H]^−^ is present in the ESI/MS spectrum at *m*/*z* 343. Furthermore, its UV spectrum shows an absorption maximum value characteristic of this class. Compound **36** is classified as a hydrocaffeic acid hexoside [16]. Compounds **31** and **38**, detected in the *P. × candicans* extract, showed absorption maximum values in the UV region of 327–330 nm and presented ESI-MS spectrum molecular ions [M+H]^+^ at *m*/*z* 481 and 439, respectively. Based on these data and taking into consideration some information from the literature [17], they were classified as caffeic acid derivatives. In the extract of *P. × candicans*, compound **28** showed a UV spectrum characteristic of hydroxycinnamic acid’s absorption maximum at 325 nm, with a shoulder at 293 (sh) and a molecular ion [M-H]^−^ at *m*/*z* 335. In the positive mode of ionization, it produced a molecular ion [M+H]^+^ at *m*/*z* 337 and a fragmentation ion at *m*/*z* 165, proving the presence of a coumaroyl moiety in the structure of this compound. The *m*/*z* value of the molecular ion of compound **28** is consistent with the molecular mass of the dihydroxybenzoic acid coumaric ester (Table 1).

#### 2.1.3. Salicylate Glycosides

In this class of glycoside derivatives, compound **2** was identified as salicin and compound **72** as tremulacin by comparison with the standards. The first compound occurred in all analyzed poplars, while the second compound only in *P. alba* (Table 1).

Most salicylate glycosides exhibit two characteristic regions of UV absorption—210–220 nm and 265–275 nm. Moreover, due to the presence of formic acid in the mobile phase, they often form [M-H+HCOOH]^−^ adducts in the negative-ionization ESI-MS mode, which provides additional information for their identification by HPLC-DAD-ESI/MS. On the basis of characteristic UV spectra and molecular ions [M-H]^−^ or adducts [M-H+HCOOH], the following derivatives of salicylic alcohol were tentatively identified: 2′acetyl-salicin (compound **13**, [M-H]^−^ at *m*/*z* 327) [9] in *P. × candicans*, salicortin (compound **26**, [M-H]^−^ at *m*/*z* 423) [12] in *P. alba* and *P. × candicans*, tremuloidin (compound **52**, [M-H+HCOOH]^−^ at *m*/*z* 435) [9] in *P. alba*, and its isomer (compound **66**, [M-H+HCOOH]^−^ at *m*/*z* 435) in *P. nigra*.

Compound **76**, detected only in *P. alba*, produced, in the negative mode, ions at *m*/*z* 527 and *m*/*z* 423 next to their adducts with HCOOH at *m*/*z* 573 and *m*/*z* 469, and showed a UV spectrum with absorption maximum values of 225 nm and 271 nm, which are characteristic of salicylic compounds. On this basis, it was recognized as an isomer of tremulacin (Abreu et al., 2011 [9]). Compound **40**, present in extracts of *P. alba* and *P. × candicans*, revealed a molecular ion [M-H]^−^ at *m*/*z* 405 next to the adduct with HCOOH (*m*/*z* 451). Its UV spectrum with absorption maximum values at 217 nm, 240sh nm, and 307 nm confirms that it is a salicylic compound. Based on the obtained chromatographic data, compound **40** was tentatively identified as salicyloyl-salicin [9,12]. Absorption maxima in the UV region of 312–318 nm and a molecular ion [M-H]^−^ at *m*/*z* 423 were observed for peaks **46**, **49**, and **58**. On the basis of the literature data [6,7,21], these compounds were characterized as grandidentatin isomers. They were detected in *P. × candicans*, and the first of them, compound **46**, was also found in *P. alba*. In turn, compounds **50** and **61** showed absorption maximum values in the UV region of 325–326 and a molecular ion [M-H]^−^ at *m*/*z* 447, which corresponds to the literature data for populoside or populoside A [17,22]. The former was detected in the leaves of *P. alba*, and the latter in the other analyzed poplars. Similar UV spectra and molecular ion [M-H]^−^ values at *m*/*z* 461 were exhibited by compound **60**, observed on *P. × candicans* chromatograms, and it was identified as populoside C [22]. In the extracts of leaves of *P. alba* and *P. × candicans,* compound **67** was present, which had a molecular ion [M-H]^−^ at *m*/*z* 585 and fragment ions at *m*/*z* 423 and 469. The difference between the mass (162 Da) of a molecular ion and fragment ion [M-H-caffeoyl]^−^ and its adduct with formic acid [M-H-caffeoyl+HCOOH]^−^, corresponding to salicortin, tentatively enabled the identification of this compound as caffeoyl-salicortin. *P. alba* leaves contain compound **81**, which has the UV absorption maximum characteristics of some salicylic glycosides—209, 273sh, 313 nm—and produces a molecular ion [M-H]^−^ at *m*/*z* 509, and it was classified as salicyloyl-tremuloidin [2].

Moreover, several compounds—**4**, **62**, **63**, **68**, **69**, **70**, and **79**—were observed with absorption maximum values in the UV spectrum characteristic of salicylic alcohol derivatives, but none of the molecular ions released by these compounds corresponded to any of the known, natural, salicylic glycosides. Compound **4** was detected in the extracts of *P. alba* and *P. × candicans*, compound **79** in *P. alba*, and the remaining compounds **62**, **63**, **68**, **69**, and **70** in *P. × candicans.* Compounds **4** and **79** showed UV spectra with the characteristics of salicylate’s absorption maximum values at 217 and 272 nm; therefore, compound **79** was characterized as an unidentified salicylate-like compound. In addition, compound **4** exhibited a fragment ion at *m*/*z* 123 corresponding to saligenin (salicylic alcohol) and was identified as an unidentified saligenin derivative. The rest of the above-mentioned compounds, namely **62**, **63**, **68**, **69**, and **70**, had absorption maximum values in the ranges of 198–218 nm and 310–315 nm. Compounds **62** and **63** had molecular ions in the negative mode [M-H]^−^ at *m*/*z* 487 and a fragment ion at *m*/*z* 285, corresponding to the salicin moiety, and they were classified as isomers of an unidentified salicin derivative. Compounds **68** and **69** showed a molecular ion in the negative mode [M-H]^−^ at *m*/*z* 471, and in addition, compound **68** yielded a fragment ion at *m*/*z* 327, corresponding to acetyl-salicin or fragilin moieties; so, these compounds were characterized as isomers of an unidentified acetyl-salicin/fragilin derivative. Considering the fact that, in salicin derivatives, the acetyl group is located in different positions, e.g., 2′- and 6′-, its location could not be determined based on the obtained data. In turn, compound **70** exhibited a fragment ion in the negative mode [M-H]^−^ at *m*/*z* 405, corresponding to salicyloylsalicin, salireposide, or siebolside moieties, and was classified as an unidentified derivative of them [2,9] (Table 1).

#### 2.1.4. Other Compounds

Moreover, besides the main class of compounds, pyrocatechol (compound **3**) and catechin (compound **20**) were identified through a comparison of the standards. Both compounds were present in all the analyzed *Populus* taxa (Table 1).

### 2.2. Quantitative Analysis of Active Compounds

The quantitative analysis of salicin in the examined poplar leaves was carried out by the pharmacopeial HPLC method of crude methanol extracts (free salicin—FS) and after alkaline hydrolysis (total salicin content—TSC). Significant differences in the salicin content were found among the tested poplar leaves. The concentration of free salicin in the analyzed methanol extracts ranged from 1.40 to 11.09 mg/g DM (dry matter), while the total salicin content ranged from 4.42 to 36.16 mg/g DM. The highest content of FS was determined in the leaves of *P. × candicans* (11.09 mg/g DM), while the highest TSC was found in the leaves of *P. alba* (36.16 mg/g DM) (Table 2).

The total content of flavonoids (TFC) was established by spectrophotometric methods and expressed in quercetin (QE) and rutin (RE) equivalents. In turn, the total phenolics content (TPC) was expressed in gallic acid equivalents (GAEs). There were no statistically significant differences in the total phenolic content in the leaves of all the analyzed poplars, and they were characterized by a similar concentration of flavonoids.

### 2.3. Two-Dimensional-TLC Analysis and Antioxidant Bioautographic Tests

**Figure 2:**
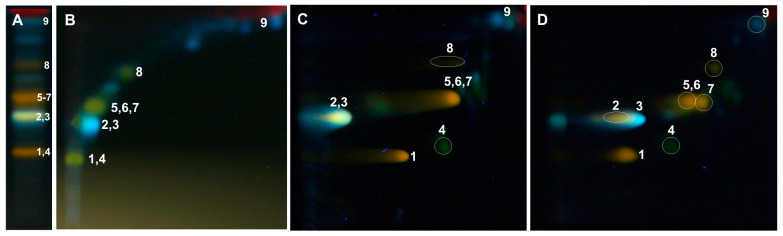
One-dimensional and two-dimensional-TLC separation of methanol extract from the leaves of *Populus nigra* (optimization). Adsorbent: silica gel 60 F_254_; mobile phase 1D: (**A**) ethyl acetate/ethyl-methyl ketone/water/formic acid (50:30:10:10, *v*/*v*/*v*/*v*); 2D: (**B**) diethyl ether/water/formic acid (18:10:10, *v*/*v*/*v*); (**C**) chloroform/methanol/water (70:30:4, *v*/*v*/*v*), (**D**) chloroform/methanol/water/formic acid (70:30:2:2, *v*/*v*/*v*/*v*); detection: NPR/PEG, UV λ-366 nm, lined with chromatography paper (6 cm) and in a saturated (7 min) chamber. **1**—rutin, **2**—luteolin-7-O-glucuronide, **3**—chlorogenic acid, **4**—nicotiflorin, **5**—isoquercitrin, **6**—isorhamnetin-3-O-glucoside, **7**—cynaroside, **8**—quercitrin, and **9**—caffeic acid.

The separation of compounds present in the analyzed methanol extracts from poplar leaves was carried out under the optimized 2D-TLC method on silica gel plates with a mixture of anhydrous formic acid:water:methylethyl ketone:ethyl acetate (1:1:3:5; *v*/*v*/*v*/*v*) as the mobile phase in the first dimension (1D) and a mixture of chloroform/methanol/water/formic acid (70:30:2:2, *v*/*v*/*v*/*v*) in the second dimension (2D). TLC plates were derivatized with the use of a solution of 2-aminoethyl diphenylborinate (NPR) (10 g/L) in methanol and a solution of Macrogol 400 (PEG) (50 g/L) in methanol, and then, they were analyzed under UV light at λ-366 nm (Figure 2D).

**Figure 3:**
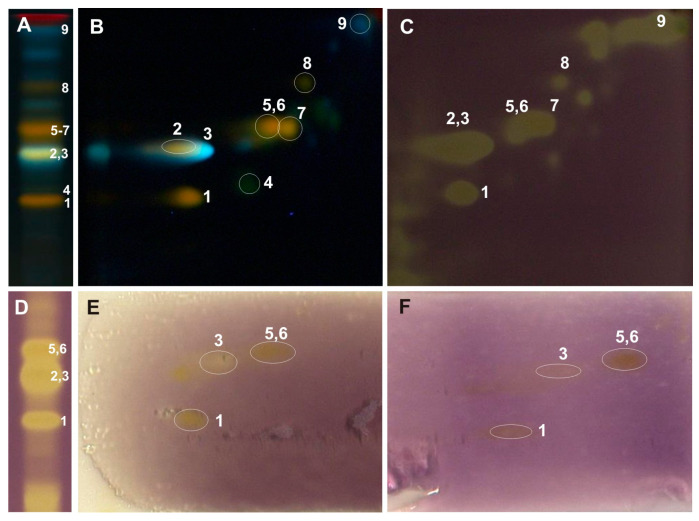
One-dimensional and two-dimensional-TLC separation of methanol extract from the leaves of *Populus nigra*. Adsorbent: silica gel 60 F_254_; mobile phase 1D: ethyl acetate/ethyl-methyl ketone/water/formic acid (50:30:10:10, *v*/*v*/*v*/*v*); 2D: chloroform/methanol/water/formic acid (70:30:2:2, *v*/*v*/*v*/*v*); lined with chromatography paper (6 cm) and in a saturated (7 min) chamber. (**A**,**B**) NPR/PEG, UV λ-366 nm; (**C**,**D**) 0.05% DPPH; (**E**) riboflavin-light-NBT test; (**F**) xanthine oxidase inhibition test. **1**—rutin, **2**—luteolin-7-O-glucuronide, **3**—chlorogenic acid, **4**—nicotiflorin, **5**—isoquercitrin, **6**—isorhamnetin-3-O-glucoside, **7**—cynaroside, **8**—quercitrin, **9**—caffeic acid.

**Figure 4:**
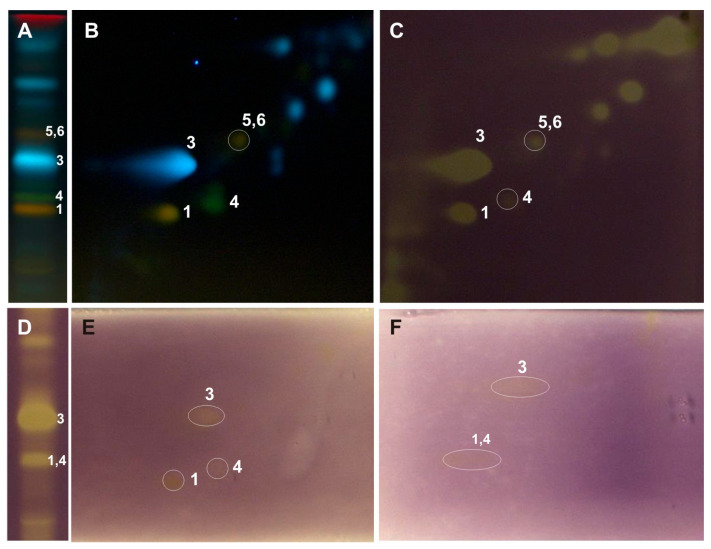
One-dimensional and two-dimensional-TLC separation of methanol extract from the leaves of *P. alba*. The chromatographic separation conditions and compound numbers are described in Figure 3. Adsorbent: silica gel 60 F_254_; mobile phase 1D: ethyl acetate/ethyl-methyl ketone/water/formic acid (50:30:10:10, *v/v/v/v*); 2D: chloroform/methanol/water/formic acid (70:30:2:2, *v/v/v/v*); lined with chromatography paper (6 cm) and in a saturated (7 min) chamber. (**A**,**B**) NPR/PEG, UV λ-366 nm; (**C**,**D**) 0.05% DPPH; (**E**) riboflavin-light-NBT test; (**F**) xanthine oxidase inhibition test. **1**—rutin, **3**—chlorogenic acid, **4**—nicotiflorin, **5**—isoquercitrin, **6**—isorhamnetin-3-O-glucoside.

**Figure 5:**
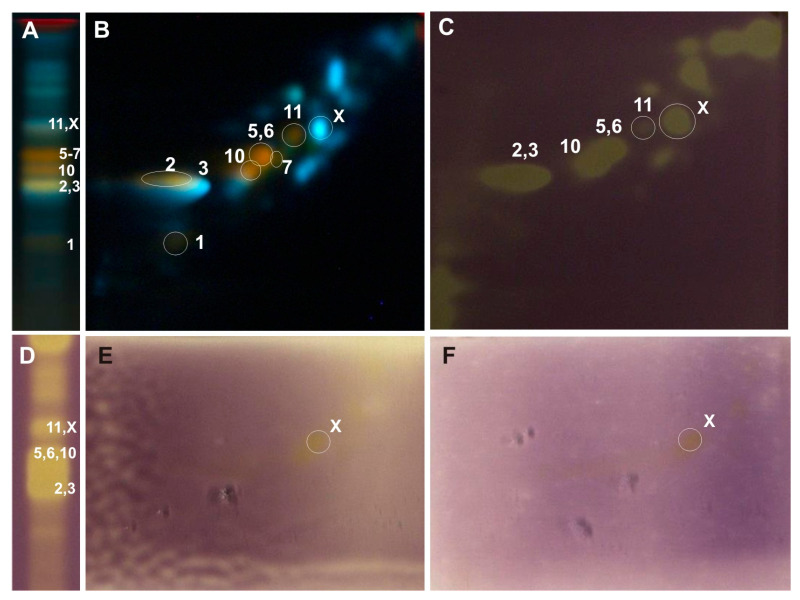
One-dimensional and two-dimensional-TLC separation of methanol extract from the leaves of *P. × candicans*. The chromatographic separation conditions and compound numbers are described in Figure 3. Adsorbent: silica gel 60 F_254_; mobile phase 1D: ethyl acetate/ethyl-methyl ketone/water/formic acid (50:30:10:10, *v/v/v/v*); 2D: chloroform/methanol/water/formic acid (70:30:2:2, *v/v/v/v*); lined with chromatography paper (6 cm) and in a saturated (7 min) chamber. (**A**,**B**) NPR/PEG, UV λ-366 nm; (**C**,**D**) 0.05% DPPH; (**E**) riboflavin-light-NBT test; (**F**) xanthine oxidase inhibition test. **1**—rutin, **2**—luteolin-7-O-glucuronide, **3**—chlorogenic acid, **5**—isoquercitrin, **6**—isorhamnetin-3-O-glucoside, **7**—cynaroside, **10**—hyperoside, and **11**—guaiaverin, **X**—unidentified compound.

The above-mentioned 2D-TLC conditions enabled the identification of rutin and chlorogenic acid in all the examined *Populus* extracts: luteolin-7-O-glucuronide and luteolin-7-O-glucoside in the leaves of *P. nigra* and *P. × candicans* and nicotiflorin in *P. alba* and *P. nigra*. The presence of quercitrin and caffeic acid was confirmed in the leaves of *P. nigra* and hyperoside alongside quercetin-3-O-arabinoside in the extract of *P. × candicans*. Moreover, in the obtained 2D-TLC chromatograms of all the analyzed extracts, isoquercitrin and isorhamnetin-3-O-glucoside were observed as one orange spot (Figure 3, Figure 4 and Figure 5).

The developed 2D-TLC method was applied to assess the antioxidant properties of compounds present in the investigated poplar extracts with the use of DPPH, riboflavin-light-NBT, and xanthine oxidase inhibition bioautographic tests (Figure 3, Figure 4 and Figure 5). Among the compounds observed on the 2D-TLC chromatograms, rutin (*P. nigra* and *P. alba*), chlorogenic acid (*P. nigra* and *P. alba*), and nicotiflorin (*P. alba*) were the compounds that showed antioxidant properties in all the bioautographic tests used (Figure 3 and Figure 4), and luteolin-7-O-glucuronide (*P. nigra* and *P. × candicans*), quercitrin (*P. nigra*), caffeic acid (*P. nigra*), hyperoside, guaiaverin, and chlorogenic acid (*P. × candicans*) by the DPPH test (Figure 3 and Figure 5). Moreover, antioxidant activity was shown for the mixture of isoquercitrin and isorhamnetin-3-O-glucoside in all the analyzed *Populus* extracts using the DPPH test and by riboflavin-light-NBT and xanthine oxidase inhibition tests only in *P. nigra* leaves (Figure 3, Figure 4 and Figure 5).

### 2.4. Antioxidant Capacity

The antioxidant capacity of the studied poplar leaves extracts was estimated using DPPH, ABTS, and FRAP spectrophotometric assays, and was expressed in mM Trolox (6-hydroxy-2,5,7,8-tetramethylchroman-2-carboxylic acid—TEA) per 1 g of DM of the tested plant material (Table 3).

The highest activity was determined for *Populus nigra* leaves by all the tests used: DPPH—0.91 mM TEA/g DM, FRAP—6.36 mM TEA/g DM, and ABTS—2.51 mM TEA/g DM. There were no statistically significant differences in the antioxidant capacity of the extracts from *P. alba* and *P. × candicans* in the FRAP test, respectively, 4.82 and 5.16 mM TEA/g DM, and for the leaves from *P. nigra* and *P. × candicans* by the ABTS test, respectively, 2.51 and 2.18 mM TEA/g DM. The lowest activity was established for the extract from *P. alba* leaves by the DPPH assay—0.49 mM TEA/g DM (Table 3).

## 3. Discussion

The results of the conducted research using the HPLC-DAD-ESI/MS method significantly expand the knowledge of the chemical composition of the leaves of three representatives of the *Populus* genus, namely *P. nigra*, *P. alba*, and *P. × candicans*. They provide new data and serve to verify the existing knowledge. They indicate both the similarities and differences in the profiles of secondary metabolites among the individual taxa studied. They confirm the presence of compounds from the flavonoids, phenolic acids, and salicylic derivatives in the poplar leaves as a constant set, characteristic of this plant material. The results of the conducted qualitative and quantitative studies confirm the previous literature data that show that flavonoid compounds, alongside salicylic glycosides and phenolic acids, constitute one of the main classes of compounds occurring in poplar leaves. In order to quantitatively characterize the analyzed leaves from various *Populus* taxa, the total content of flavonoids, salicylic derivatives, and phenols was determined. The content of individual compounds was discussed by comparing the intensity of their corresponding peaks in the HPLC-DAD chromatograms. Moreover, against the background of these results, the antioxidant potential of leaves was assessed both by spectrophotometric methods (DPPH, ABTS, and FRAP assays) and by using TLC bioautography, emphasizing the importance of the antioxidant properties of plant raw materials in the context of their anti-inflammatory activity.

### 3.1. Identification of Secondary Metabolites and the Determination of Their Content

#### 3.1.1. Flavonoids

The qualitative analysis based on the obtained chromatograms of methanolic extracts showed that, among flavonoids, glycoside forms of derivatives from the flavonol subclass predominate in all the tested poplars. The presence of significant amounts of isoquercitrin (**41**) was confirmed in all the analyzed poplar species and hybrids (Figure 1, Figure 2, Figure 3, Figure 4 and Figure 5). Moreover, quercetin (**37**), isorhamnetin (**47**), and kaempferol (**44**) rutinosides were found in high concentrations in the leaves of *P. alba* and *P. nigra*. It is worth noting that the presence of kaempferol-3-O-rutinoside (nicotiflorin) was first demonstrated in the *Populus* genus. Among kaempferol monoglycosides, only the presence of 3-O-glucoside—astragalin (**48**)—was confirmed in the leaves of *P. nigra* and *P. × candicans* (Table 1) [5].

*P. nigra* leaves, in comparison with those of other poplars, were characterized by a high content of quercitrin (**51**), while *P. × candicans* leaves were characterized by a high content of hyperoside (**39**) and guaiaverin (**45**). This last compound was first detected in the leaves of this species. Previously, the presence of guaiaverin was observed in the leaves of *Populus deltoides* [25] and *P. tremula* [26]. Similarly to our previous studies [5], significant amounts of isorhamnetin-3-O-glucoside (**53**) were found in the leaves of *P. × candicans*. This compound was also present in other taxa, but at significantly lower concentrations. In turn, for the first time in the genus *Populus,* the presence of rare-in-nature tri-glycosides, hexoside-di-rhamnoside derivatives of quercetin (**29**), kaempferol (**33**), and isorhamnetin (**35**), was identified only in *P. alba* leaves. Previously, the presence of isorhamnetin-glucosyl-di-rhamnoside has been described in the leaves of mistletoe (*Viscum album* subsp. *album*) growing on the *P. alba* tree [13]. The occurrence of this type of compound may constitute an important chemotaxonomic feature of the species, distinguishing it from other examined poplars. Other interesting di-glycosides—quercetin-hexoside-rhamnoside (**32**) and quercetin-pentosylhexoside (**34**)—were established in this study in the leaves of *P. × candicans.* Isorhamnethin-glucosyl-rhamnoside was previously identified in mistletoe, which is hosted by the poplar tree [13], while kaempferol-O-pentosylhexoside was previously identified in the leaves of *Populus tremula × tremuloides* [6] and *P. tremula* [26]. Moreover, within the subclass of flavonol di-glycosides, the presence of quercetin-dihexoside (**23**) was also detected in the analyzed plant material of *P. × candicans* and *P. alba*. It was classified as quercetin-3,7-O-diglucoside because its peak on the HPLC chromatogram had a different t_R_ than the 3,4′-O-diglucoside of quercetin used as a standard, and the occurrence of this compound was previously described in the leaves of *P. angustifolia*, *P. ×acuminata*, *P. sargentii*, and *P. wislizenii* [24]. In addition, in this subclass of flavonoids, the presence of myricetin-3-O-galactoside (**30**) was confirmed in the leaves of *P. × candicans* [15]. Among the aglycones from the flavonols subclass, galangin (**83**) was found in the leaves of *P. nigra* and *P. × candicans*, and kaempferol (**77**) in the leaves from the latter taxon (Figure 1 and Table 1).

The next subclass of flavonoids occurring in poplars is flavones, among which the presence of apigenin (**75**) was confirmed in all the analyzed extracts: cynaroside (**42**) and chrysin (**80**) were present in the leaves of *P. nigra* and *P. × candicans,* and apigetrin (**55**) and luteolin (**71**) were confirmed in *P. nigra* (Figure 1 and Table 1) [5]. In relation to the literature and spectral analysis data, compound **64** detected in the leaf extract of *P. nigra* can probably be classified as chrysin-7-O-glucoside [18], and compound **85** present in *P. × candicans* may be chrysoeriol [20]. The former compound was previously described as a component of *P. deltoides* leaves [18], while the latter compound occurred in the leaf buds of *P. × canadensis* var. *robusta* [27]. Moreover, in this subclass of derivatives, in comparison to standard, luteolin-7-O-glucuronide (**43**) was identified in the analyzed plant material of *P. nigra* and *P. × candicans*. In addition, in both poplars, on the basis of UV and MS spectra and the literature data, glucuronides of apigenin (**56**) and trihydroxy-methoxy-flavone (**59**) as well as hexoside (**57**) [10,20,23] in the last-mentioned flavones were detected. In turn, only in the leaves of *P. alba* was the presence of two isomers of apigenin-coumaroyl-hexoside (compounds **73** and **78**) [14] identified. These compounds were, for the first time, detected in the genus *Populus* (Figure 1 and Table 1).

In the subclass of flavanones, in the examined poplar leaves, only small amounts of pinocembrin (**82**) were found in *P. nigra* and *P. × candicans* (Figure 1 and Table 1). This compound is widespread in the genus *Populus*, especially in the leaf buds of poplars [28], but it was reported for the first time in *P. × candicans*.

The pharmacopeial method for determining the content of flavonoid compounds in poplar leaves comprises a colorimetric assay of the sum of compounds after the acid hydrolysis of acetone extract, expressed in quercetin equivalents. However, in most studies [29,30,31], the flavonoid content in plant materials is determined in crude extracts obtained using mixtures of various concentrations of alcohol and water. Both methods determine the absorption of the flavonoid complex with aluminum chloride. Therefore, in this study, the flavonoid content in leaves was determined using the method described in the FP XIII monograph [4] and the method described by Kalita et al. [30]. The latter method was selected based on the assessment of the repeatability of the results obtained from the tests using various methods, described previously in studies on the phytochemical analysis of various plant matrices [29,30,31].

The content of flavonoids determined by the pharmacopeial method expressed in quercetin equivalents ranged from 6.23 mg/g DM for *P. alba* leaves to 8.69 mg/g DM in the material from *P. × candicans* (Table 2). The determined concentrations were similar to those obtained previously for another batch of leaves of the same species and poplar variety [5], and met the requirements of FP XIII (Table 2).

In the second method, described by Kalita et al. [30], the content of flavonoid compounds was expressed in rutin equivalents and ranged from 12.38 mg/g DM for *P. alba* leaves to 17.02 mg/g DM for *P. nigra* leaves (Table 2). Similar levels of flavonoids were described for poplar leaves collected in Hungary (12.82–15.10 mg/g DM) [32], while in leaves of *P. tremula* from Romania, the content of flavonoids, determined using a similar method, was much higher and amounted to 31.74 mg/g DM [29].

#### 3.1.2. Phenolic Acids

The analyses performed provided new information on the type of phenolic acids from the subclass of hydroxybenzoic derivatives occurring in the *Populus* genus. Gallic (**1**) and protocatechuic (**5**) acids were detected in all the poplars examined, as well as hexosides of dihydroxybenzoic acid (**6** and **8**) in *P. × candicans* and vanillic acid (**17**) in *P. nigra* (Figure 1 and Table 1), the occurrence of which, to the best of our knowledge, has not yet been described in the analyzed poplar species and hybrids. The presence of gallic and protocatechuic acids has been previously described in the leaves of *P. ×canadensis* and *P. deltoides* [33].

A more diverse set of compounds was represented by hydroxycinnamic derivatives, among which chlorogenic acid (**19**) was dominant in all the extracts tested (Figure 1). In addition, the presence of caffeic (**22**) and *p*-coumaric (**27**) acids was confirmed in *P. nigra* leaves. In this subclass of derivatives, we noted some significant differences in relation to our previous studies [5]. The last two phenolic acids mentioned above were also identified previously in other poplars studied, whereas chlorogenic acid was not found in the leaves of *P. × candicans*. In the present study, in comparison with the reference substance, the presence of 1,5-dicaffeoylquinic acid (**54**) was detected for the first time in the *Populus* genus in the leaves of *P. alba* and *P. × candicans* (Table 1).

Moreover, in this subclass of phenolic acids, on the basis of the obtained UV and MS spectral data, two chlorogenic acid isomers (compounds **9** and **21**), four coumaroylquinic acid isomers (compounds **10**, **15**, **24**, and **25**), and hexosides of caffeic (compounds **11** and **14**), coumaric (compounds **12** and **18**), and hydrocaffeic (compound **36**) acids were tentatively identified. Both isomers of caffeoylquinic acid were present in the leaves of *P. alba*: the first one (**9**) was detected in the *P. nigra* material, while the second one (**21**) was detected in *P. × candicans* (Figure 1 and Table 1). All coumaroylquinic acid isomers were present in *P. alba*, two of them, **24** and **25**, in *P. nigra* and only one of them, **24**, in *P. × candicans*. These coumaroylquinic acid derivatives were previously observed in the leaves of *P. tremula × tremuloides* [6]. Glycosides of coumaric acid were present in all the analyzed poplars, whereas hexosides of caffeic acid were present in *P. nigra* and *P. × candicans*. The collected literature data on the chemical composition of poplars indicate that glycoside derivatives of phenolic acids are widely identified within this genus. Coumaroylglucosides were previously detected in the leaves of *P. balsamifera, P. deltoides*, and *P. tremula* var. *davidiana* [34,35,36] and in the bark of *P. balsamifera, P. tremula, P. tremula* var. *davidiana*, and *P. tremuloides* [22,37,38], while caffeoylglucoside was detected in the leaves of *P. nigra* [38]. In most studies on the chemical composition of poplar leaves, derivatives of *p*-coumaric acid were identified [19,34,36], but based on the MS and UV data obtained in this study, the position of the hydroxyl group in the structure of coumaric acid could not be determined. However, the hydrocaffeic acid hexoside (**36**) observed in the present study in the material from *P. alba* and *P. × candicans* has not been described in poplars to date. The UV and MS data of compounds **31** and **38** are consistent with those of the compounds described as caffeic acid derivatives, which were previously defined as components of poplar buds [17]. In turn, compound **28**, tentatively identified as a coumaric acid dihydroxybenzyl ester, has not been described in the literature to date; so, the confirmation of its structure requires further studies.

The content of all phenolic compounds present in the analyzed poplar leaves was assessed as the total phenolic content (TPC) using the Folin–Ciocalteau reagent and expressed as GAEs. There were no statistically significant differences among the taxa studied, and the TPC ranged from 81.75 to 85.29 mg/g DM GAE (Table 2). To date, significant differences in the contents of total phenolic compounds have been demonstrated among different poplar species and their hybrids. Similar contents were characterized by the leaves of *Populus × euramericana* syn. *P. × canadensis* (53.63–73.73 DM mg/g) [32], and *P. trichocarpa × deltoides* (97.8 mg/g DM) [39], whereas significantly higher amounts of TPC were detected in the leaves of *P. tremula* (136.5–146.71 mg/g DM) [29] compared to the very low amounts detected in *P. deltoides* (16.49–21.67 mg/g DM) and *P. × canadensis* (20.93 mg/g DM), grown in a greenhouse [33].

#### 3.1.3. Salicylate Glycosides

Salicin and its ester derivatives, such as salicortin, are known anti-inflammatory components of poplar leaves. Their main in vivo metabolites, salicylic acid and catechol, inhibited inflammatory markers in various in vitro tests [40].

Among the studied taxa, the most diverse sets of salicylic compounds were found in the leaves of *P. alba* (9 compounds) and *P. × candicans* (10 compounds). The presence of salicin (**2**) was confirmed in the leaves of all analyzed poplars, salicortin (**26**) in *P. × candicans* and *P. alba*, as well as grandidentatin (**46**), tremulacin (**72**), and tremuloidin (**52**) in the last of the mentioned species [5,7]. In addition, populoside (**50**) was tentatively identified for the first time in *P. alba* and its isomer (**61**) with t_R_ 43.65 min in *P. nigra* and *P. × candicans*; salicyloyl-salicin (**40**) and caffeoyl-salicortin (**67**) in *P. nigra* and *P. × candicans*; 2′-acetyl-salicin (**13**), populoside C (**60**), and grandidentatin (**46**) with its two isomers (49 and 58) in *P. × candicans*; a tremuloidin isomer (**66**) in *P. nigra* and a tremulacin isomer (**76**) in *P. alba* (Table 1). The most frequently detected salicylic compounds in poplar leaves include salicin, populin, salicortin, tremuloidin, and tremulacin [2,5,9,38]. The presence of populosides in the leaves of *P. deltoides, P. suaveolens, P. tremula,* and *P. tremula* var. *davidiana* [19,35,36]; grandidentatin in *P. alba, P. tremula* var. *davidiana,* and *P. suaveolens* [19,35,38]; 2′-acetyl-salicin in *P. alba* and *P. tremula* [7,9]; and salicyloylsalicin in *P. balsamifera, P. deltoides, P. tremula*, and *P. trichocarpa* [9,34,36] has been described to date.

The content of salicin, both in free and bound forms, was determined to be significantly different, depending on the taxon from which the leaves were obtained. The highest content of free salicin (FS) was found in the leaves of *Populus × candicans* (11.09 mg/g DM) and the lowest in *Populus nigra* (1.40 mg/g DM). On the other hand, the sum of salicylic derivatives (TSC) determined after alkaline hydrolysis to release salicin from its ester derivatives was the highest in the leaves of *P. alba* (36.16 mg/g DM) and the lowest in the plant material from *P. nigra* (4.42 mg/g DM) (Table 2). Similar relationships were observed in previous studies of leaves collected from the same species and varieties of *Populus* [5]. All the analyzed leaves met the pharmacopeial requirements for the content of total salicylic derivatives (TSC), which should be no less than 2 mg/g (0.2%) [4]. The literature data confirm a significant variation in the salicin content in the leaves of various taxa of the *Populus* genus. Free salicin was present in the leaves of *P. tremula* (0.03–2.4 mg/g DM) and *P. tremula × tremuloides* (1.7–7.13 mg/g DM) in a range similar to that determined in the analyzed poplars [9,41,42]. On the other hand, the contents of sum salicylic compounds determined to date in poplar leaves sometimes reached values more than twice as high as those determined in the analyzed plant material, i.e., *P. tremula*, 19.4 mg/g DM [43], and *P. tremula* × *tremuloides*—27.19–84.45 mg/g DM [41,42]. It is worth noting that significant differences in the salicin content have been found to date in the leaves of trees belonging to the same species or variety. This confirms the need to assess the content of salicylic derivatives in the plant material for pharmaceutical purposes.

The most diverse and similar set of flavonoid compounds characterized the leaves of *P. × candicans* and *P. nigra*, where they occurred in the highest concentrations (8.69 and 8.12 mg/g QE). In turn, a high content of salicylic compounds, several-times higher compared to their content in the leaves of *P. nigra* (4.42 mg/g), and a more qualitatively diverse set of salicylic glycosides were revealed in the leaves of *P. alba* (36.16 mg/g) and *P. × candicans* (21.47 mg/g). A number of differences revealed in the chemical composition of the analyzed poplar leaves may have chemotaxonomic significance and may facilitate the botanical identification of plant material intended for medicinal purposes. The presence of quercitrin, luteolin, chrysin-hexoside (probably chrysin-7-O-glucoside), *p*-coumaric acid, caffeic acid, and vanillic acid hexoside was revealed only in *P. nigra* leaves. The leaves of *P. alba* were distinguished by the presence of tremulacin, tremuloidin and its isomer, hexoside-di-rhamnosides of quercetin, kaempferol and isorhamnetin, isomers of apigenin-coumaroyl-hexoside, and 1,5-dicaffeoylquinic acid. However, only populoside C, myricetin-hexoside (probably myricetin-3-O-galactoside), quercetin-hexoside-rhamnoside, quercetin-pentosylhexoside, and kaempferol were identified in the leaves of *P. × candicans*.

### 3.2. Evaluation of Antioxidant Potential

#### 3.2.1. Two-Dimensional-TLC Bioautography

The next stage of this study was the evaluation of the antioxidant activity of the tested *Populus* extracts as an element of their anti-inflammatory potential. A quick, simple, and relatively cheap method of assessing the biological properties of the components of plant extracts is TLC bioautography. As a result of the chromatographic separation of the components of the tested plant matrix on the adsorbent layer, TLC bioautography allows for the assessment of their properties, e.g., antioxidant, and, as a result, the assessment of the activity of the entire matrix [44].

In the 1D-TLC separation conditions described in FP XIII as a method for confirming the phytochemical identity of the plant raw material, *Populi folium*, the co-elution of compounds from the flavonoid class and phenolic acids, e.g., cynaroside and isoquercitrin, and luteolin-7-O-glucuronide and chlorogenic acid, was observed (Figure 2A). Therefore, conditions for the separation of groups of active compounds from the poplar leaves analyzed by two-dimensional thin-layer chromatography (2D-TLC) were developed.

In the 2D-TLC separations in the first dimension of separation on silica gel plates, the conditions described in the pharmacopeia monograph for *Populi folium* were used, while the composition of the mobile phase used in the second dimension was optimized. In the optimization process, mixtures of chloroform, methanol, ethyl acetate, ethyl ether, isopropanol, and tetrahydrofuran with the addition of water or formic acid as the eluent components were used (Figure 2).

As a result of the preliminary tests, the best separation was obtained using a solvent mixture consisting of chloroform, methanol, and water in a ratio of 70:30:4 (*v*/*v*/*v*) (Figure 2C) as the mobile phase in the second dimension. To reduce the effect of spot blurring of separated compounds observed in the chromatograms, half of the volume of water in the mobile phase was replaced with formic acid. This allowed for a better separation of co-eluting compounds, namely luteolin-7-O-glucuronide from chlorogenic acid and isoquercitrin from cynaroside (Figure 2D). The separations were carried out under the conditions of saturation in the chromatographic chamber with mobile phase vapors (cellulose chromatographic paper placed inside the chamber and wetted with the mobile phase used; saturation: 7 min), which increased the concentration of spots of the separated compounds and, as a result, increased their resolution.

Among the various derivatization reagents tested, constituting group reagents for flavonoids and other phenols, the NPR/PEG reagent was selected for further chromatographic analysis [4,45,46]. The largest number of spots of separated compounds with intense fluorescence was observed in TLC chromatograms obtained after spraying the compounds with the NPR/PEG reagent, under the influence of which the spots of flavonoid compounds—fluorescing yellow and orange—differed from the spots of phenolic acids and their derivatives, fluorescing intensely blue (Figure 2).

In the developed 2D-TLC chromatographic system, the metabolic profiles of the examined poplar leaves were analyzed, and bioautographic tests were performed to determine the antioxidant activity by the DPPH method, light riboflavin-NBT, and xanthine oxidase inhibition (Figure 3, Figure 4 and Figure 5). The TLC bioautography method revealed the presence of compounds showing antioxidant activity through the inhibition of DPPH, riboflavin-light-NBT, and xanthine oxidase in all the tested leaves. Their identification was carried out in relation to the standard compounds and is described in the Results Section. Moreover, in the obtained 2D-TLC chromatograms of the *P. × candicans* extract, compound X was observed, with intense yellow fluorescence, in all the bioautography tests used, which indicates its strong antioxidant activity (Figure 5). Therefore, further studies are planned, including its isolation and the determination of its chemical structure.

The developed 2D-TLC separation conditions can be used to confirm the phytochemical identity of a poplar leaf and rapidly assess its antioxidant potential.

#### 3.2.2. Antioxidant Capacity

Free radical scavenging reduces the risk of inflammation; therefore, assessing the antioxidant properties of a plant extract is one method to assess its potential to reduce inflammation [8]. Spectrophotometric analyses using DPPH, FRAP, and ABTS assays enabled the quantitative assessment of the total potential of the tested extracts from poplar leaves expressed as mM of Trolox equivalents (TEA) per 1 g DM (Table 3). The greatest differences in the strength of the antioxidant activity were found using the DPPH assay—0.49 mM TEA/g DM for *P. alba* and 0.91 mM TEA/g DM for *P. nigra*. In the remaining tests, the differences were smaller—ABTS, 1.81–2.51 mM TEA/g DM, and FRAP, 4.82–6.36 mM TEA/g DM. To sum up the results obtained from the conducted tests, it was found that the antioxidant potential of the analyzed extracts from the poplar leaves decreased in the following order: *P. nigra* > *P. × candicans* > *P. alba*.

When analyzing the literature data, it should be emphasized that the antioxidant potential of the leaves of the poplar species *P. tremula* [29], *P. × euramericana* (syn. *P. × canadensis*) [32], *P. alba* [7], and *P. × canadensis* and *P. detloides* [33] have been determined to date using the DPPH, FRAP, ABTS, and TEAC tests. Considering the fact that the antioxidant potential was expressed using different units, e.g., IC_50_ values, AAE (ascorbic acid equivalents)/g DM, and others, it is difficult to assess the antioxidant potential of the leaves studied against the background of these data.

The noted antioxidant effect of the tested poplar leaves may have an impact on their anti-inflammatory activity. To date, the anti-inflammatory effect of the methanol extract of *P. deltoides* leaves was observed in a LPS(lipopolysaccharide)-stimulated mouse macrophage cell line RAW 264.7 at doses of 12.5 and 25 µg/mL [47]. The extract inhibited the expression of inducible nitric oxide synthase (iNOS) and nitric oxide (NO) production and caused a significant reduction (*p* < 0.01) in the levels of pro-inflammatory factors: tumor necrosis factor α (TNF-α), IĸBα (NF-κB inhibitor α), NF-ĸB-p65 (nuclear factor ĸB-p65), and NF-ĸB (nuclear factor ĸB). It was found that the anti-inflammatory effect of the extract may be related to the inhibition of iNOS expression through the suppression of JNK (c-Jun N-terminal kinase) and p38 MAPK (mitogen-activated protein kinase) signaling and the inhibition of NF-κB activation.

In 40 clinical studies on its efficacy in the therapy of degenerative and inflammatory rheumatic diseases, a significant and low-to-moderate efficacy of Phytodolor (STW extract), a herbal medicine whose main component (60%) is an extract of poplar bark and leaves, was observed [48]. It was found, among other things, that therapy with this herbal medicine is effective compared to the placebo and comparable to diclofenac, indomethacin, and piroxicam administered concurrently. In addition, it was well-tolerated by the study participants and revealed a low risk of side effects, especially compared to non-steroidal anti-inflammatory drugs (NSAIDs). At the same time, the mechanism of anti-inflammatory action of individual extracts contained in its composition was investigated using in vitro tests. *P. tremula* bark and leaf extracts modulated 51 genes involved in the immunoregulation, inflammation, and apoptosis of human fibroblasts, compared to 31 genes modulated by *Fraxinus excelsior* bark extract and 24 genes modulated by the *Solidago virgaurea* herb. The poplar extract significantly reduced the proinflammatory cytokines interleukin-13 (IL-13) and tumor necrosis factor (TNF-α), and the gene expression of proinflammatory IL-6 and IL-8, monocyte chemoattractant protein 1 (MCP-1), and growth-regulated oncogene alpha (Gro-α). Moreover, it inhibited proinflammatory TNF-α gene expression and the synthesis of TNF-α and COX-2 (cyclooxygenase-2) proteins in IFN(interferon)-c/LPS-stimulated human monocytes [48].

## 4. Materials and Methods

### 4.1. Chemicals

All solvents were of analytical grade. Methanol, ethyl acetate, chloroform, acetone, ethylenediaminetetraacetic acid (EDTA), diethyl ether, sodium hydroxide (NaOH), sulfuric acid (VI) 95% (H_2_SO_4_), formic acid 98–100%, Folin–Ciocalteu’s phenol reagent, sodium nitrate, and sulfanilic acid were obtained from POCH (Gliwice, Poland); methanol was used for spectroscopy (Uvasol); hydrochloric acid (HCl) 37%, sodium sulphate anhydrous, and magnesium acetate tetrahydrate were obtained from Merck (Darmstadt, Germany); aluminum chloride hexahydrate was obtained from AlfaAesar (Kandel, Germany); methenamine (urotropine) was obtained from PPH Galfarm (Kraków, Poland); and methylethyl ketone was obtained from Chem-Lab (Zedelgem, Belgium). Acetic acid 99.8%, sodium acetate, sodium carbonate, sodium persulfate, *p*-anisaldehyde, 2,2′-azino-bis(3-ethylbenzothiazoline-6-sulfonic acid) diammonium salt (ABTS), 2,4,6-Tris(2-pyridyl)-s-triazine (TPTZ), iron (III) chloride hexahydrate (FeCl_3_ × 6H_2_O), (±)-6-hydroxy-2,5,7,8-tetramethylchromane-2-carboxylic acid (Trolox) 2-aminoethyl diphenylborinate (Natural Product Reagent—NPR), polyethylene glycol 400 (Macrogol 400—PEG 400), 2,2-diphenyl-1-picrylhydrazyl (DPPH), nitro blue tetrazolium chloride (NBT), phosphate buffer (1.0 M, pH = 7.4), agar, allopurinol, riboflavin, xanthine oxidase (XO—from bovine milk, ammonium sulfate suspension), and xanthine were purchased from Sigma-Aldrich (Steinheim, Germany). Chromatography paper grade 3 was obtained from Whatman (Little Chalfont, United Kingdom). Acetonitrile Lichrosolv^®^, methanol Lichrosolv^®^, and isopropanol Lichrosolv^®^ were purchased from Merck (Darmstadt, Germany). Formic acid LC-MS LichroPur 97.5–98.5% was obtained from Sigma-Aldrich (Steinheim, Germany). Ultra-pure water for LC-MS analysis was prepared using the Direct-Q^®^ Water Purification System (Merck Millipore; Darmstadt, Germany).

Galangin, isorhamnetin, luteolin, pinocembrin, pinostrobin, genkwanin, eriodictyol, tectochrysin, chrysin 5,7-dimethylether, pinocembrin 5,7-dimethylether, apigenin-7-O-glucoside (apigetrin), luteolin-7-O-glucoside (cynaroside), luteolin-3′,7-di-O-glucoside, kaempferol-3-O-glucoside (astragalin), kaempferol-3-O-glucuronide, quercetin-3-O-glucuronide, quercetin-3-O-rutinoside (rutin), isorhamnetin-3-O-glucoside, quercetin-7-O-glucoside (quercimeritrin), quercetin-3,4′-di-O-glucoside, quercetin-3-O-(6-acetylglucoside), chlorogenic acid, and picein were purchased from Extrasynthèse (Genay, France); apigenin, kaempferol, myricetin, quercetin, quercetin-3-O-galactoside (hyperoside), quercetin-3-O-glucoside (isoquercitrin), ferulic acid, and caffeic acid were purchased from Fluka (Buchs, Switzerland); chrysin, pinobanksin, salicin, quercetin-3-O-rhamnoside (quercitrin), kaempferol-3-O-galactoside (trifolin), luteolin-7-O-glucuronide, kaempferol-3-O-rutinoside (nicotiflorin), gallic acid, protocatechuic acid, vanillic acid, 1,3-dicaffeoylquinic acid (cynarin), 1,5-dicaffeoylquinic acid, 3,4-dicaffeoylquinic acid, 3,5-dicaffeoylquinic acid, 4,5-dicaffeoylquinic acid, rosmarinic acid, and 1,2-dihydroxybenzene (pyrocatechol) were purchased from Sigma (St. Louis, MO, USA); quercetin-3-O-xylopyranoside (reynoutrin), kaempferol-3-O-arabinoside, pinostrobin chalcone (2′,6′-dihydroxy-4′-methoxychalcone), caffeic acid phenethyl ester from ChemFaces (Wuhan, China), catechin, naringenin, and *p*-coumaric acid were purchased from Koch-Light (Colnbrook, UK); quercetin-3-O-arabinoside (guaiaverin) was purchased from PhytoLab (Vestenbergsgreuth, Germany); and populin and tremulacin were purchased from Apin Chemicals (Abingdon, UK). The standards were dissolved in methanol (1 mg/2 mL).

### 4.2. Plant Material

The leaves of 3 species and hybrids of *Populus,* namely *P. alba* L., *P. nigra* L., and *P. × candicans* Aiton (syn. *Populus balsamifera* var. *candicans* (Aiton) A.Gray), were collected in June 2016 from trees growing in the city of Gdańsk (Poland) and then dried. The plant material was botanically classified by Jolanta Zarembska, a taxonomist from the Medicinal Plants Garden of the Medical University of Gdańsk (Poland). Voucher specimens (16L-011, 16L-013, and 16L-017) were deposited at the Department of Pharmacognosy in the Medical University of Gdańsk.

### 4.3. Sample Preparation

Dried poplar leaves (1 g) were crushed manually and then extracted three times with methanol (30 mL, 60 °C) on a magnetic stirrer (45 min). The extracts obtained were evaporated to dryness and then dissolved in methanol (25 mL). Three parallel extract samples were prepared and analyzed.

### 4.4. HPLC-DAD-ESI/MS Analysis

The analysis was carried out with an HPLC-DAD-ESI/MS Shimadzu system (Shimadzu Corp., Kyoto, Japan), which consists of two pumps LC-20AD, a degasser DGU-20A5, a semi-micro mixer, an autosampler SIL-20AC_XR_, a column oven (CTO-20AC), a controller (CBM-20A), a nitrogen generator (PEAK Scientific GeniusXE35 230), a single-quadrupole mass detector (LCMS-2020) equipped with an electrospray ionization (ESI) interface (operated in the positive and negative modes), and a diode array (DAD) SPD-M20A detector. MS analysis was performed at scanning ranges from *m*/*z* 200 to 800 and from *m*/*z* 100 to 350. The operating conditions for MS analysis were DL temperature at 250 °C, heat block temperature at 200 °C, nebulizing gas flow (N_2_) of 1.5 L/min, drying gas flow of 15 L/min, detector voltage of 1.9 kV, and interface voltage of 4.5 kV. Data acquisition was performed with the software LabSolutions version 5.89 (Copyrights© 2022–2016 Shimadzu Corp., Kyoto, Japan). Separation was performed on a Kinetex F5 column (4.6 × 100 mm, 2.6 µm) (Phenomenex, Torrance, CA, USA) using gradient elution in the following mixture of mobile phases: A—water:formic acid (100:0.1, *v*/*v*) and B—acetonitrile:formic acid (100:0.1, *v*/*v*), according to the following program ranging from 3 to 20% B (0–40 min), 20 to 50% B (40–55 min), and 50 to 70% B (55–65 min). The column temperature was set at 20 °C. The flow rate of the mobile phase was 0.8 mL/min. The injection volume was 2 (scanning *m*/*z* 200–800) or 6 µL (scanning *m*/*z* 100–350). UV detection was λ—254 nm.

### 4.5. Quantitative Analysis of Salicin

The quantitative analysis of free salicin and its ester derivatives (total salicin content—TSC) using the HPLC-DAD method was performed according to the method described in the monograph *Populi folium* of the Polish Pharmacopoeia XI [4], and their content was expressed in mg of salicin equivalent per g of dry matter (DM).

### 4.6. Quantitative Analysis of Flavonoids

The analysis of total flavonoid content (TFC) was performed according to the method described in the monograph of poplar leaves *Populi folium* in the Polish Pharmacopoeia XI [4] and expressed as mg of quercetin equivalent (QE) per g of dry matter (DM). At the same time, the TFC was determined using the method described by Barman et al. [49] and expressed as mg of rutin equivalent (RE) per g of DM.

### 4.7. Quantitative Analysis of Phenolics

Total phenolic content (TPC) was determined using the Folin–Ciocalteu method [50] and was expressed as mg gallic acid (GAE) per g of dry matter (DM).

### 4.8. One-Dimensional and Two-Dimensional-TLC and Bioautography

The methanol extracts (9 μL) were applied to TLC Silica gel 60 F_254_ plates (10 cm × 10 cm, Merck, Darmstadt, Germany) by a semi-automatic TLC sampler AS-30 (Desaga, Germany) as bands (7 mm) or spots (5 mm). Chromatograms were developed in a Twin-Trough Chamber (Camag, Muttenz, Switzerland) with a mixture of anhydrous formic acid:water:methylethyl ketone:ethyl acetate (1:1:3:5; *v*/*v*/*v*/*v*) as the mobile phase in the first dimension (1D) and a mixture of chloroform/methanol/water/formic acid (70:30:2:2, *v*/*v*/*v*/*v*) in the second dimension (2D). While the plate was developed in the first dimension, the chamber was lined with chromatography paper (6 cm) and saturated (7 min). The separation distance in each dimension was 5 cm. The derivatization of the obtained chromatograms was performed using a Derivatizer (Camag, Muttenz, Switzerland) automated spray device, and an NPR solution in methanol (10 g/L) was used as the spraying reagent, followed by a PEG 400 solution in methanol (50 g/L) [4]. After derivatization, 2D-TLC chromatograms were analyzed under UV light at λ—366 nm.

In the process of optimizing the separation conditions using the 2D-TLC method, various derivatization reagents described in the literature as group reagents for the analysis of flavonoid compounds and other phenols were tested, namely a 1% methanolic solution of magnesium acetate, 2% methanolic solution of aluminum (III) chloride, 0.5% anisaldehyde solution in a 5% methanolic sulfuric acid solution, a 1% methanolic solution of NPR and then a 5% methanolic solution of PEG 400, and diazotized sulfanilic acid [4,45,46].

Bioautography using the DPPH, riboflavin-light-NBT, and xanthine oxidase inhibition tests was carried out using the previously described procedures [51]. The documentation of chromatograms and bioautograms was performed by a TLC Visualizer (Camag, Muttenz, Switzerland).

### 4.9. Antioxidant Capacity Assays

Antioxidant capacity was measured using the DPPH, ABTS, and FRAP assays, according to the previously described procedures [52]. The results of these assays are expressed as mM Trolox equivalents per g of dry matter (mM TEA/g DM). Absorption in all the above-described assays was measured using a UV-1800 spectrophotometer (Shimadzu, Kyoto, Japan).

### 4.10. Statistical Analysis

The mean difference among the salicin content, TFC, TPC, and the DPPH, ABTS, and FRAP antioxidant capacity was controlled using a one-way analysis of variance (ANOVA) followed by Tukey’s multiple comparison tests. All statistical analyses were performed using Statistica 12 (StatSoft, Kraków, Poland).

## 5. Conclusions

The conducted chromatographic studies provided considerable new information on the chemical composition of leaves of *P. alba*, *P.* × *candicans*, and *P. nigra*. Eighty-two compounds were identified, thirteen of which were recognized for the first time in the *Populus* genus, and the presence of a number of others was detected for the first time in the individual poplars studied. The observed differences in the content of salicylic glycosides and flavonoids in the leaves of the studied taxa confirm the need for the standardization of herbal substances intended for the pharmaceutical industry.

The compounds that characterize poplar leaves are chlorogenic acid, salicin, and isoquercetin, which were present in high concentrations in all the analyzed leaves. The differences in their chemical composition revealed in this study may have chemotaxonomic significance and allow the distinction of leaves from individual taxa. All analyzed raw materials showed high antioxidant potential in all the tests.

The results of the conducted chromatographic and spectrophotometric studies show that the analyzed poplar leaves can be valuable herbal substances for use in the phytopharmaceutical industry. A particularly valuable plant material, compared to the pharmacopeial herbal substance—*P. nigra* leaves—are the leaves of *P. × candicans*, with the same content of flavonoids and comparable antioxidant activity, but a five-fold higher content of salicylates. Taking into account the observed qualitative and quantitative differences in the chemical composition of the tested poplar leaves, it seems that they can constitute model substances for further in vitro and in vivo studies on their anti-inflammatory activity, explaining the participation of both salicylic and flavonoid compounds in this effect.

## Figures and Tables

**Table 2:** Free (FS) and total salicin content (TSC), total flavonoid (TFC), and total phenolic (TPC) contents of methanol extract from poplar leaves.

*Populus* Species/Hybrid	Salicin Content [mg/g DM] *		TFC		TPC
	FS	TSC	[mg/g DM QE] *	[mg/g DM RE] *	[mg/g DM GAE] *
*P. alba* (A)	8.17 ± 0.02 ^a^	36.16 ± 0.42 ^a^	6.23 ± 0.27 ^a^	12.38 ± 0.54 ^a^	84.13 ± 0.76 ^a^
*P. × candicans* (C)	11.09 ± 0.12 ^b^	21.47 ± 1.55 ^b^	8.69 ± 0.08 ^b^	16.68 ± 1.03 ^b^	81.75 ± 3.07 ^a^
*P. nigra* (N)	1.40 ± 0.01 ^c^	4.42 ± 0.66 ^c^	8.12 ± 0.59 ^b^	17.02 ± 2.41 ^b^	85.29 ± 3.45 ^a^

* Mean ± SD (standard deviation) (*n* = 3); values in individual columns marked with different letters indicate statistically significant differences (*p* < 0.05; Tukey’s RIR test).

**Table 3:** Antioxidant capacity of methanol extracts from poplar leaves.

*Populus* Species/Hybrid	Antioxidant Capacity [mM TEA/g DM] *		
	DPPH	FRAP	ABTS
*P. alba* (A)	0.49 ± 0.03 ^a^	4.82 ± 0.13 ^a^	1.81 ± 0.09 ^a^
*P. × candicans* (C)	0.78 ± 0.01 ^b^	5.19 ± 0.32 ^a^	2.18 ± 0.21 ^b^
*P. nigra* (N)	0.91 ± 0.05 ^c^	6.36 ± 0.22 ^b^	2.51 ± 0.08 ^b^

* Mean ± SD (standard deviation) (*n* = 3); values in individual columns marked with different letters indicate statistically significant differences (*p* < 0.05; Tukey’s RIR test).

## Data Availability

Data are available upon request.

## Supplementary Materials

The following supporting information can be downloaded at: https://www.mdpi.com/article/10.3390/ijms26136189/s1.
