# Supplementary material for: HPLC-DAD-ESI/MS and 2D-TLC Analyses of Secondary Metabolites from Selected Poplar Leaves and an Evaluation of Their Antioxidant Potential
Source: Int J Mol Sci. 2025 Jun 27;26(13):6189. doi: 10.3390/ijms26136189 (PMC12249639; doi:10.3390/ijms26136189)
Supplement: Supplementary file 1 [file ijms-26-06189-s001.zip › ijms-3623135-supplementary.pdf]

Table S1. UV spectra of compounds detected in the methanol extracts of analyzed *Populus* leaves (the numbering used in this table corresponds to the peak numbers separated in the HPLC chromatograms as in Figure 1 and Table 1 of main document).

Peak number	UV spectrum
**1.**	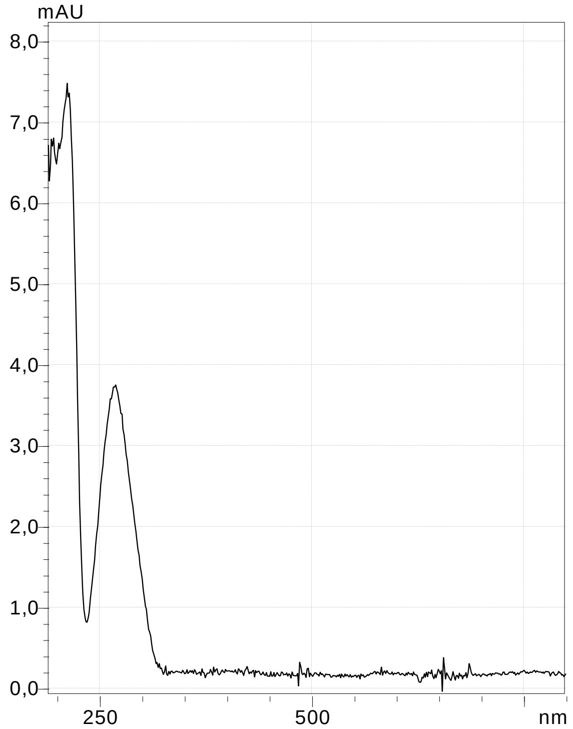
**2.**	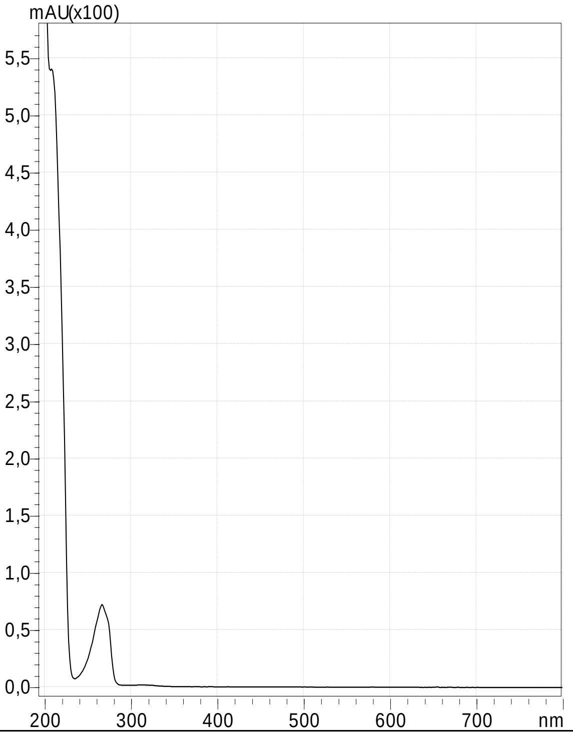

**3.**	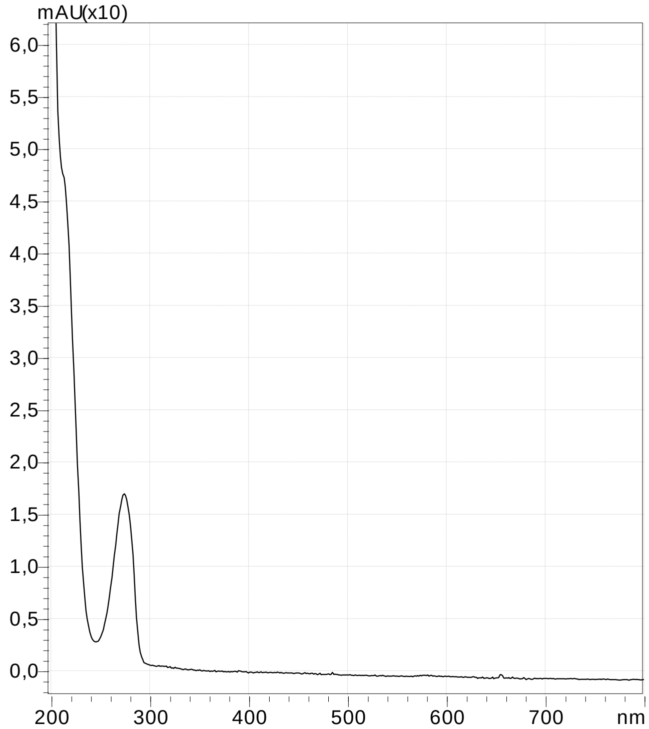 mAU(x10)<br>6,0<br>5,5<br>5,0<br>4,5<br>4,0<br>3,5<br>3,0<br>2,5<br>2,0<br>1,5<br>1,0<br>0,5<br>0,0<br>200<br>300<br>400<br>500<br>600<br>700<br>nm
**4.**	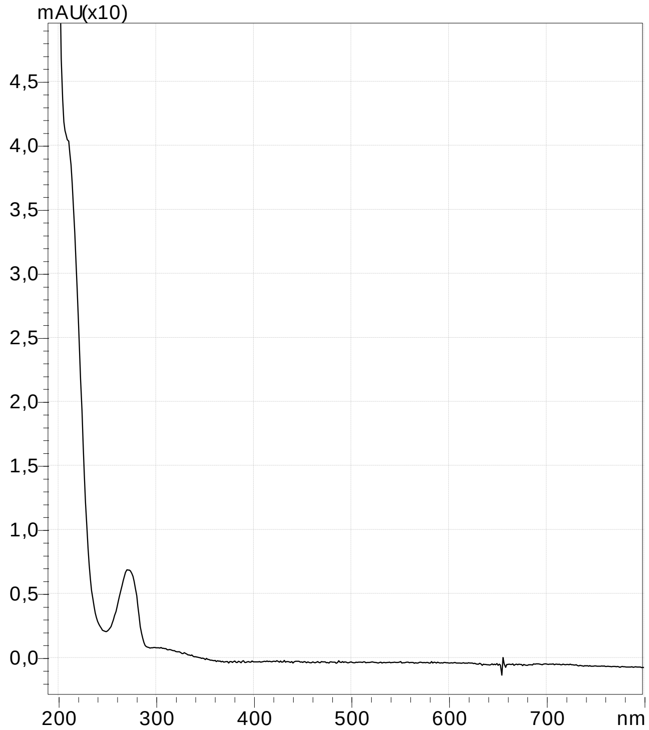 mAU(x10)<br>4,5<br>4,0<br>3,5<br>3,0<br>2,5<br>2,0<br>1,5<br>1,0<br>0,5<br>0,0<br>200<br>300<br>400<br>500<br>600<br>700<br>nm

<table>
<tr><td>5.</td><td>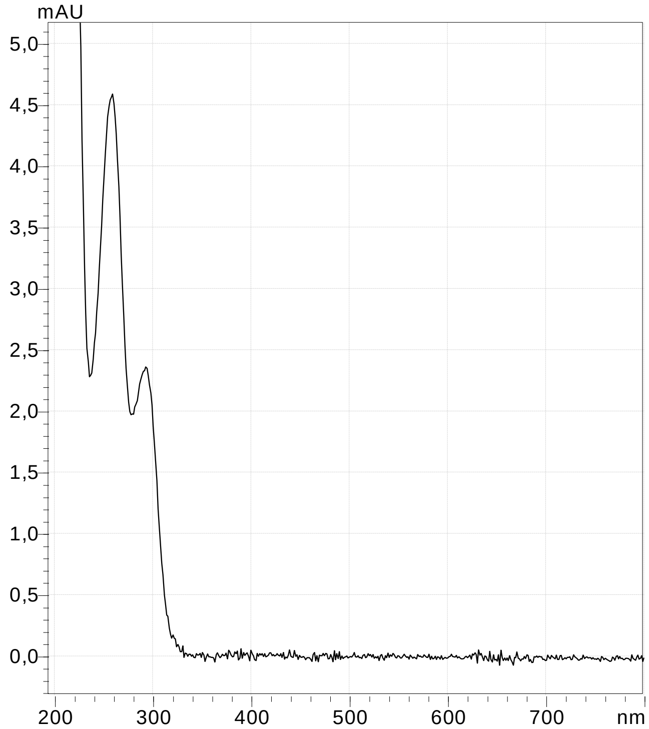
</td></tr>
<tr><td>6.</td><td>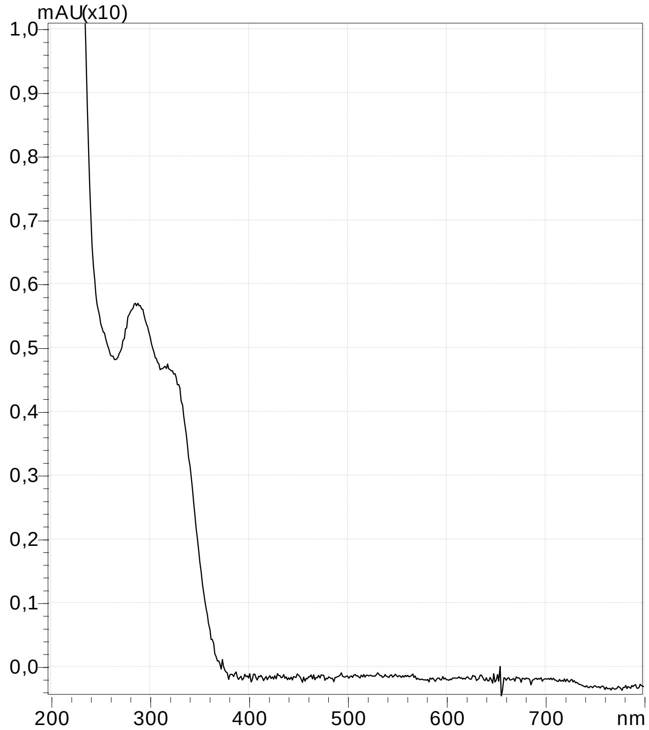
</td></tr>
</table>

**7.**	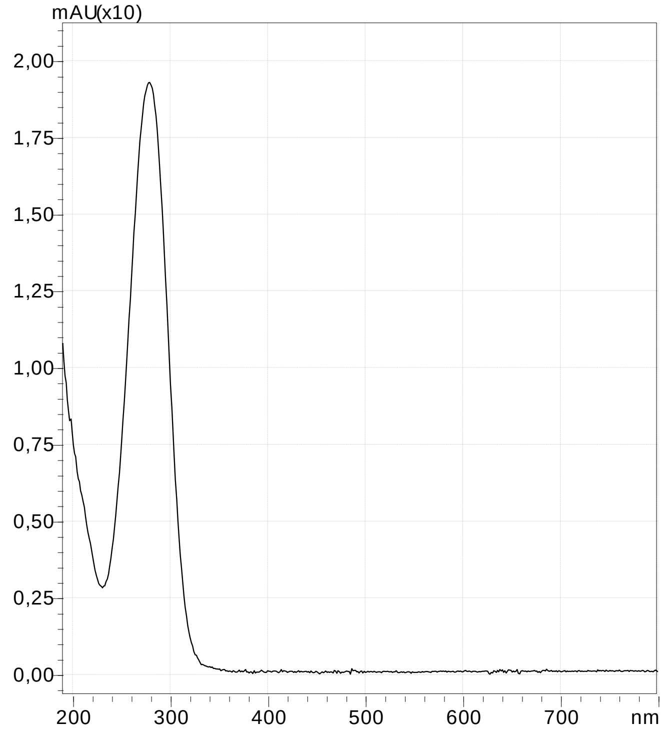mAU(x10)<br>2,00<br>1,75<br>1,50<br>1,25<br>1,00<br>0,75<br>0,50<br>0,25<br>0,00<br>200<br>300<br>400<br>500<br>600<br>700<br>nm
**8.**	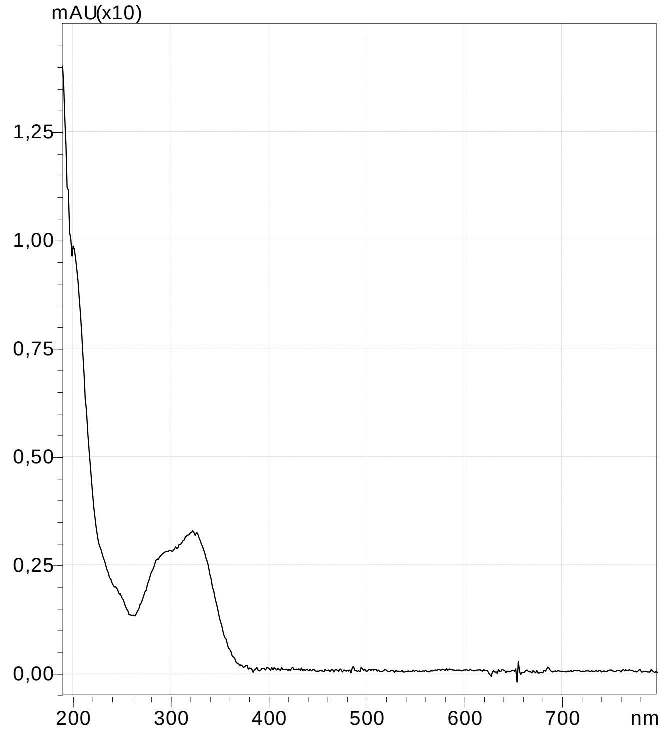mAU(x10)<br>1,25<br>1,00<br>0,75<br>0,50<br>0,25<br>0,00<br>200<br>300<br>400<br>500<br>600<br>700<br>nm

**9.**	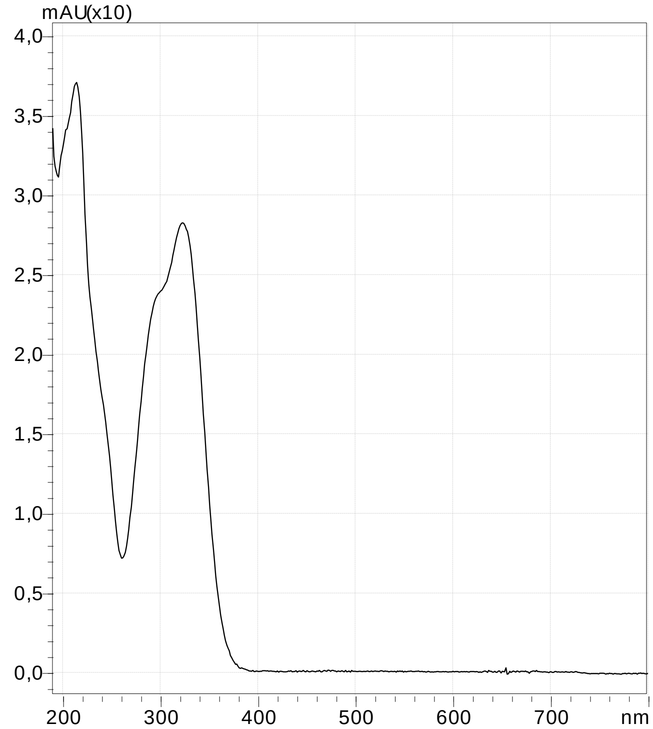mAU(x10)<br>4,0<br>3,5<br>3,0<br>2,5<br>2,0<br>1,5<br>1,0<br>0,5<br>0,0<br>200<br>300<br>400<br>500<br>600<br>700<br>nm
**10.**	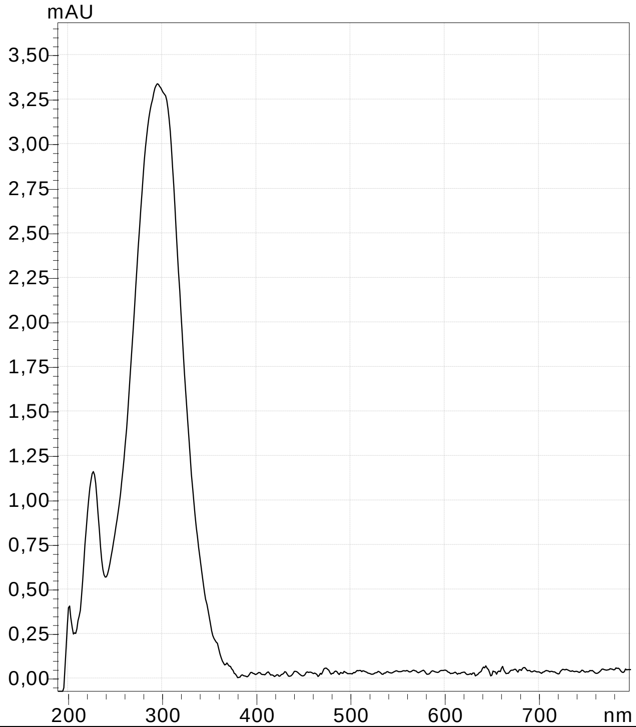mAU<br>3,50<br>3,25<br>3,00<br>2,75<br>2,50<br>2,25<br>2,00<br>1,75<br>1,50<br>1,25<br>1,00<br>0,75<br>0,50<br>0,25<br>0,00<br>200<br>300<br>400<br>500<br>600<br>700<br>nm

<table>
<tr>
<td>11.</td>
<td>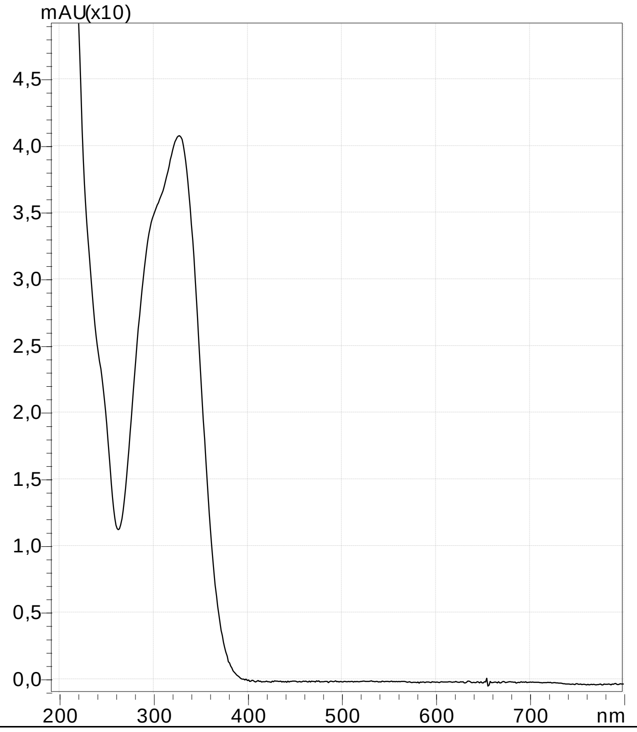
</td>
</tr>
<tr>
<td>12.</td>
<td>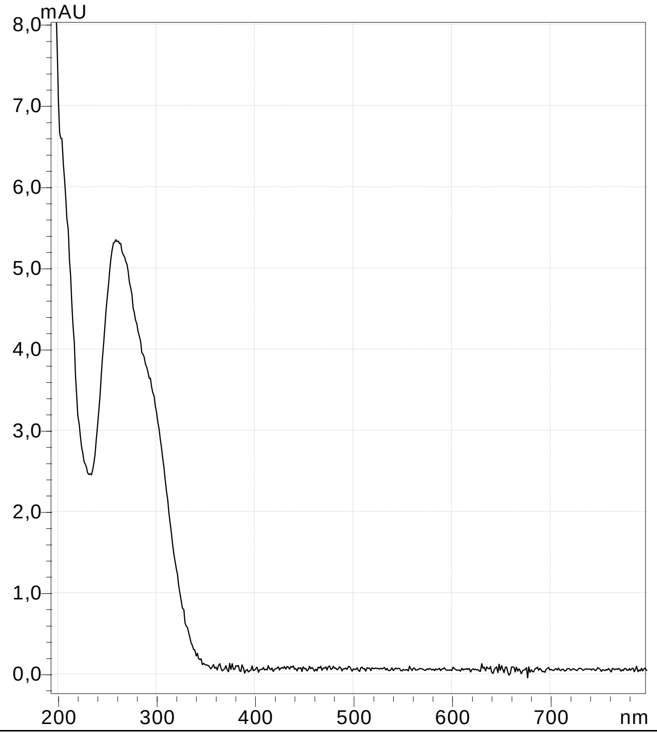
</td>
</tr>
</table>

**13.**	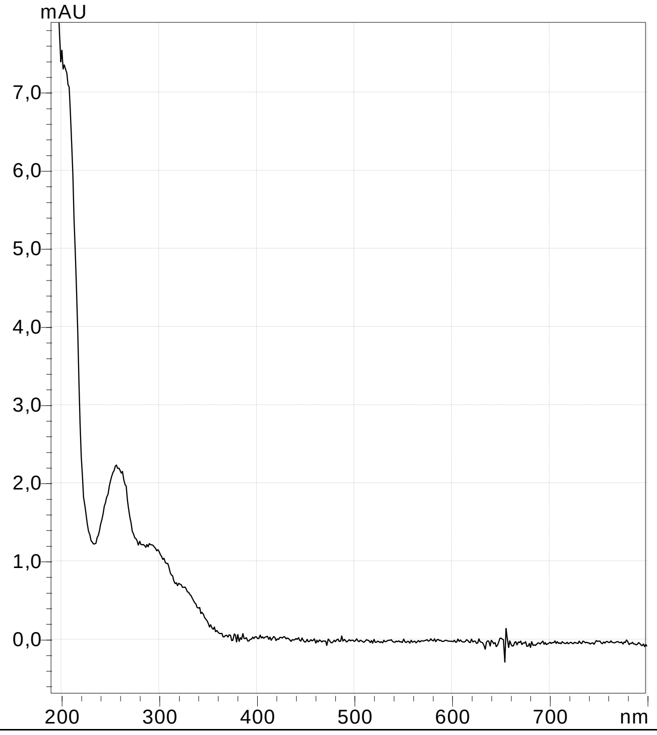mAU<br>7,0<br>6,0<br>5,0<br>4,0<br>3,0<br>2,0<br>1,0<br>0,0<br>200<br>300<br>400<br>500<br>600<br>700<br>nm
**14.**	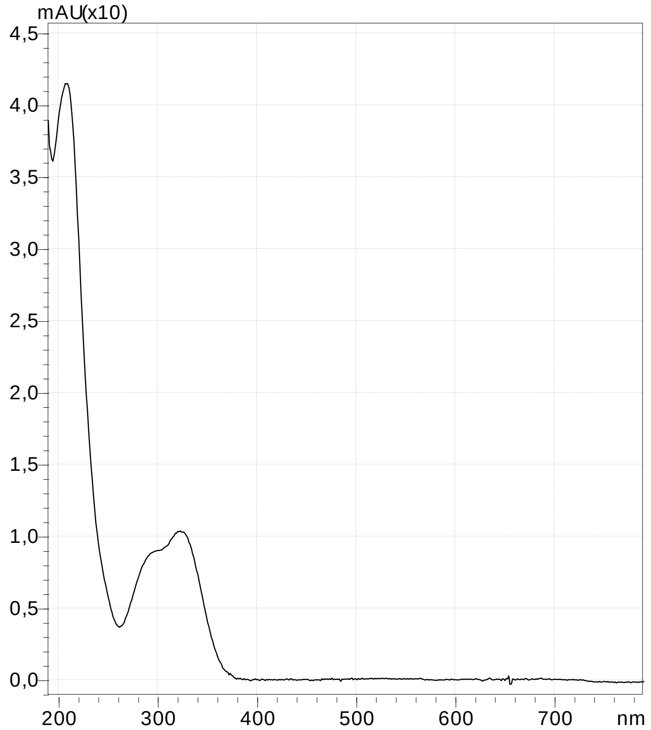mAU(x10)<br>4,5<br>4,0<br>3,5<br>3,0<br>2,5<br>2,0<br>1,5<br>1,0<br>0,5<br>0,0<br>200<br>300<br>400<br>500<br>600<br>700<br>nm

**15.**	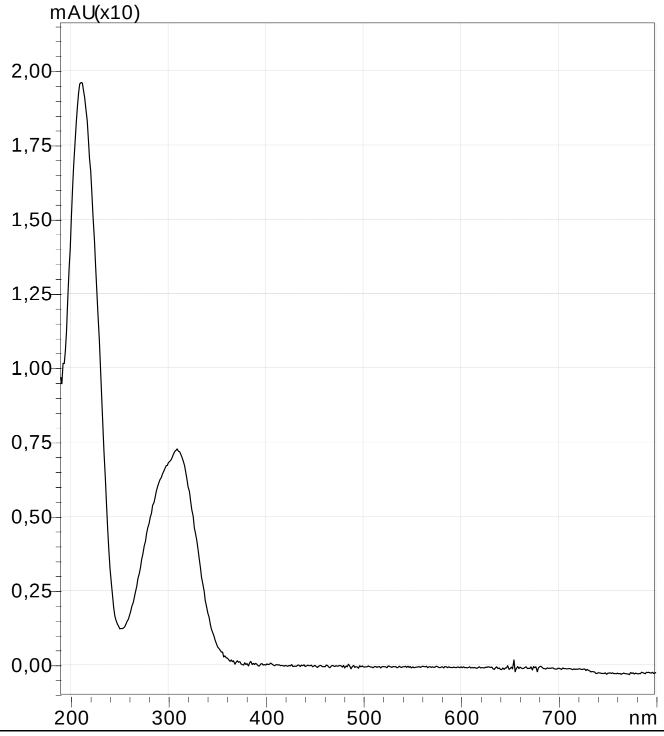mAU(x10)<br>2,00<br>1,75<br>1,50<br>1,25<br>1,00<br>0,75<br>0,50<br>0,25<br>0,00<br>200<br>300<br>400<br>500<br>600<br>700<br>nm
**16.**	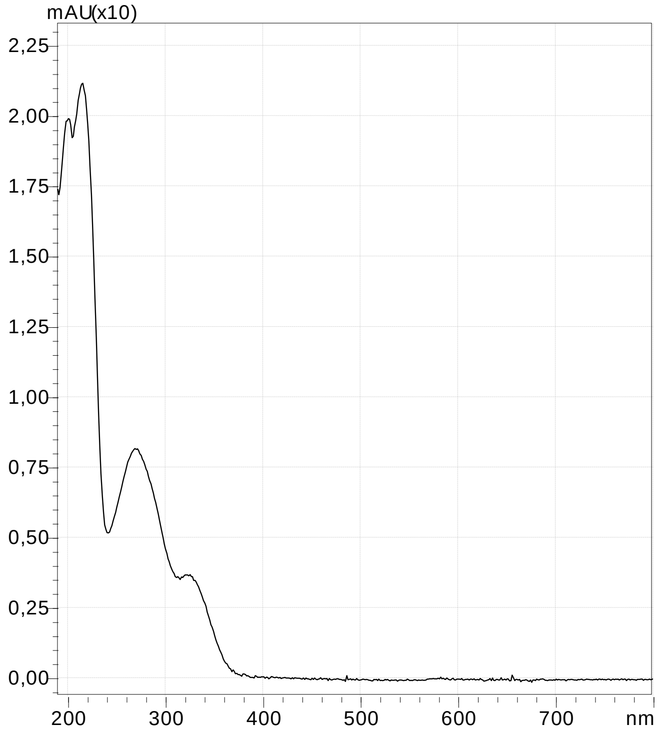mAU(x10)<br>2,25<br>2,00<br>1,75<br>1,50<br>1,25<br>1,00<br>0,75<br>0,50<br>0,25<br>0,00<br>200<br>300<br>400<br>500<br>600<br>700<br>nm

**17.**	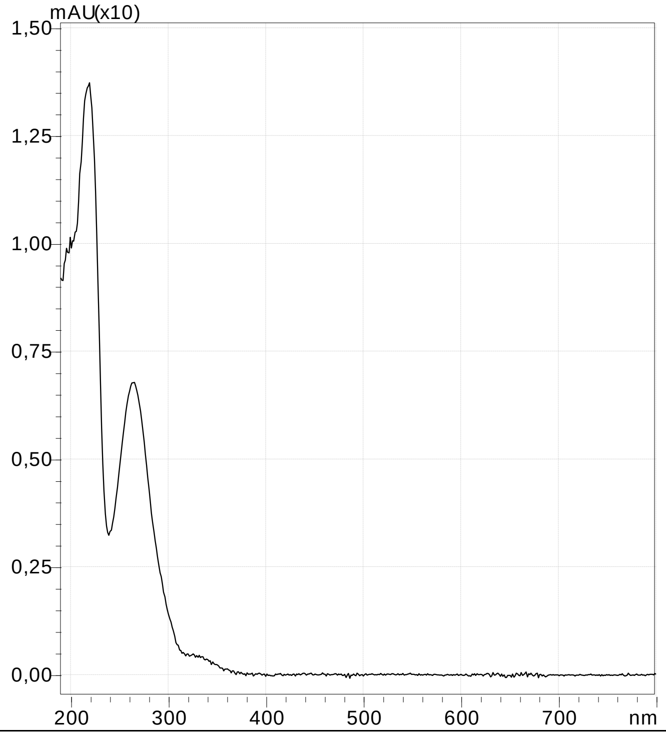mAU(x10)<br>1,50<br>1,25<br>1,00<br>0,75<br>0,50<br>0,25<br>0,00<br>200 300 400 500 600 700 nm
**18.**	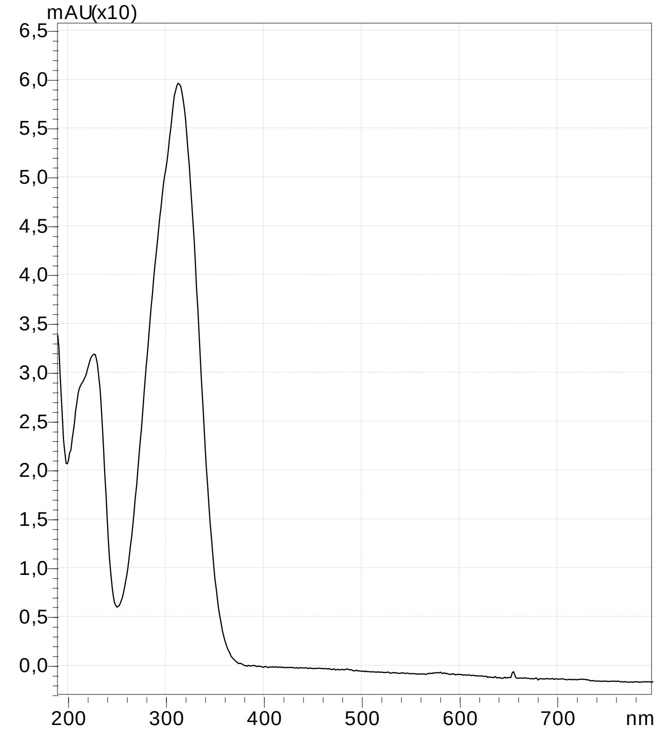mAU(x10)<br>6,5<br>6,0<br>5,5<br>5,0<br>4,5<br>4,0<br>3,5<br>3,0<br>2,5<br>2,0<br>1,5<br>1,0<br>0,5<br>0,0<br>200 300 400 500 600 700 nm

**19.**	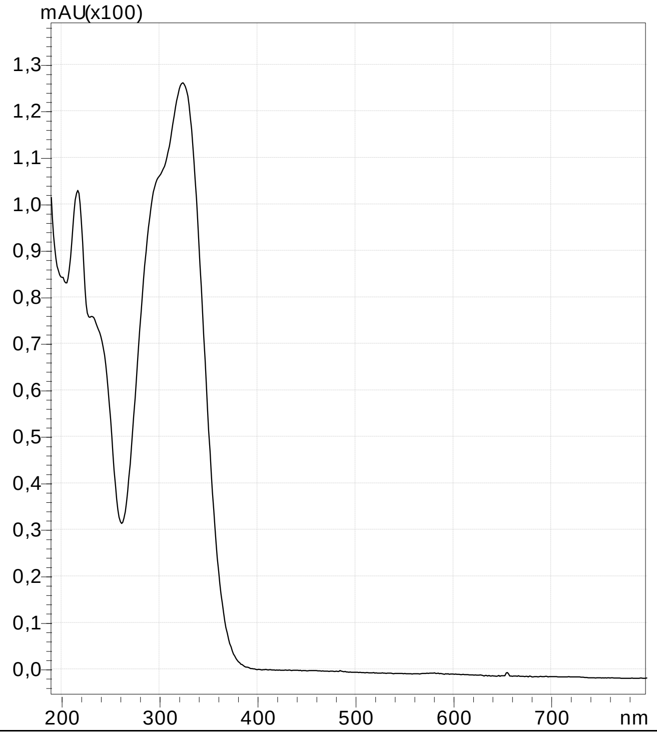mAU(x100)
1,3
1,2
1,1
1,0
0,9
0,8
0,7
0,6
0,5
0,4
0,3
0,2
0,1
0,0
200
300
400
500
600
700
nm
**20.**	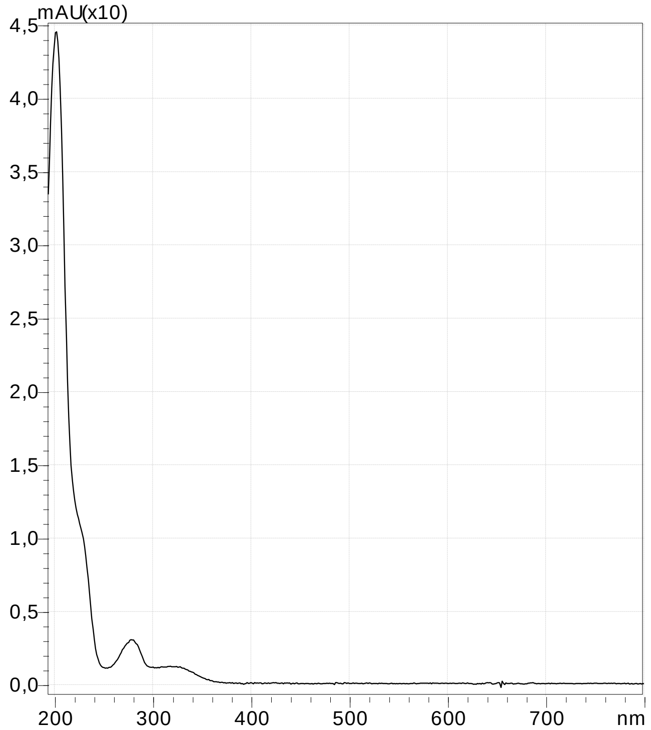mAU(x10)
4,5
4,0
3,5
3,0
2,5
2,0
1,5
1,0
0,5
0,0
200
300
400
500
600
700
nm

**21.**	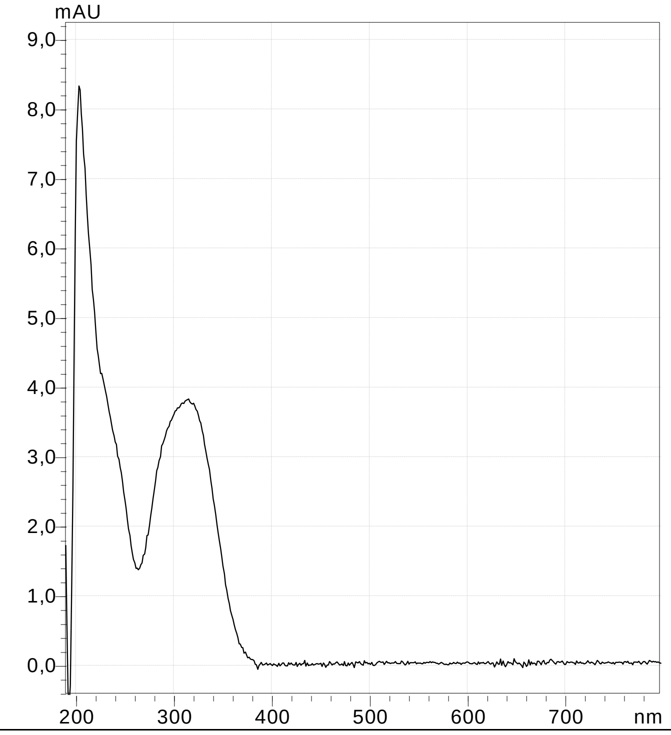
**22.**	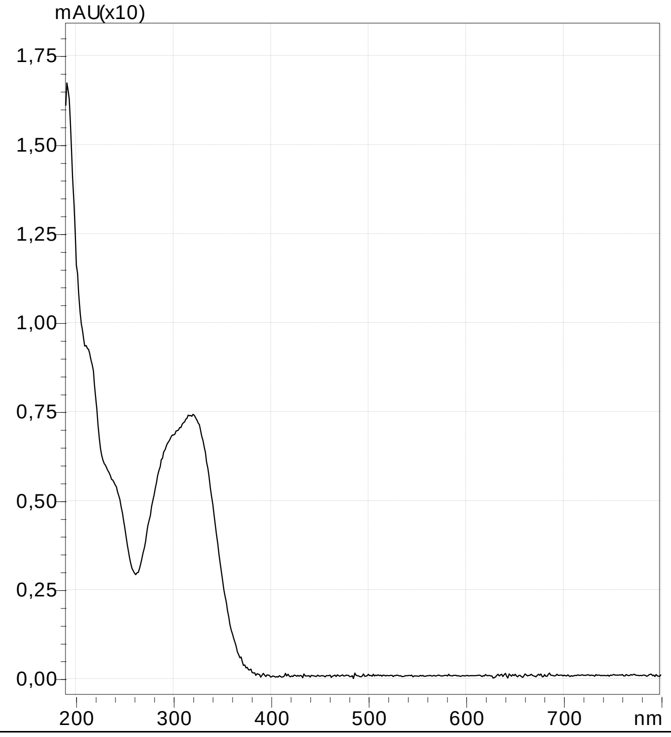

mAU
9,0
8,0
7,0
6,0
5,0
4,0
3,0
2,0
1,0
0,0
200
300
400
500
600
700
nm

mAU(x10)
1,75
1,50
1,25
1,00
0,75
0,50
0,25
0,00
200
300
400
500
600
700
nm

**23.**	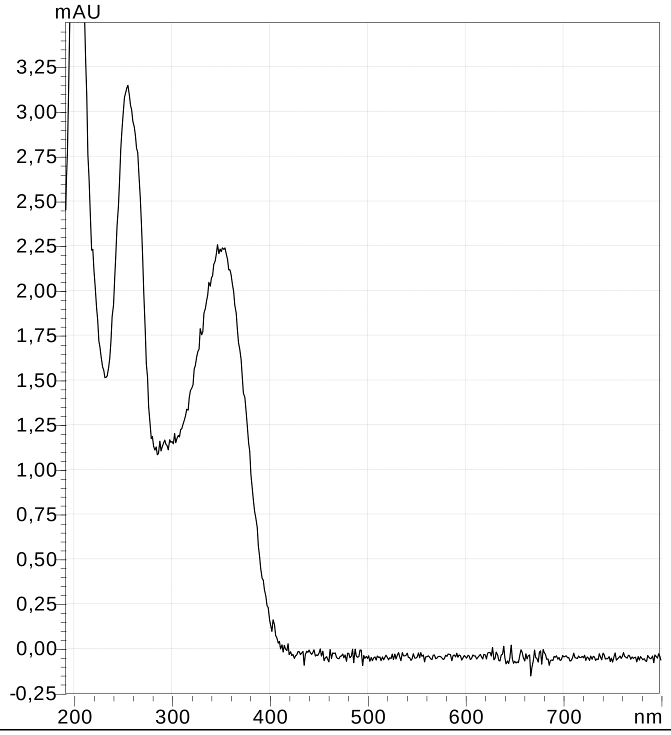mAU<br>3,25<br>3,00<br>2,75<br>2,50<br>2,25<br>2,00<br>1,75<br>1,50<br>1,25<br>1,00<br>0,75<br>0,50<br>0,25<br>0,00<br>-0,25<br>200<br>300<br>400<br>500<br>600<br>700<br>nm
**24.**	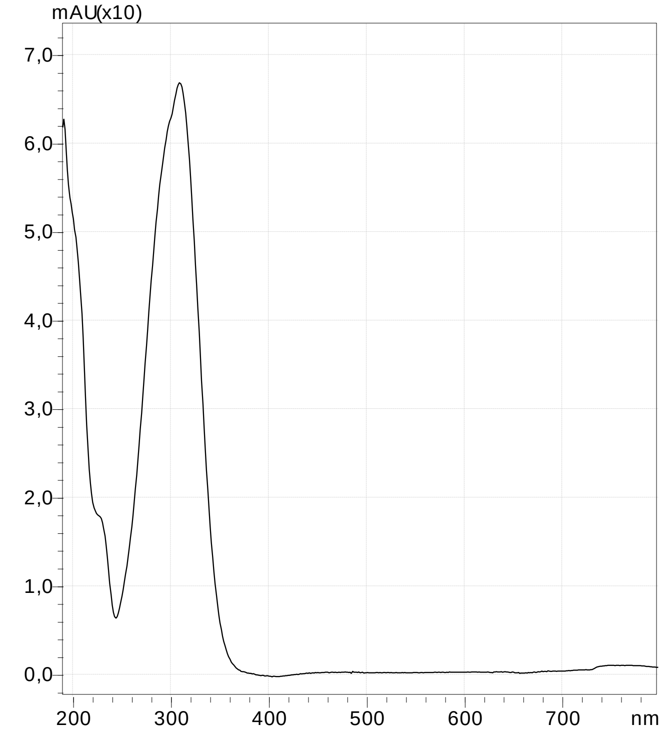mAU(x10)<br>7,0<br>6,0<br>5,0<br>4,0<br>3,0<br>2,0<br>1,0<br>0,0<br>200<br>300<br>400<br>500<br>600<br>700<br>nm

**25.**	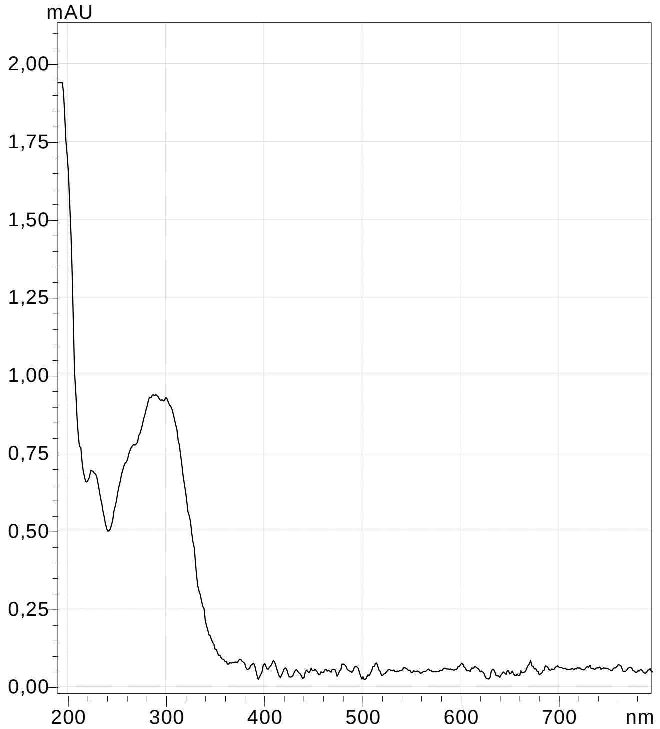 mAU 2,00 1,75 1,50 1,25 1,00 0,75 0,50 0,25 0,00 200 300 400 500 600 700 nm
**26.**	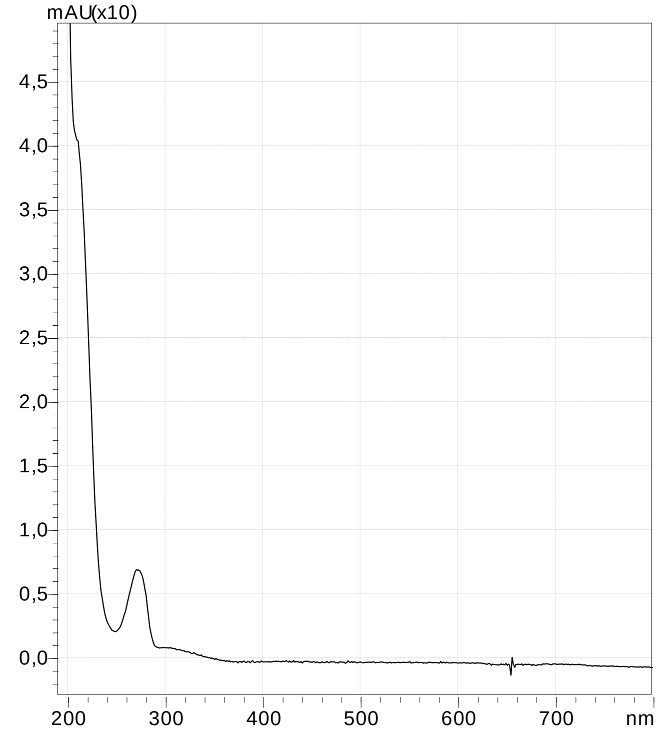 mAU(x10) 4,5 4,0 3,5 3,0 2,5 2,0 1,5 1,0 0,5 0,0 200 300 400 500 600 700 nm

**27.**	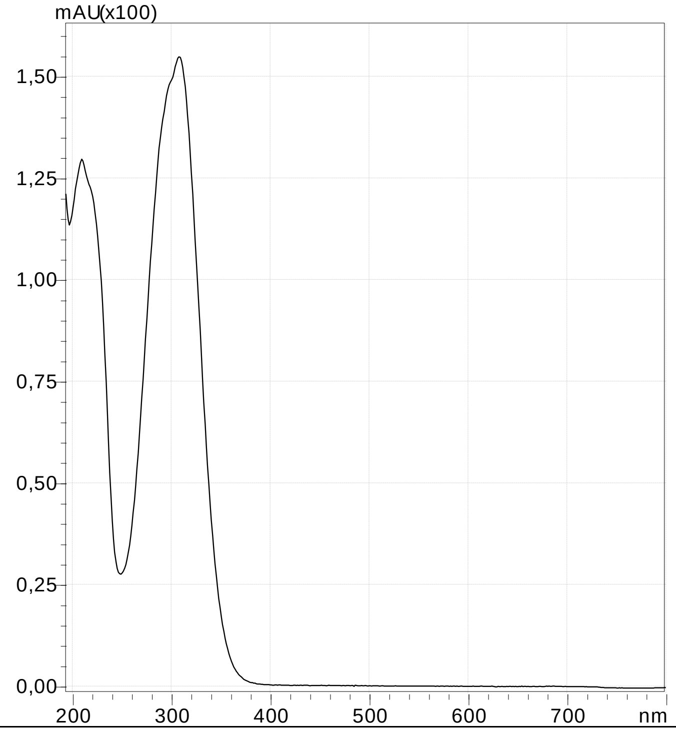mAU(x100)<br>1,50<br>1,25<br>1,00<br>0,75<br>0,50<br>0,25<br>0,00<br>200<br>300<br>400<br>500<br>600<br>700<br>nm
**28.**	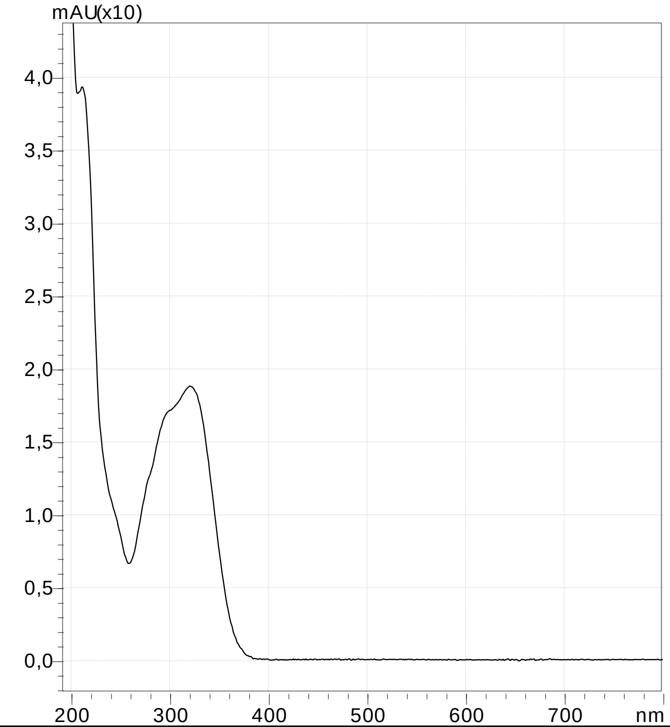mAU(x10)<br>4,0<br>3,5<br>3,0<br>2,5<br>2,0<br>1,5<br>1,0<br>0,5<br>0,0<br>200<br>300<br>400<br>500<br>600<br>700<br>nm

**29.**	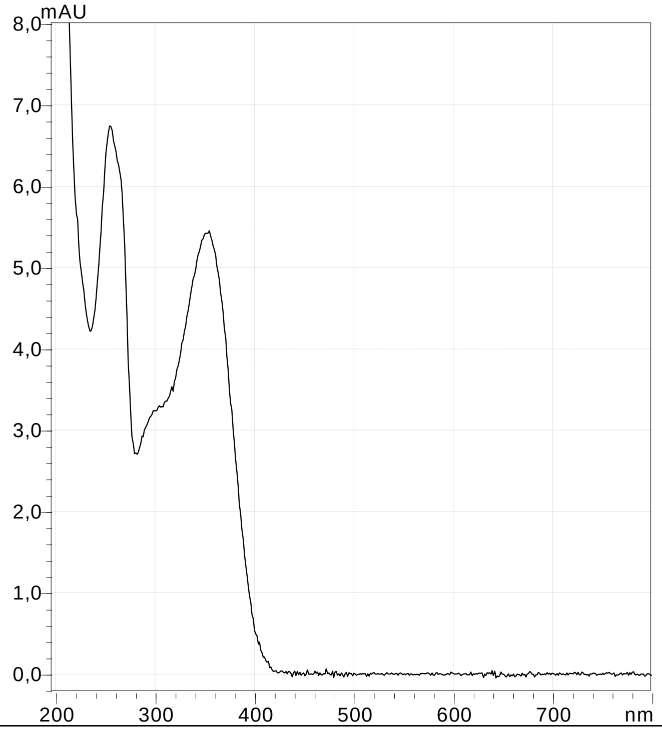mAU<br>8,0<br>7,0<br>6,0<br>5,0<br>4,0<br>3,0<br>2,0<br>1,0<br>0,0<br>200<br>300<br>400<br>500<br>600<br>700<br>nm
**30.**	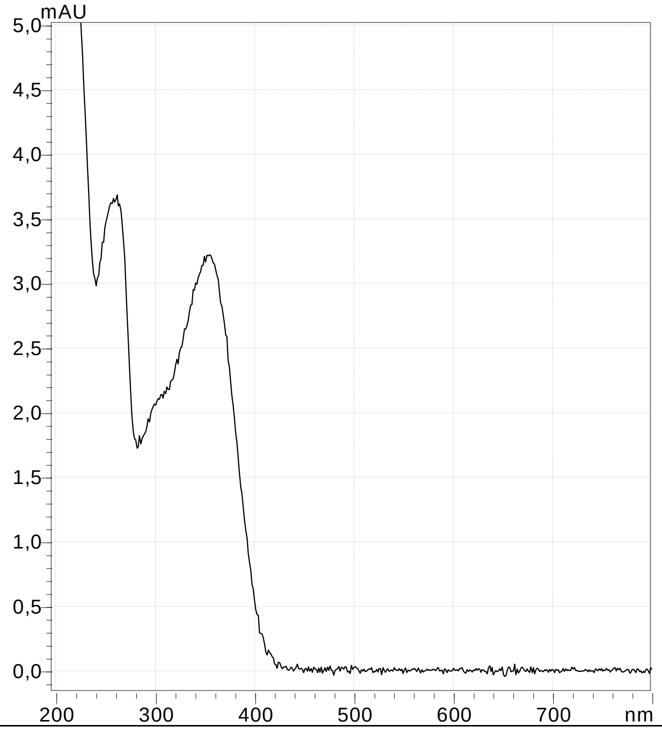mAU<br>5,0<br>4,5<br>4,0<br>3,5<br>3,0<br>2,5<br>2,0<br>1,5<br>1,0<br>0,5<br>0,0<br>200<br>300<br>400<br>500<br>600<br>700<br>nm

**31.**	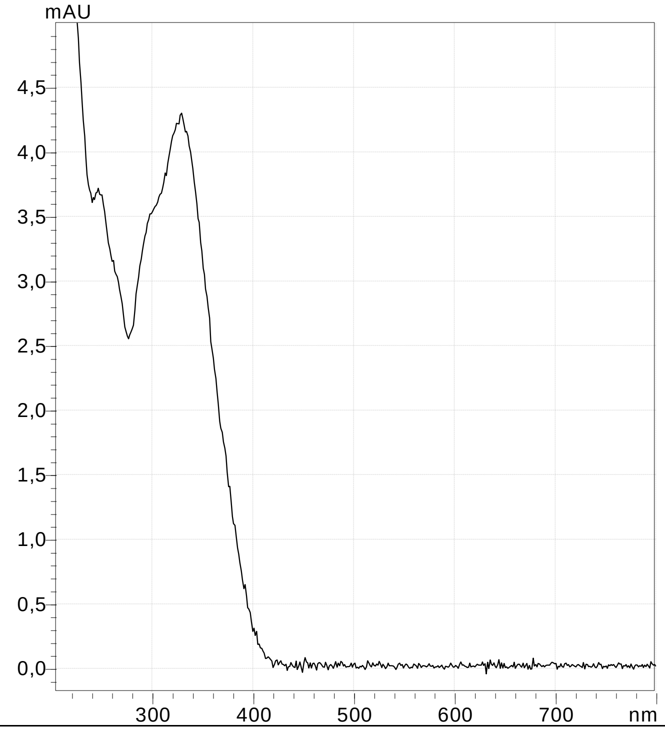 mAU<br>4,5<br>4,0<br>3,5<br>3,0<br>2,5<br>2,0<br>1,5<br>1,0<br>0,5<br>0,0<br>300<br>400<br>500<br>600<br>700<br>nm
**32.**	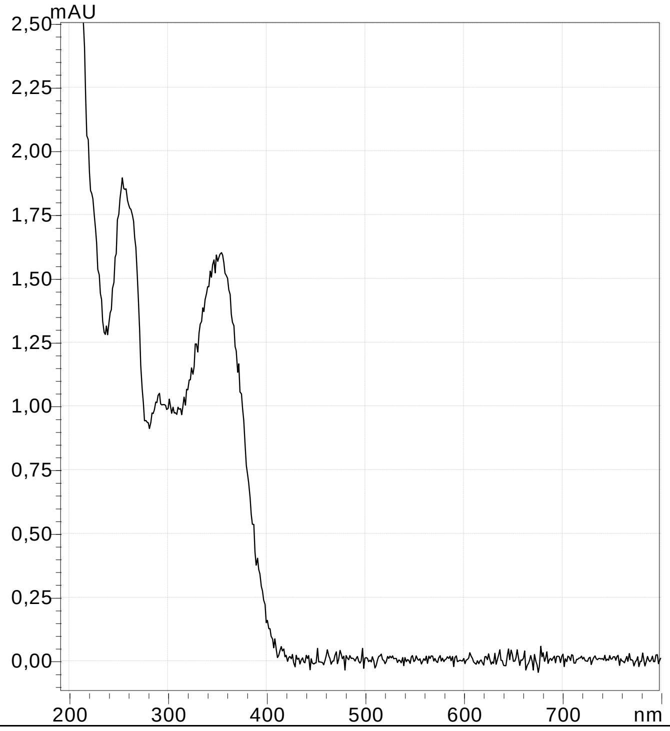 mAU<br>2,50<br>2,25<br>2,00<br>1,75<br>1,50<br>1,25<br>1,00<br>0,75<br>0,50<br>0,25<br>0,00<br>200<br>300<br>400<br>500<br>600<br>700<br>nm

**33.**	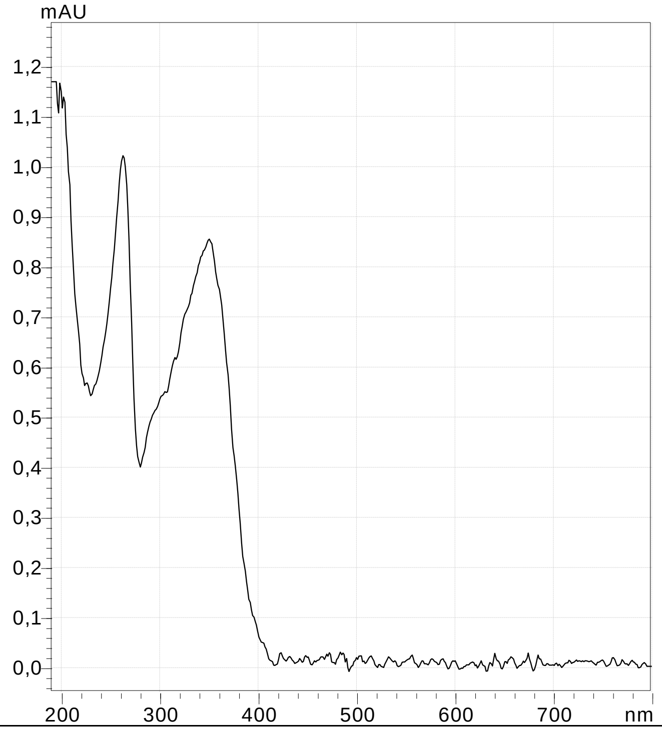mAU<br>1,2<br>1,1<br>1,0<br>0,9<br>0,8<br>0,7<br>0,6<br>0,5<br>0,4<br>0,3<br>0,2<br>0,1<br>0,0<br>200<br>300<br>400<br>500<br>600<br>700<br>nm
**34.**	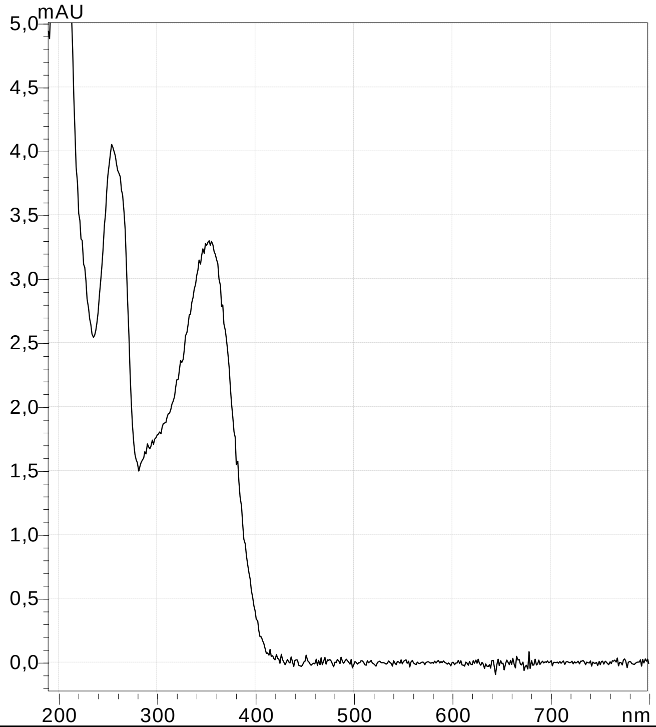mAU<br>5,0<br>4,5<br>4,0<br>3,5<br>3,0<br>2,5<br>2,0<br>1,5<br>1,0<br>0,5<br>0,0<br>200<br>300<br>400<br>500<br>600<br>700<br>nm

**35**	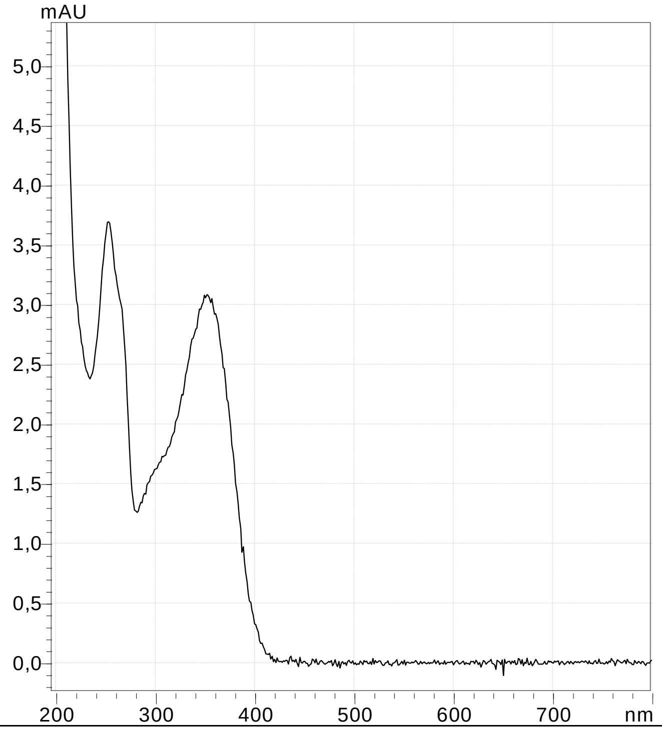mAU<br>5,0<br>4,5<br>4,0<br>3,5<br>3,0<br>2,5<br>2,0<br>1,5<br>1,0<br>0,5<br>0,0<br>200<br>300<br>400<br>500<br>600<br>700<br>nm
**36.**	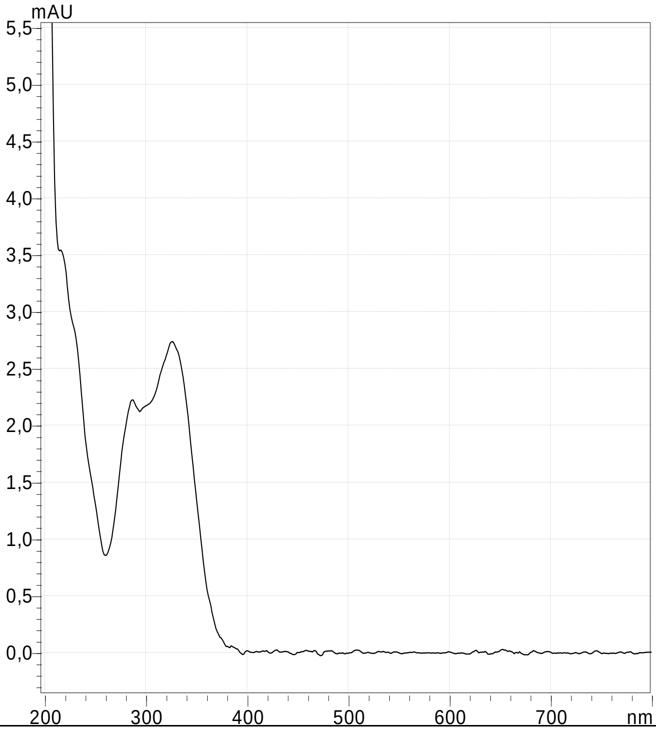mAU<br>5,5<br>5,0<br>4,5<br>4,0<br>3,5<br>3,0<br>2,5<br>2,0<br>1,5<br>1,0<br>0,5<br>0,0<br>200<br>300<br>400<br>500<br>600<br>700<br>nm

**37.**	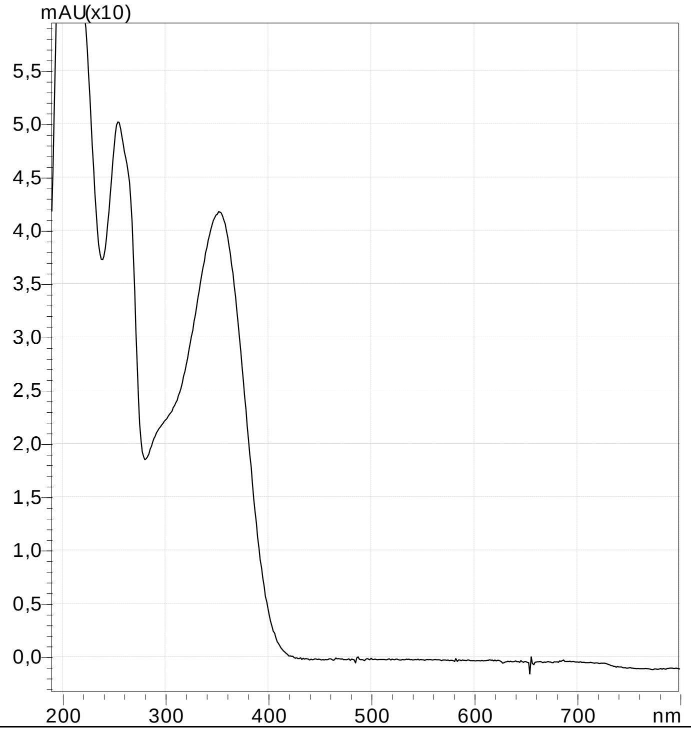mAU(x10)<br>5,5<br>5,0<br>4,5<br>4,0<br>3,5<br>3,0<br>2,5<br>2,0<br>1,5<br>1,0<br>0,5<br>0,0<br>200<br>300<br>400<br>500<br>600<br>700<br>nm
**38.**	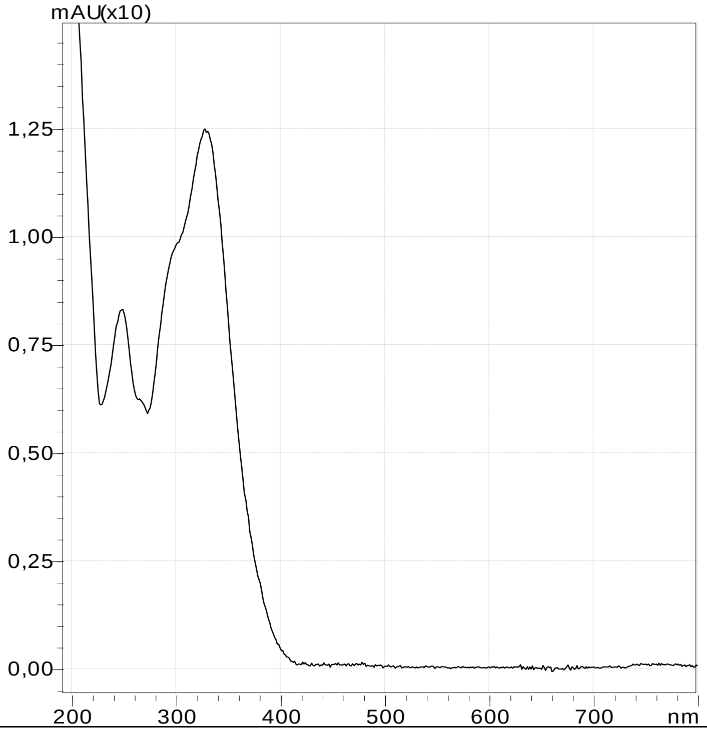mAU(x10)<br>1,25<br>1,00<br>0,75<br>0,50<br>0,25<br>0,00<br>200<br>300<br>400<br>500<br>600<br>700<br>nm

**39.**	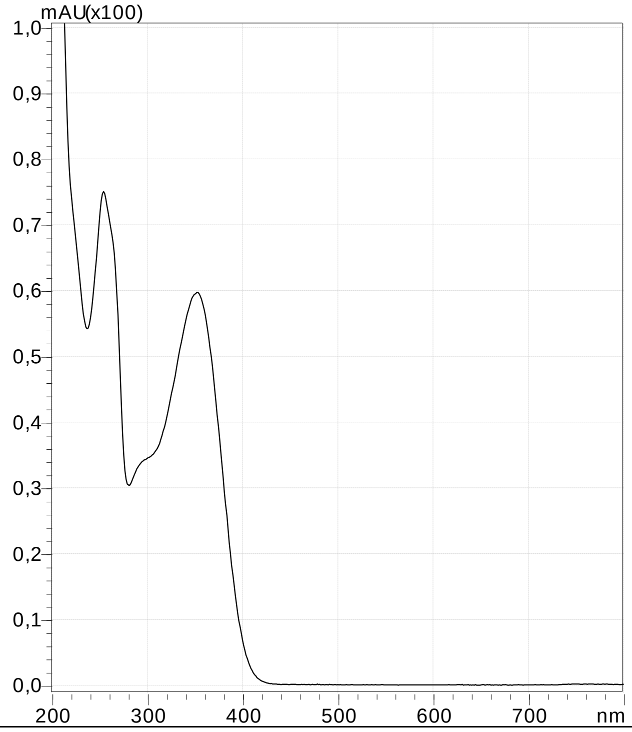mAU(x100)
1,0
0,9
0,8
0,7
0,6
0,5
0,4
0,3
0,2
0,1
0,0
200
300
400
500
600
700
nm
**40.**	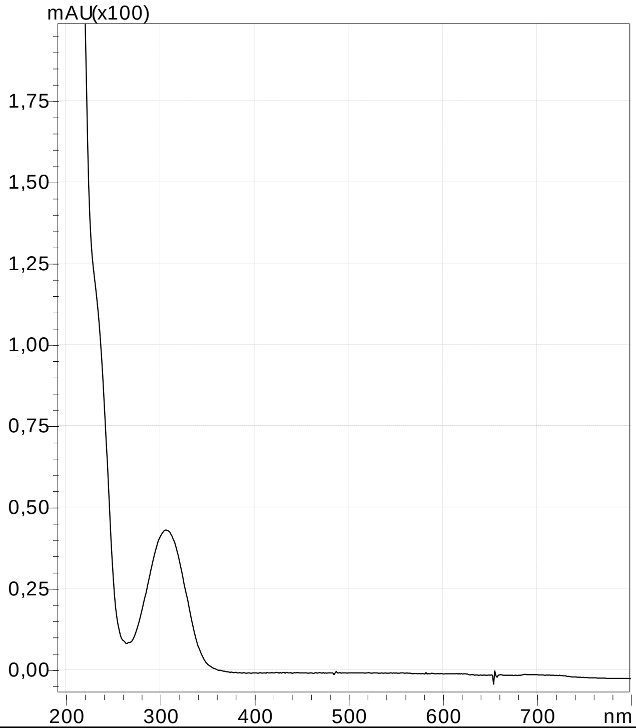mAU(x100)
1,75
1,50
1,25
1,00
0,75
0,50
0,25
0,00
200
300
400
500
600
700
nm

**41.**	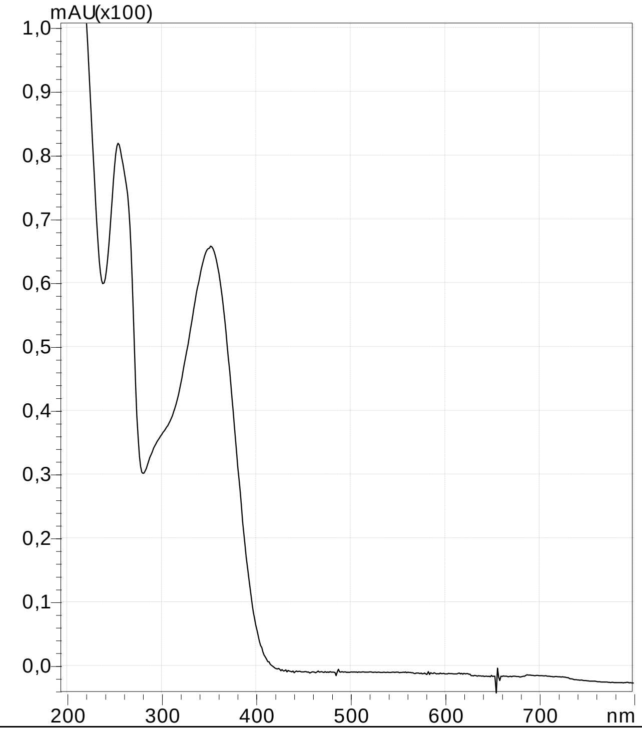mAU(x100)<br>1,0<br>0,9<br>0,8<br>0,7<br>0,6<br>0,5<br>0,4<br>0,3<br>0,2<br>0,1<br>0,0<br>200<br>300<br>400<br>500<br>600<br>700<br>nm
**42.**	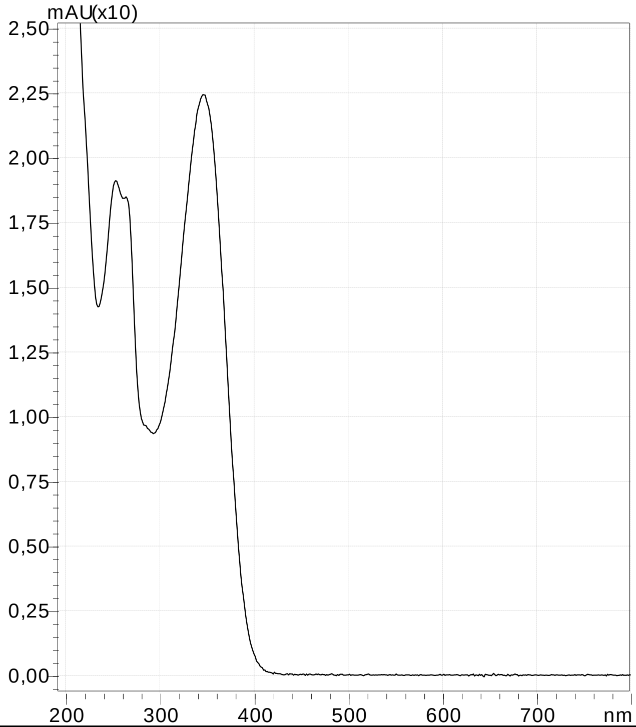mAU(x10)<br>2,50<br>2,25<br>2,00<br>1,75<br>1,50<br>1,25<br>1,00<br>0,75<br>0,50<br>0,25<br>0,00<br>200<br>300<br>400<br>500<br>600<br>700<br>nm

**43.**	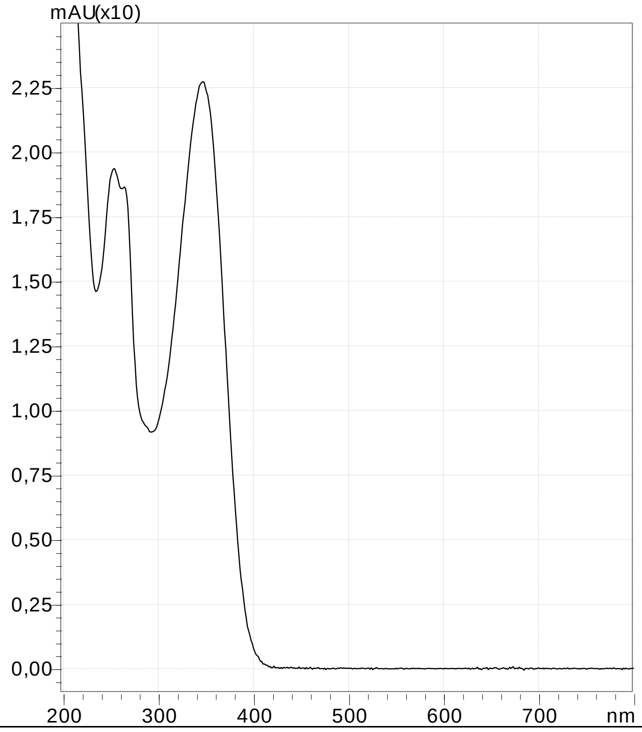mAU(x10)<br>2,25<br>2,00<br>1,75<br>1,50<br>1,25<br>1,00<br>0,75<br>0,50<br>0,25<br>0,00<br>200 300 400 500 600 700 nm
**44.**	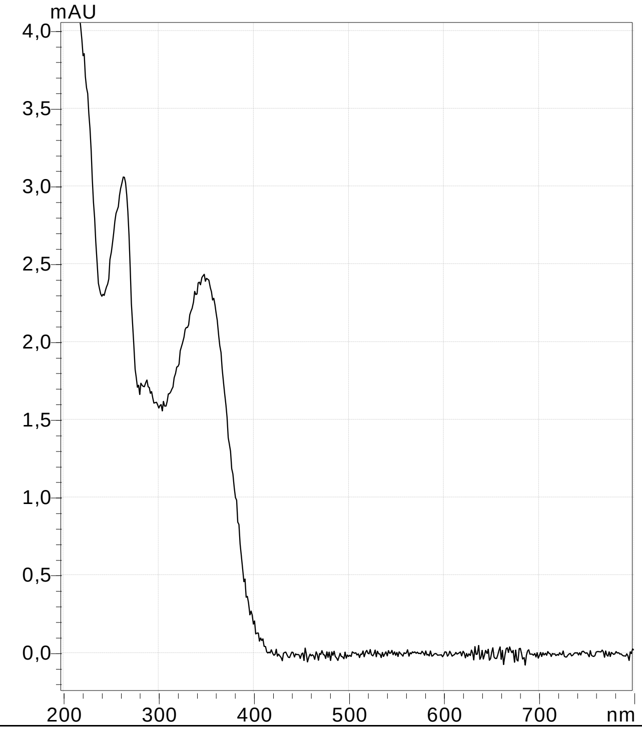mAU<br>4,0<br>3,5<br>3,0<br>2,5<br>2,0<br>1,5<br>1,0<br>0,5<br>0,0<br>200 300 400 500 600 700 nm

45.

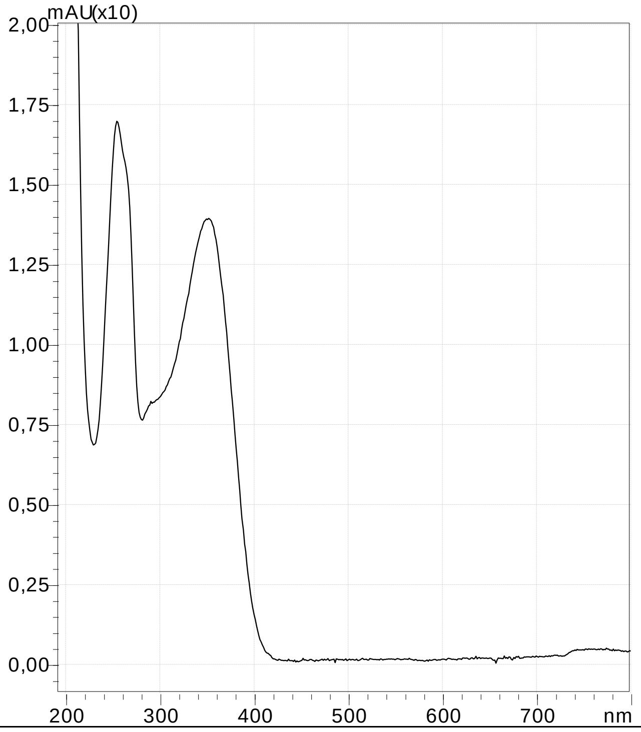
mAU(x10)
2,00
1,75
1,50
1,25
1,00
0,75
0,50
0,25
0,00
200
300
400
500
600
700
nm

46.

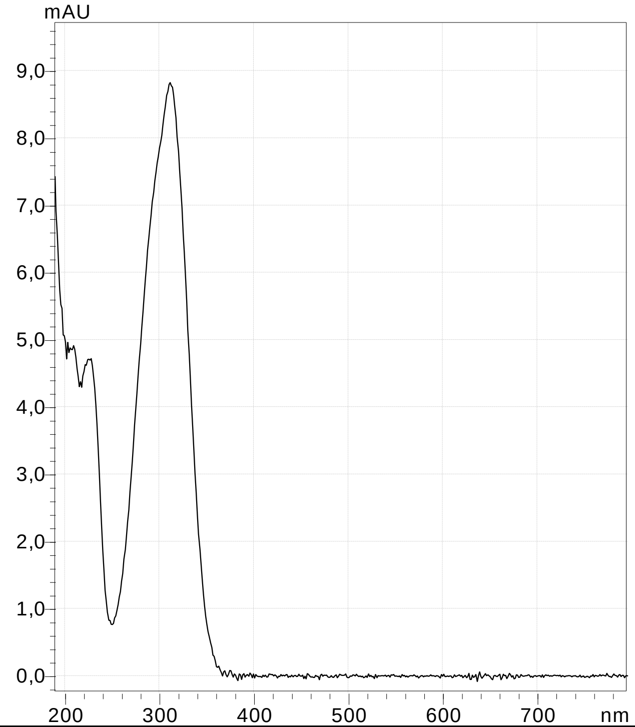
mAU
9,0
8,0
7,0
6,0
5,0
4,0
3,0
2,0
1,0
0,0
200
300
400
500
600
700
nm

**47.**	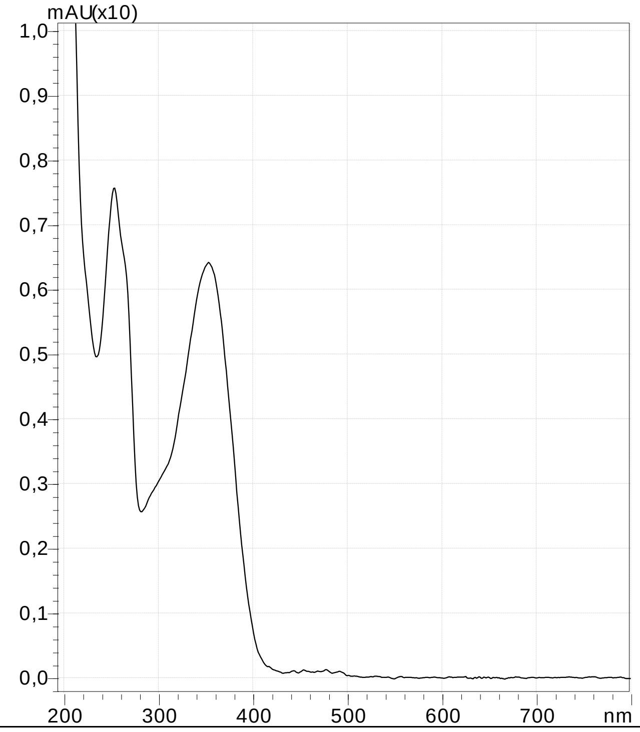mAU(x10)<br>1,0<br>0,9<br>0,8<br>0,7<br>0,6<br>0,5<br>0,4<br>0,3<br>0,2<br>0,1<br>0,0<br>200<br>300<br>400<br>500<br>600<br>700<br>nm
**48.**	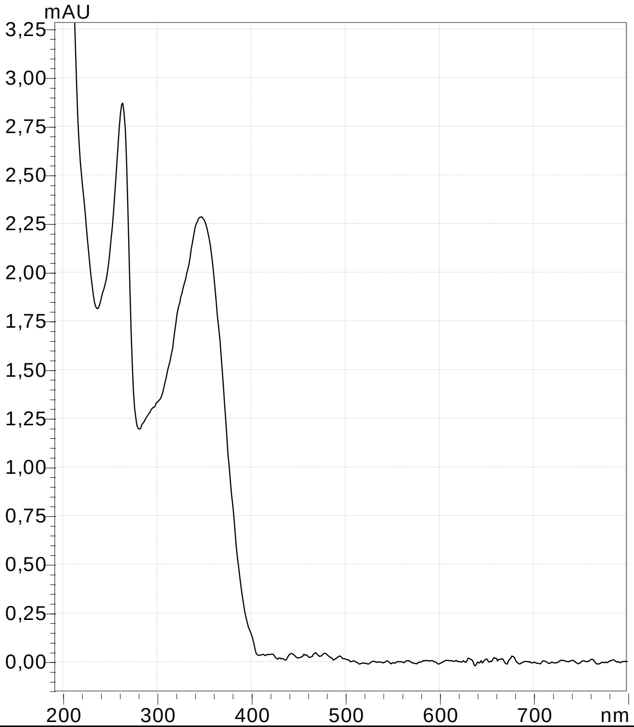mAU<br>3,25<br>3,00<br>2,75<br>2,50<br>2,25<br>2,00<br>1,75<br>1,50<br>1,25<br>1,00<br>0,75<br>0,50<br>0,25<br>0,00<br>200<br>300<br>400<br>500<br>600<br>700<br>nm

**49.**	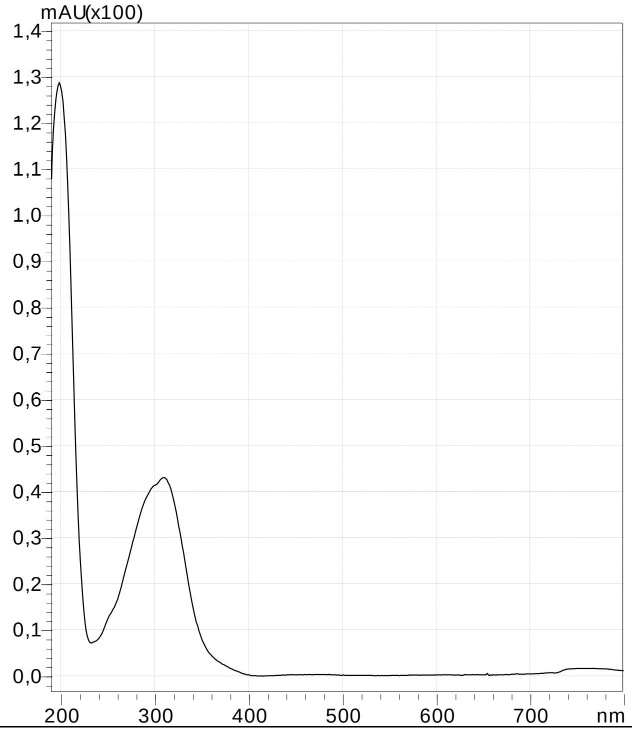
**50.**	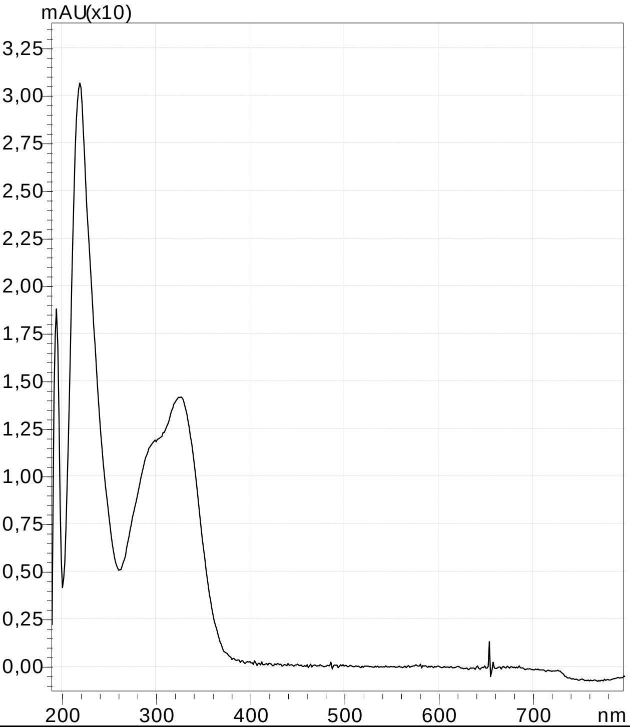

mAU(x100)
1,4
1,3
1,2
1,1
1,0
0,9
0,8
0,7
0,6
0,5
0,4
0,3
0,2
0,1
0,0
200
300
400
500
600
700
nm

mAU(x10)
3,25
3,00
2,75
2,50
2,25
2,00
1,75
1,50
1,25
1,00
0,75
0,50
0,25
0,00
200
300
400
500
600
700
nm

51.

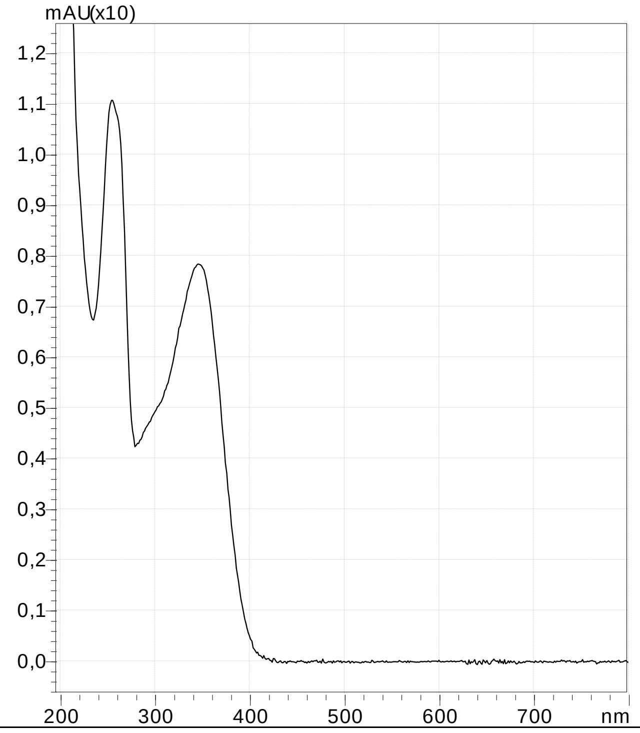
mAU(x10)
1,2
1,1
1,0
0,9
0,8
0,7
0,6
0,5
0,4
0,3
0,2
0,1
0,0
200
300
400
500
600
700
nm

52.

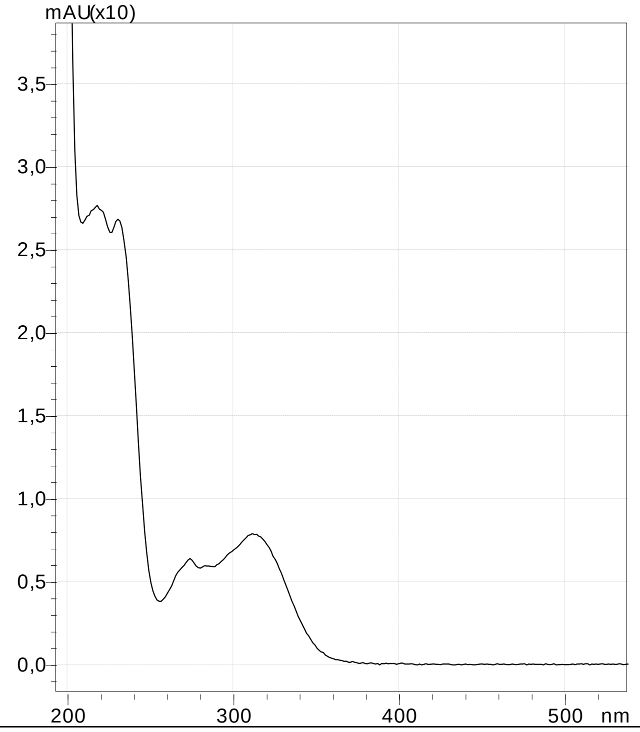
mAU(x10)
3,5
3,0
2,5
2,0
1,5
1,0
0,5
0,0
200
300
400
500
nm

53.

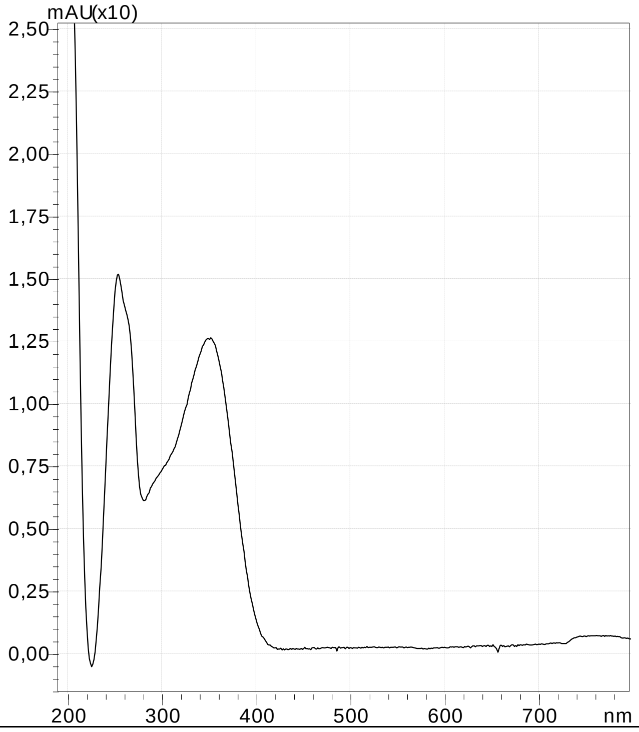
mAU(x10)
2,50
2,25
2,00
1,75
1,50
1,25
1,00
0,75
0,50
0,25
0,00
200
300
400
500
600
700
nm

54.

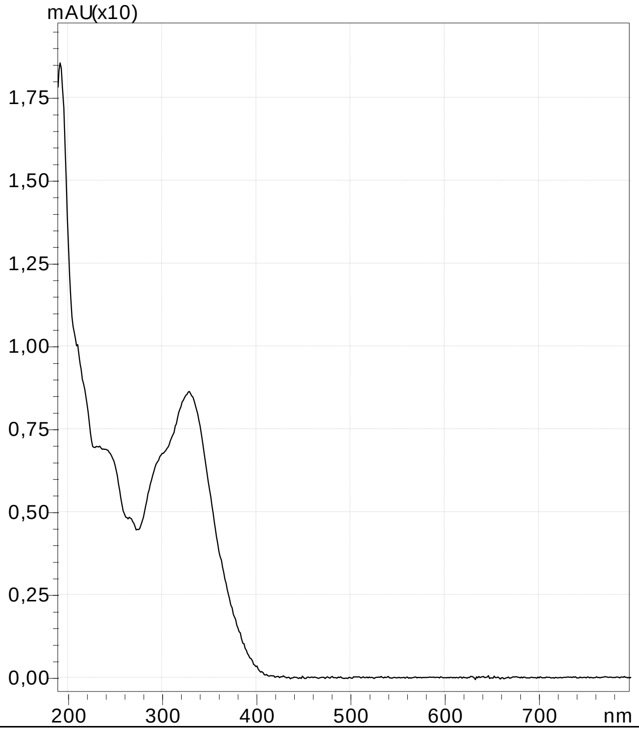
mAU(x10)
1,75
1,50
1,25
1,00
0,75
0,50
0,25
0,00
200
300
400
500
600
700
nm

55.

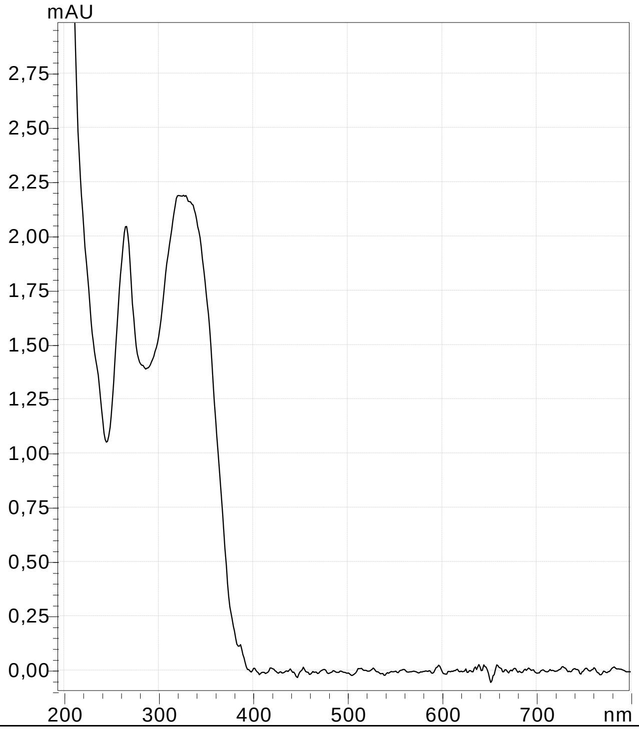
mAU
2,75
2,50
2,25
2,00
1,75
1,50
1,25
1,00
0,75
0,50
0,25
0,00
200
300
400
500
600
700
nm

56.

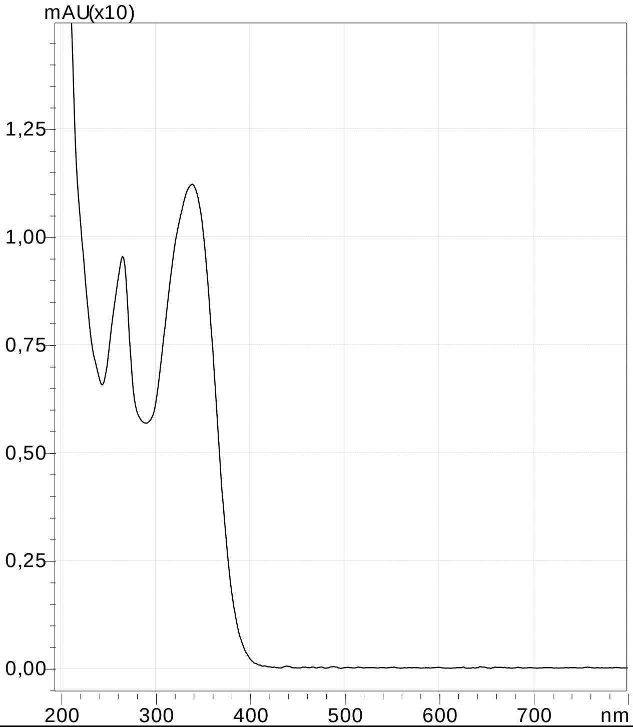
mAU(x10)
1,25
1,00
0,75
0,50
0,25
0,00
200
300
400
500
600
700
nm

<table>
<tr><td>57</td><td>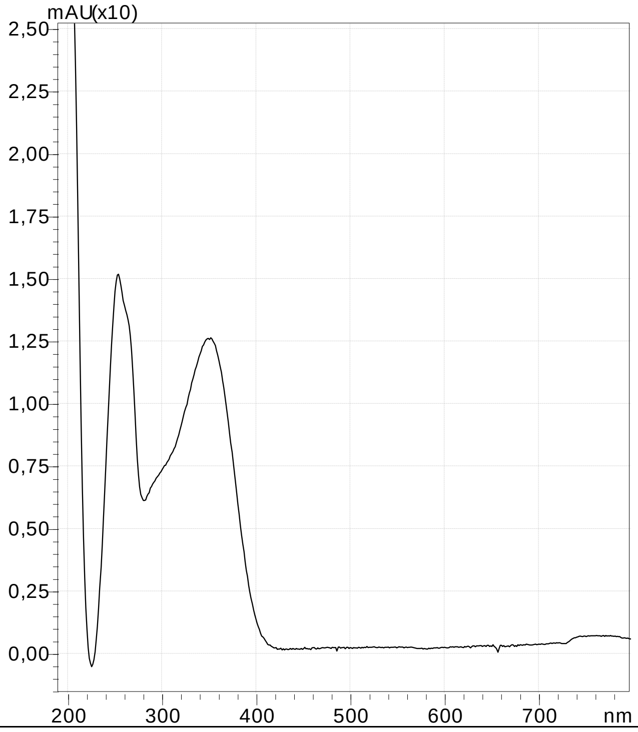
</td></tr>
<tr><td>58.</td><td>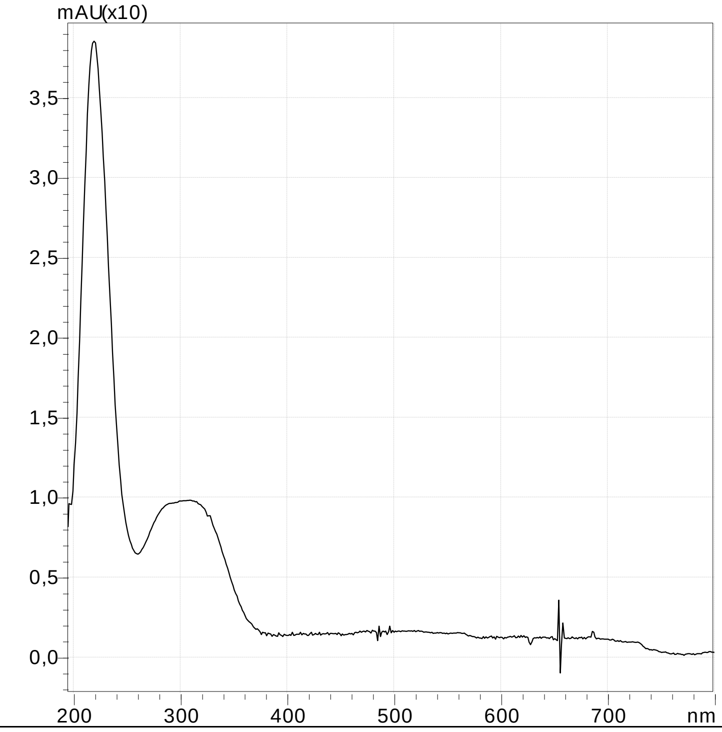
</td></tr>
</table>

**59.**	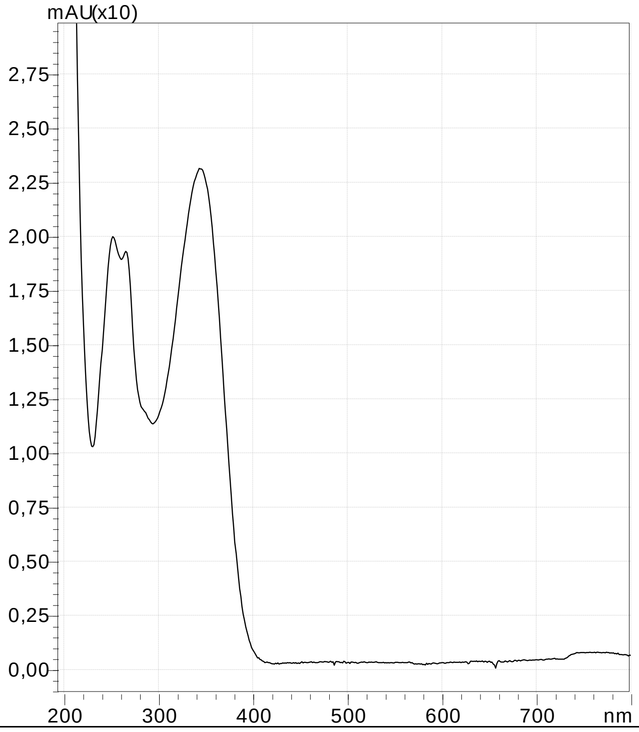mAU(x10)
2,75
2,50
2,25
2,00
1,75
1,50
1,25
1,00
0,75
0,50
0,25
0,00
200
300
400
500
600
700
nm
**60.**	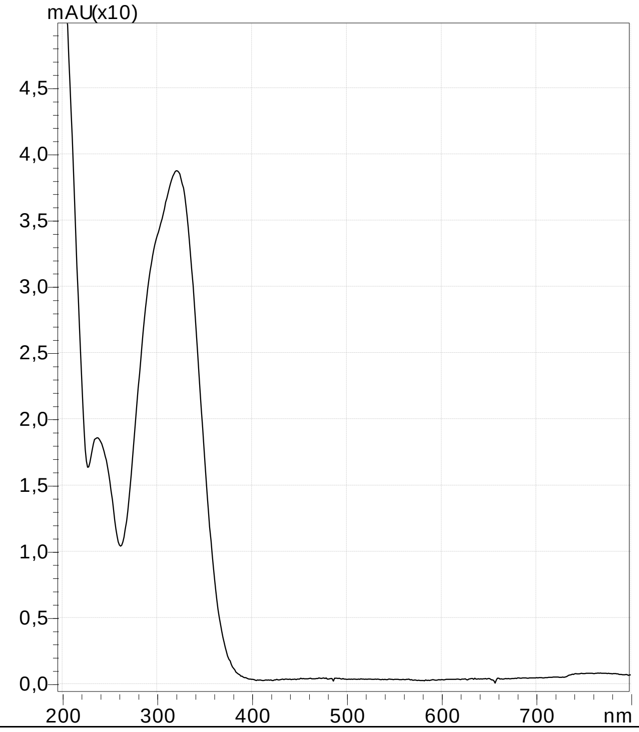mAU(x10)
4,5
4,0
3,5
3,0
2,5
2,0
1,5
1,0
0,5
0,0
200
300
400
500
600
700
nm

61.

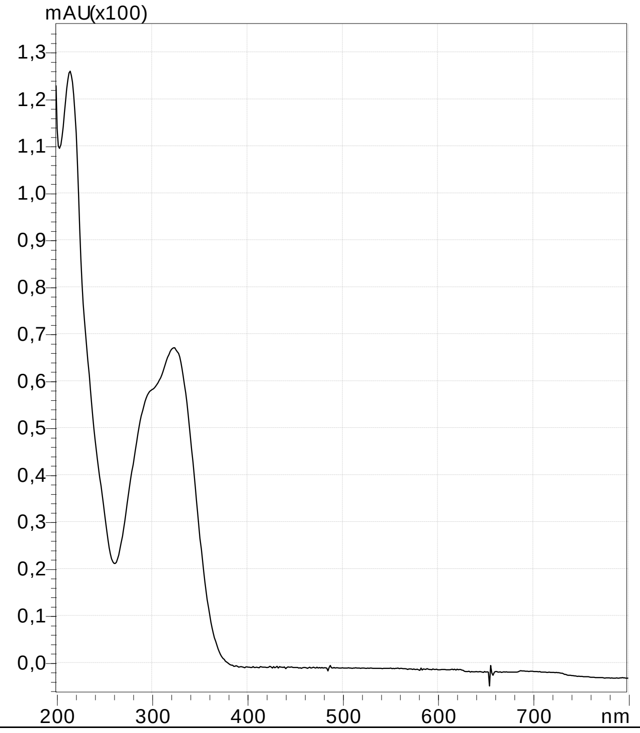
mAU(x100)
1,3
1,2
1,1
1,0
0,9
0,8
0,7
0,6
0,5
0,4
0,3
0,2
0,1
0,0
200
300
400
500
600
700
nm

62.

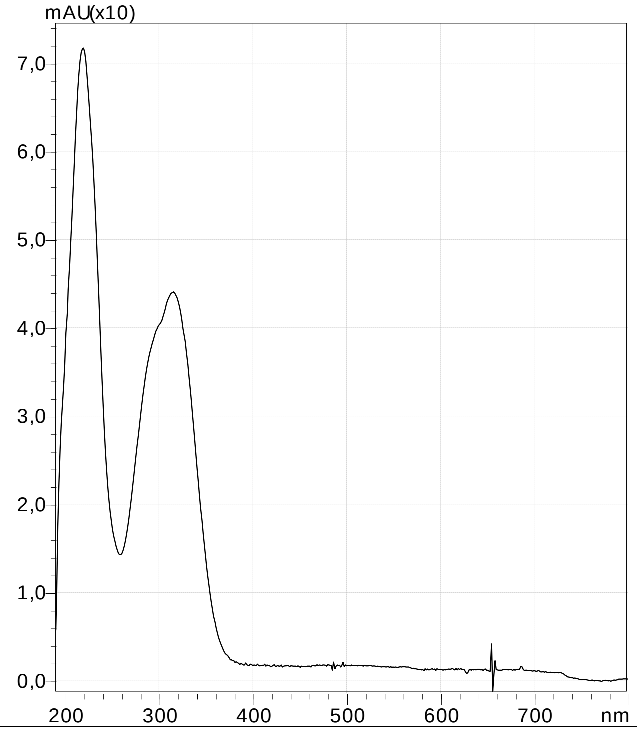
mAU(x10)
7,0
6,0
5,0
4,0
3,0
2,0
1,0
0,0
200
300
400
500
600
700
nm

**63.**	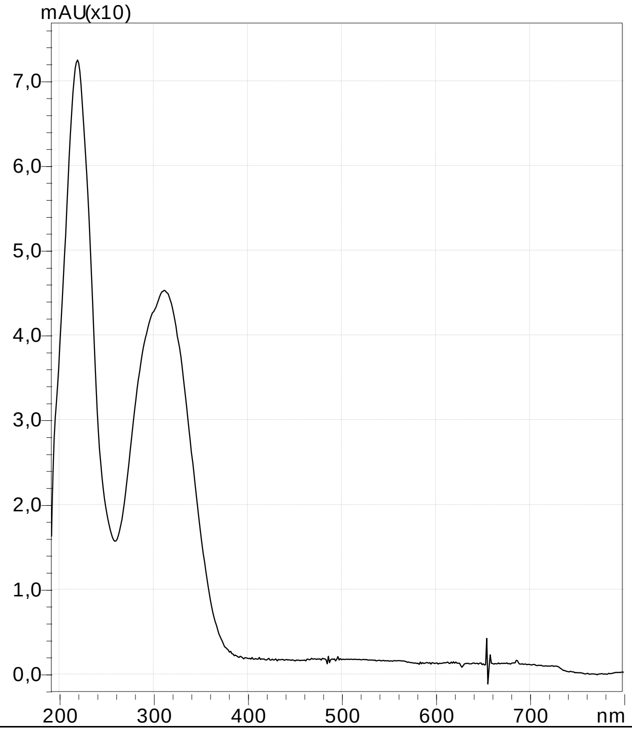 mAU(x10)<br>7,0<br>6,0<br>5,0<br>4,0<br>3,0<br>2,0<br>1,0<br>0,0<br>200 300 400 500 600 700 nm
**64.**	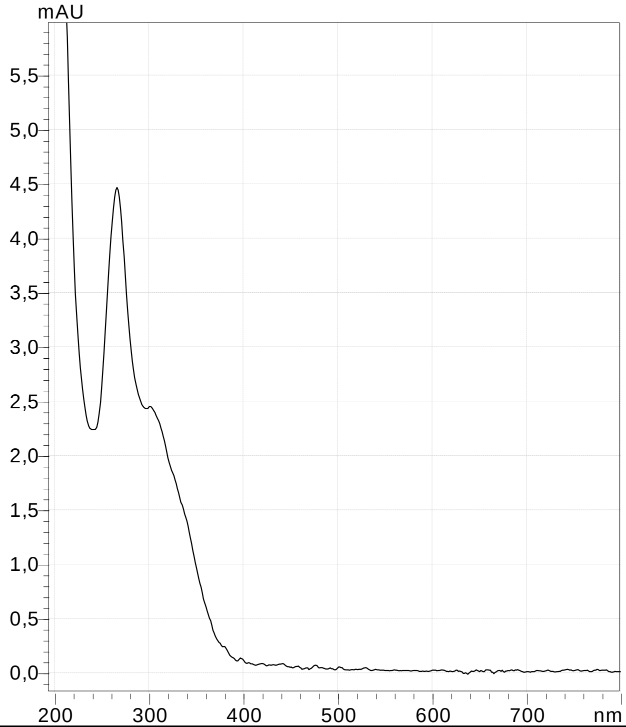 mAU<br>5,5<br>5,0<br>4,5<br>4,0<br>3,5<br>3,0<br>2,5<br>2,0<br>1,5<br>1,0<br>0,5<br>0,0<br>200 300 400 500 600 700 nm

**65.**	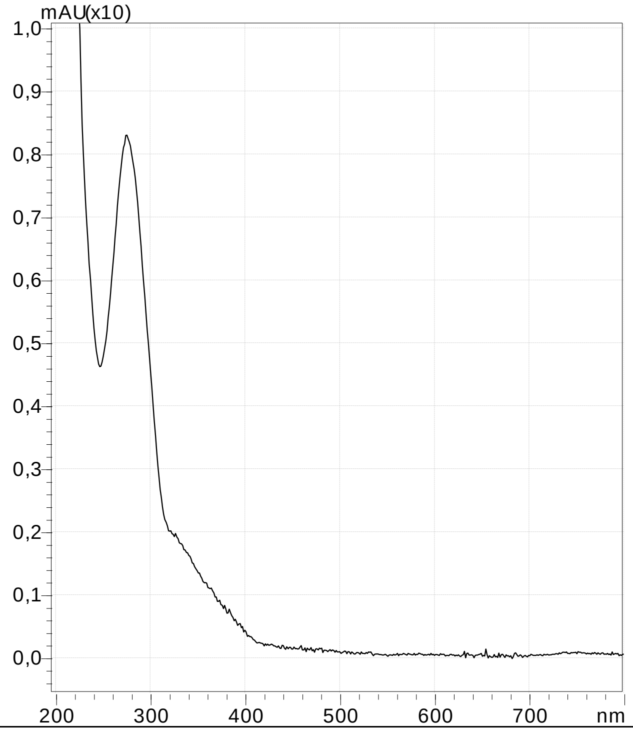mAU(x10) 1,0 0,9 0,8 0,7 0,6 0,5 0,4 0,3 0,2 0,1 0,0 200 300 400 500 600 700 nm
**66.**	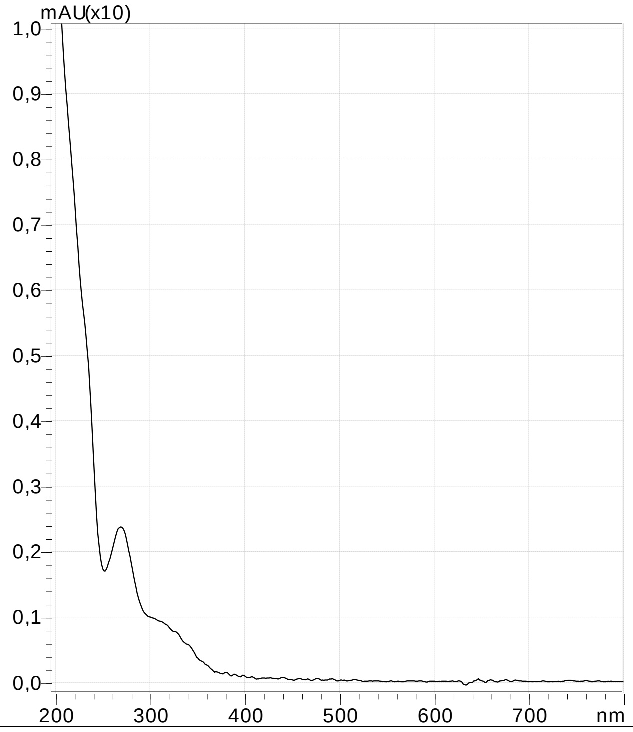mAU(x10) 1,0 0,9 0,8 0,7 0,6 0,5 0,4 0,3 0,2 0,1 0,0 200 300 400 500 600 700 nm

**67.**	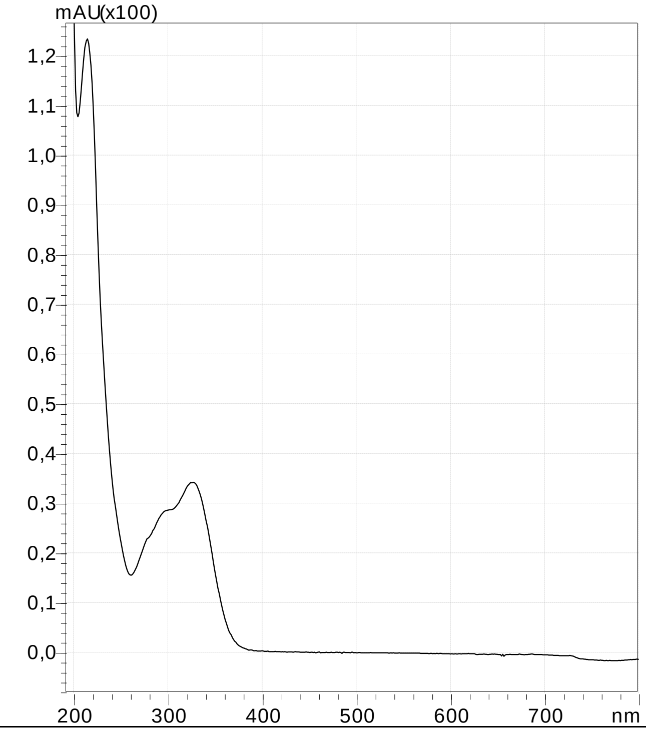mAU(x100)<br>1,2<br>1,1<br>1,0<br>0,9<br>0,8<br>0,7<br>0,6<br>0,5<br>0,4<br>0,3<br>0,2<br>0,1<br>0,0<br>200<br>300<br>400<br>500<br>600<br>700<br>nm
**68.**	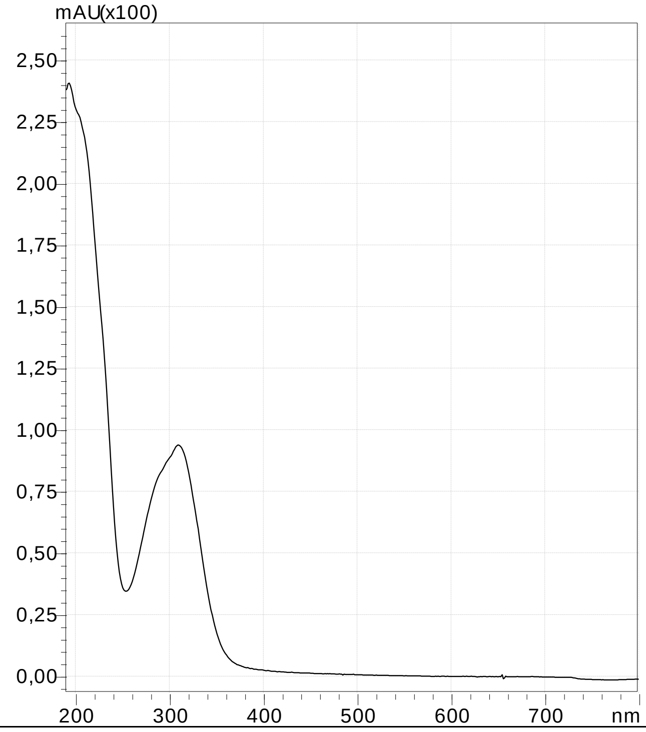mAU(x100)<br>2,50<br>2,25<br>2,00<br>1,75<br>1,50<br>1,25<br>1,00<br>0,75<br>0,50<br>0,25<br>0,00<br>200<br>300<br>400<br>500<br>600<br>700<br>nm

69.

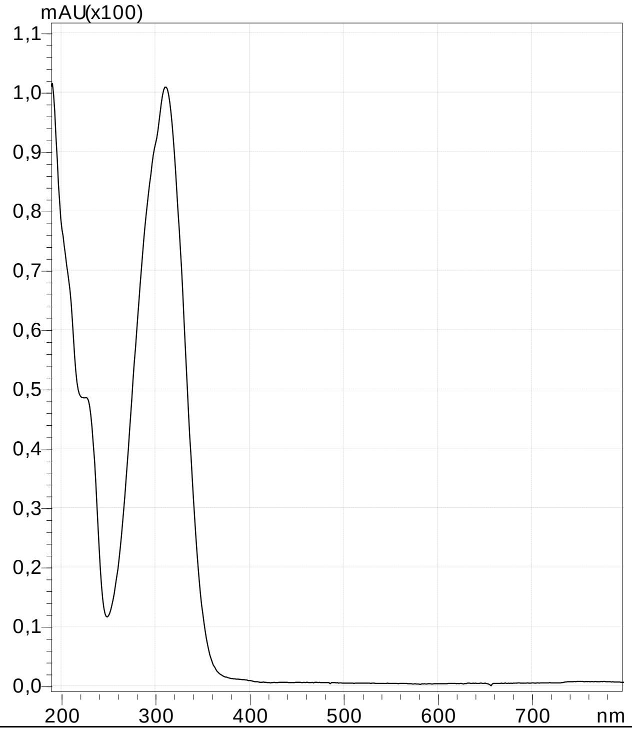
mAU(x100)
1,1
1,0
0,9
0,8
0,7
0,6
0,5
0,4
0,3
0,2
0,1
0,0
200
300
400
500
600
700
nm

70.

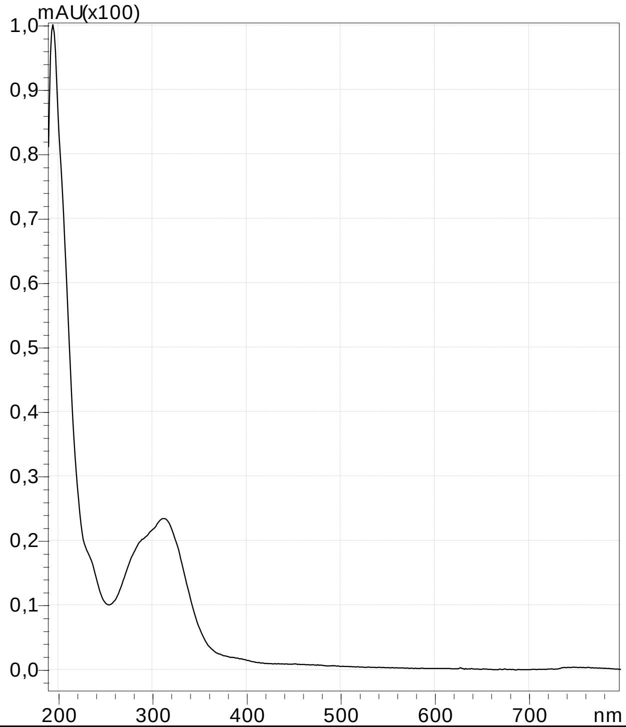
mAU(x100)
1,0
0,9
0,8
0,7
0,6
0,5
0,4
0,3
0,2
0,1
0,0
200
300
400
500
600
700
nm

**71.**	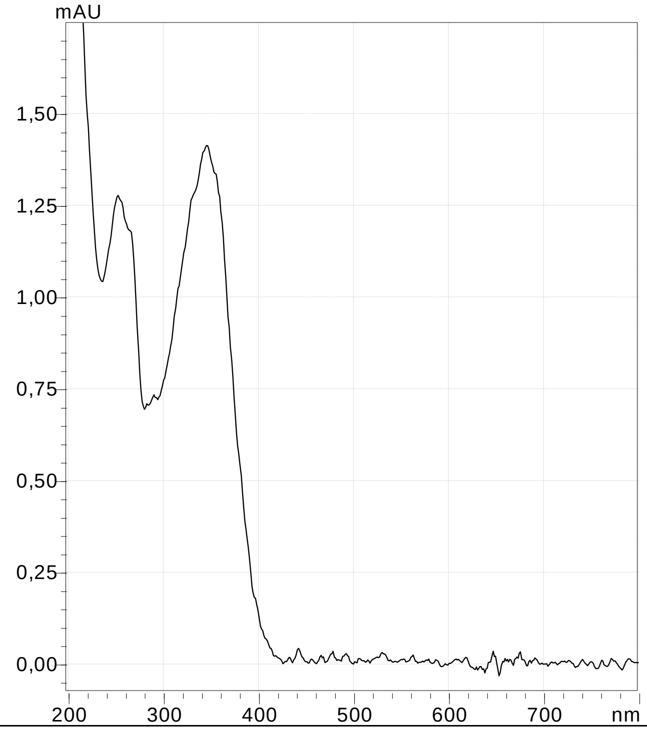mAU<br>1,50<br>1,25<br>1,00<br>0,75<br>0,50<br>0,25<br>0,00<br>200 300 400 500 600 700 nm
**72.**	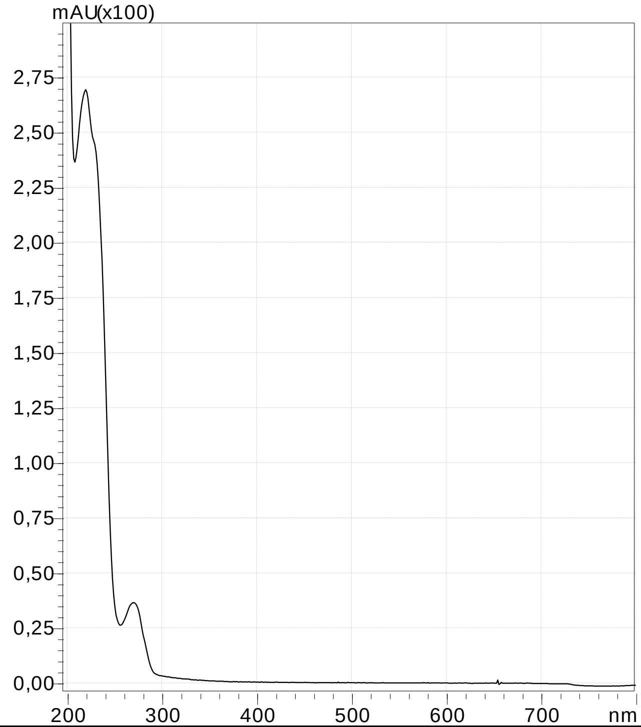mAU(x100)<br>2,75<br>2,50<br>2,25<br>2,00<br>1,75<br>1,50<br>1,25<br>1,00<br>0,75<br>0,50<br>0,25<br>0,00<br>200 300 400 500 600 700 nm

**73.**	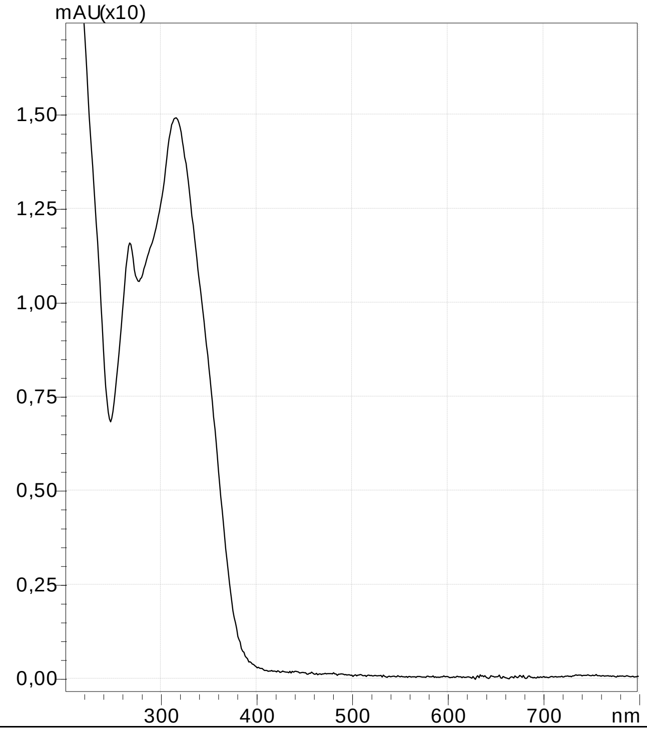mAU(x10)
1,50
1,25
1,00
0,75
0,50
0,25
0,00
300
400
500
600
700
nm
**74.**	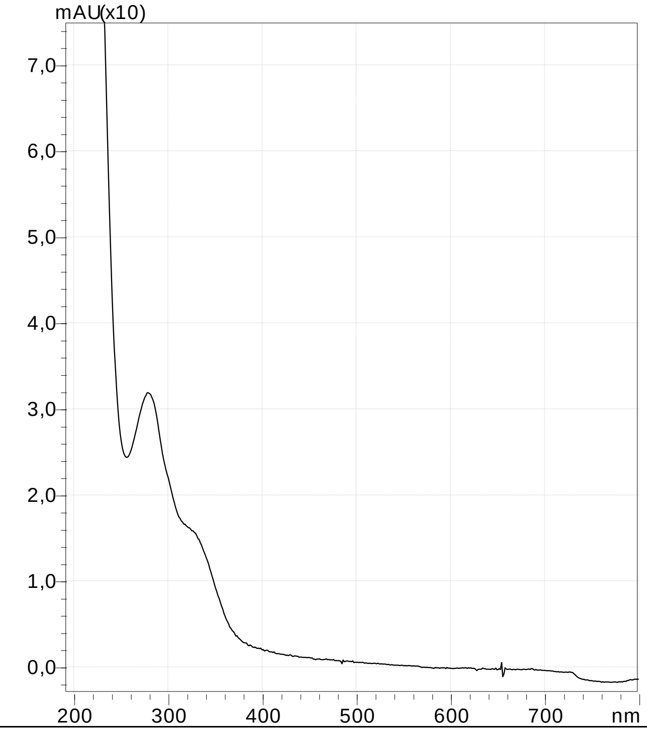mAU(x10)
7,0
6,0
5,0
4,0
3,0
2,0
1,0
0,0
200
300
400
500
600
700
nm

<table>
<tr><td>75.</td><td>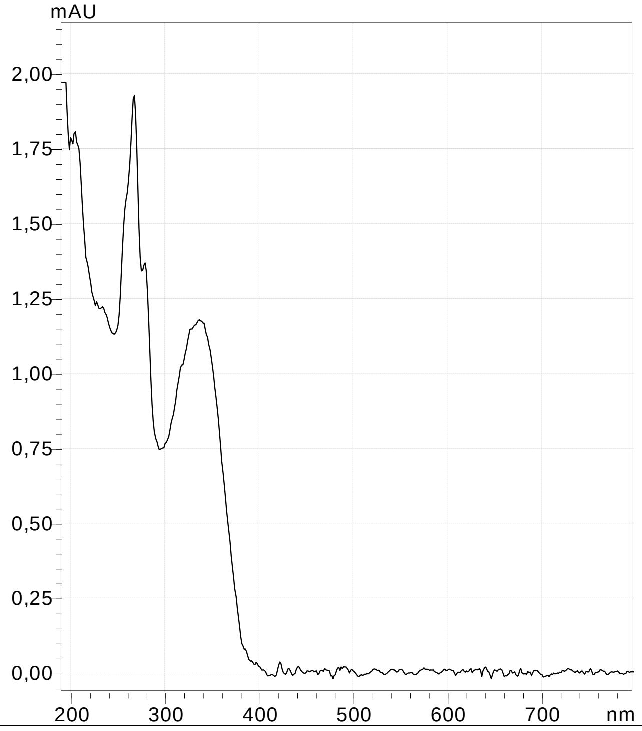
</td></tr>
<tr><td>76.</td><td>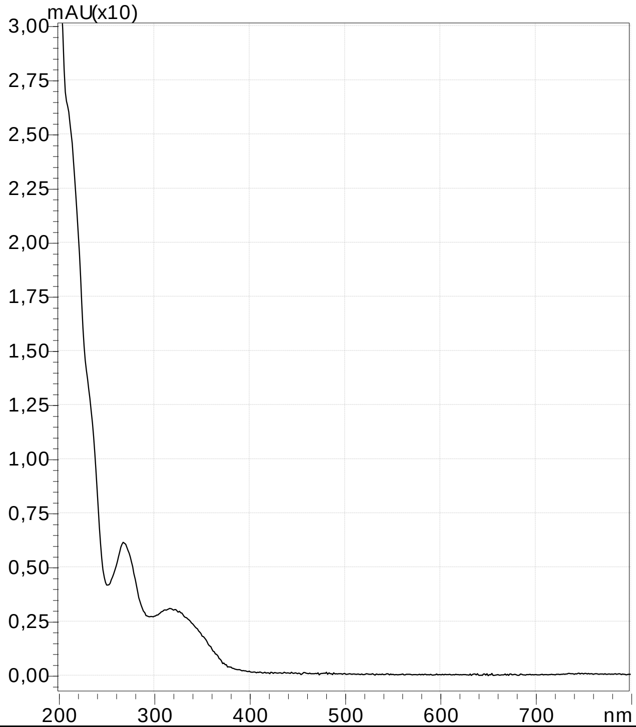
</td></tr>
</table>

**77.**	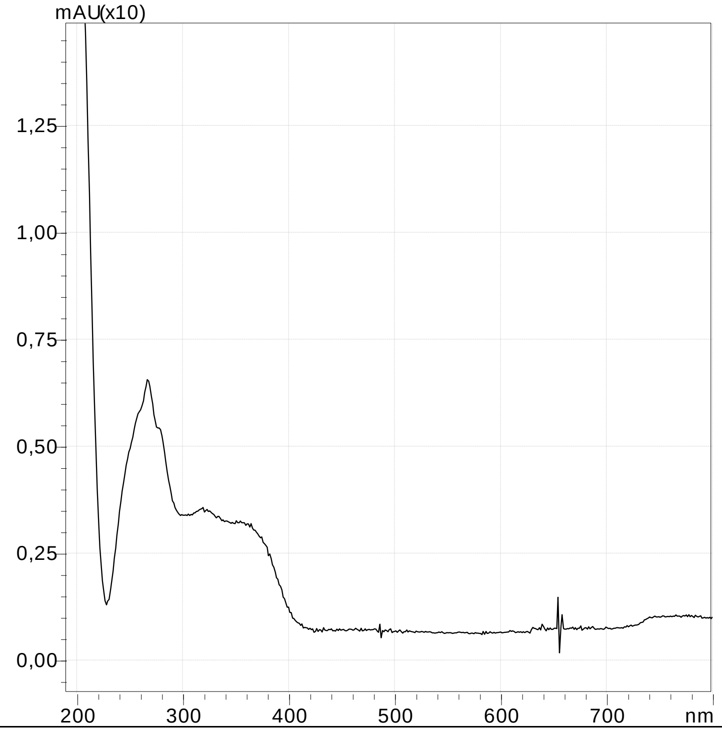mAU(x10)<br>1,25<br>1,00<br>0,75<br>0,50<br>0,25<br>0,00<br>200<br>300<br>400<br>500<br>600<br>700<br>nm
**78.**	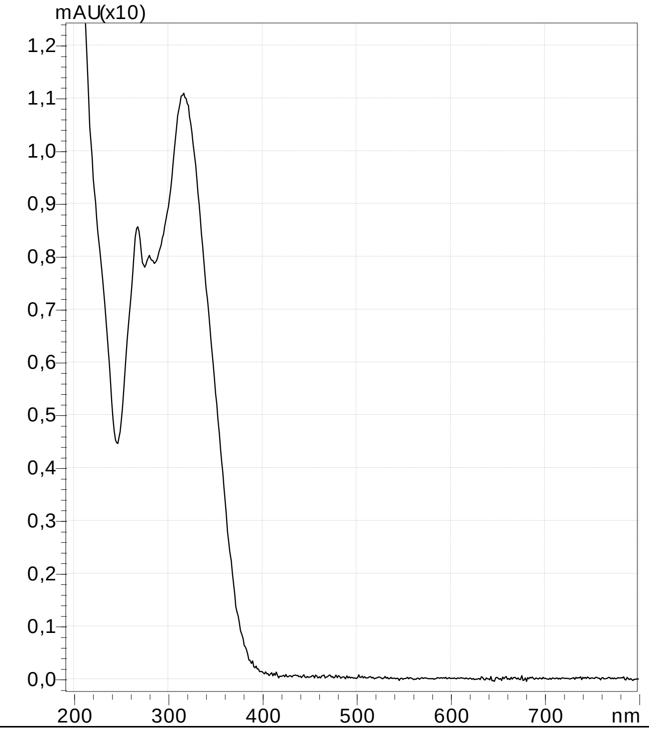mAU(x10)<br>1,2<br>1,1<br>1,0<br>0,9<br>0,8<br>0,7<br>0,6<br>0,5<br>0,4<br>0,3<br>0,2<br>0,1<br>0,0<br>200<br>300<br>400<br>500<br>600<br>700<br>nm

**79.**	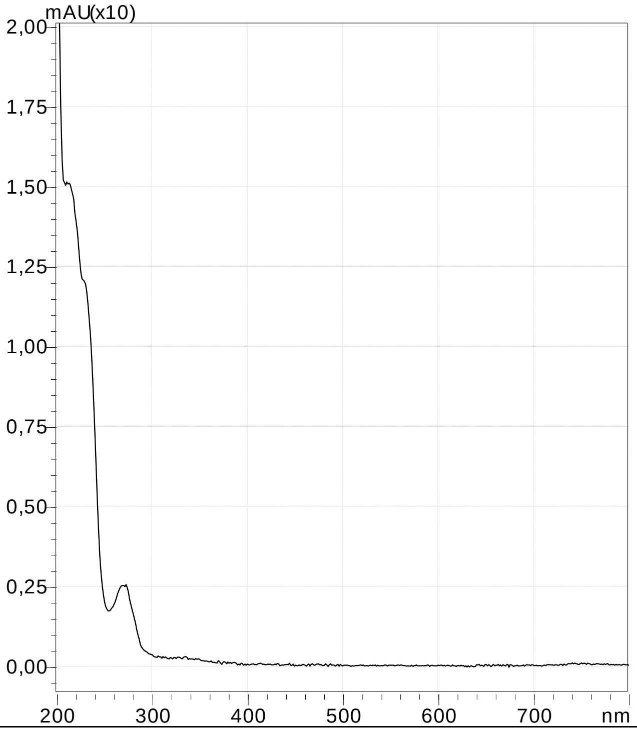mAU(x10)
2,00
1,75
1,50
1,25
1,00
0,75
0,50
0,25
0,00
200
300
400
500
600
700
nm
**80.**	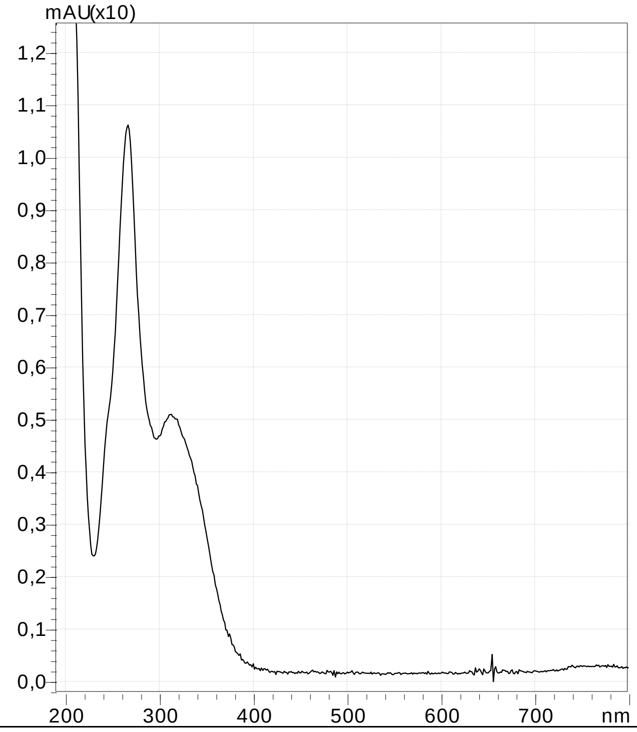mAU(x10)
1,2
1,1
1,0
0,9
0,8
0,7
0,6
0,5
0,4
0,3
0,2
0,1
0,0
200
300
400
500
600
700
nm

**81.**	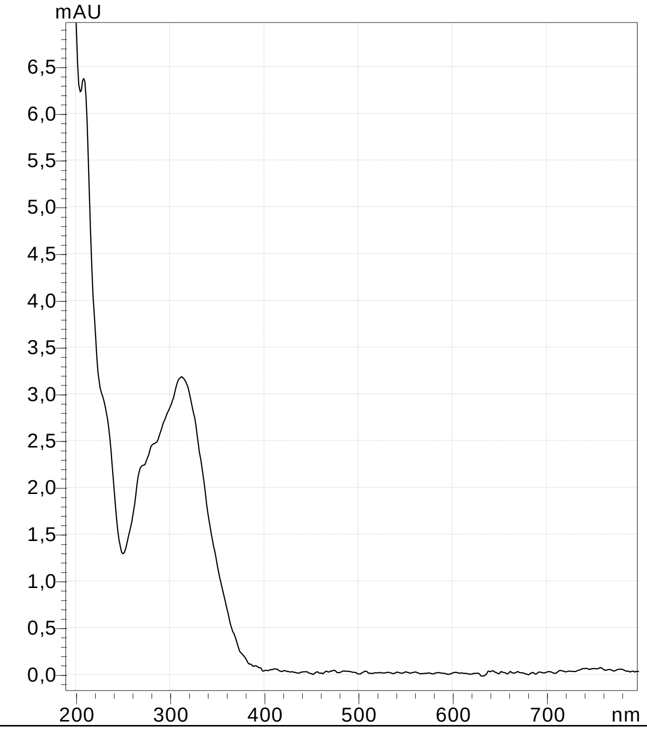mAU<br>6,5<br>6,0<br>5,5<br>5,0<br>4,5<br>4,0<br>3,5<br>3,0<br>2,5<br>2,0<br>1,5<br>1,0<br>0,5<br>0,0<br>200 300 400 500 600 700 nm
**82.**	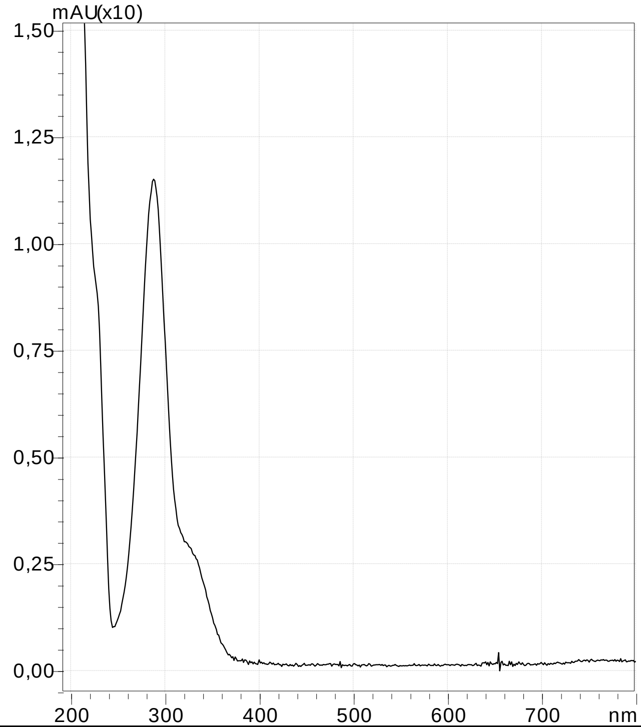mAU(x10)<br>1,50<br>1,25<br>1,00<br>0,75<br>0,50<br>0,25<br>0,00<br>200 300 400 500 600 700 nm

**83.**	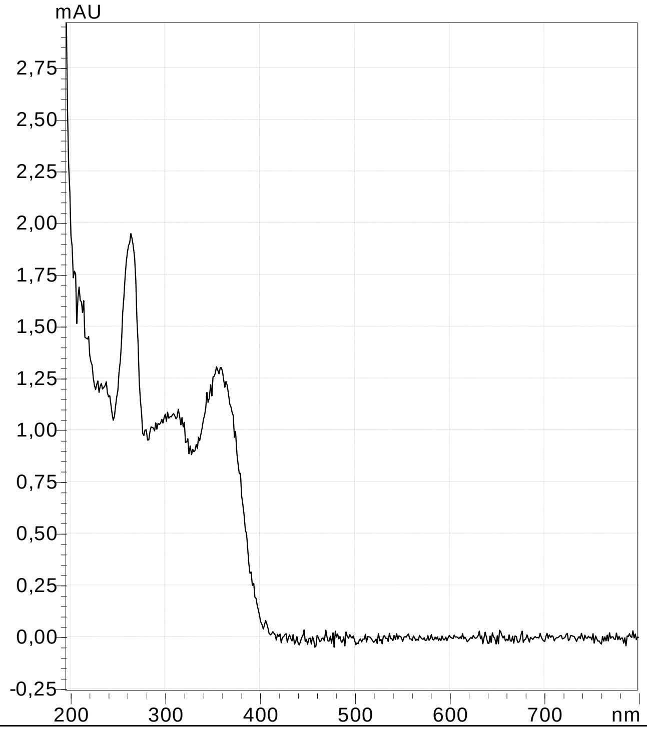mAU<br>2,75<br>2,50<br>2,25<br>2,00<br>1,75<br>1,50<br>1,25<br>1,00<br>0,75<br>0,50<br>0,25<br>0,00<br>-0,25<br>200<br>300<br>400<br>500<br>600<br>700<br>nm
**84.**	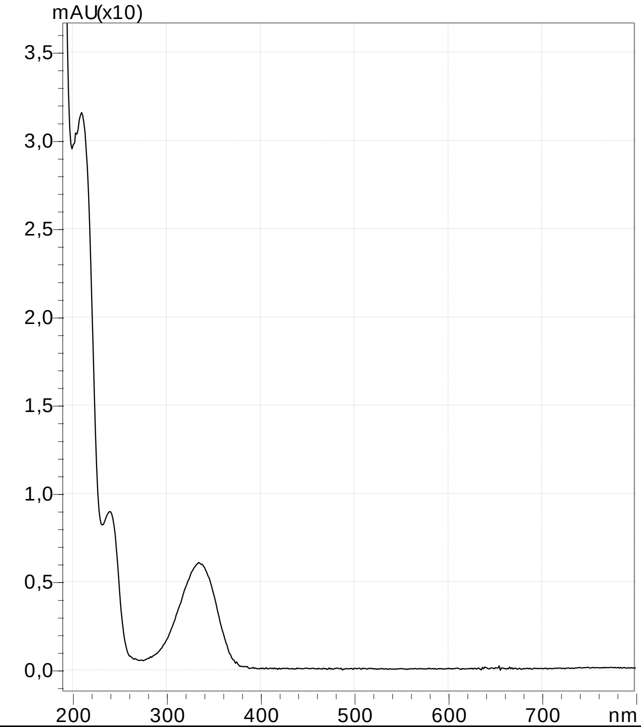mAU(x10)<br>3,5<br>3,0<br>2,5<br>2,0<br>1,5<br>1,0<br>0,5<br>0,0<br>200<br>300<br>400<br>500<br>600<br>700<br>nm

**85.**	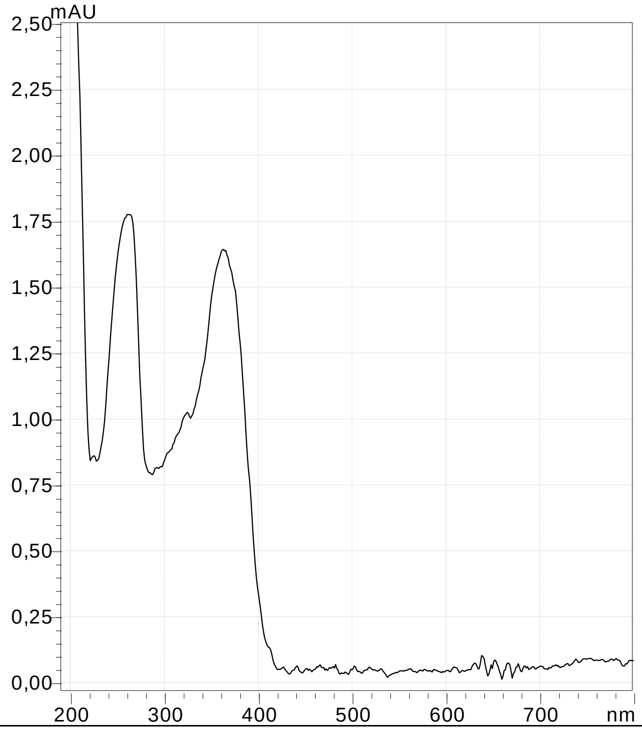mAU<br>2,50<br>2,25<br>2,00<br>1,75<br>1,50<br>1,25<br>1,00<br>0,75<br>0,50<br>0,25<br>0,00<br>200<br>300<br>400<br>500<br>600<br>700<br>nm
**86.**	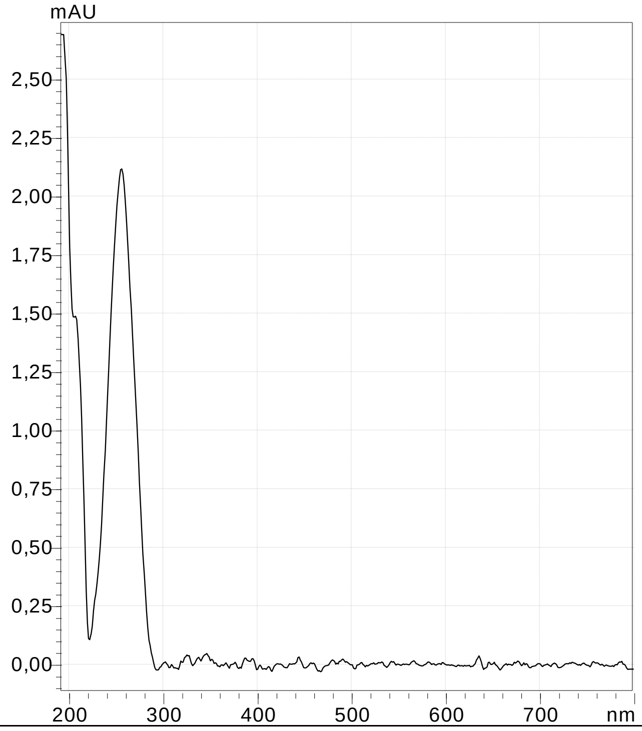mAU<br>2,50<br>2,25<br>2,00<br>1,75<br>1,50<br>1,25<br>1,00<br>0,75<br>0,50<br>0,25<br>0,00<br>200<br>300<br>400<br>500<br>600<br>700<br>nm
